# Cardiotoxicity of anthracycline agents for the treatment of cancer: Systematic review and meta-analysis of randomised controlled trials

**DOI:** 10.1186/1471-2407-10-337

**Published:** 2010-06-29

**Authors:** Lesley A Smith, Victoria R Cornelius, Christopher J Plummer, Gill Levitt, Mark Verrill, Peter Canney, Alison Jones

**Affiliations:** 1 Medical Research Matters, 77 Witney Road, Eynsham, OX29 4PN, UK; 2 Department of Cardiology, Freeman Hospital, Newcastle upon Tyne Hospitals NHS Foundation Trust, Newcastle upon Tyne, NE7 7DN, UK; 3 Great Ormond St Hospital for Sick Children NHS Trust, London, WCIN3JH, UK; 4 Northern Centre for Cancer Care, The Newcastle upon Tyne Hospitals NHS Foundation Trust, Freeman Hospital, High Heaton, Newcastle upon Tyne, NE7 7DN, UK; 5 Beatson West of Scotland Cancer Centre, Gartnavel General Hospital, Great Western Road, Glasgow, G12 0NY, UK; 6 UCLH Foundation Trust, Cancer Management, 3rd Floor West, 250 Euston Road, London NW1 2PG, UK

## Abstract

**Background:**

We conducted a systematic review and meta-analysis to clarify the risk of early and late cardiotoxicity of anthracycline agents in patients treated for breast or ovarian cancer, lymphoma, myeloma or sarcoma.

**Methods:**

Randomized controlled trials were sought using comprehensive searches of electronic databases in June 2008. Reference lists of retrieved articles were also scanned for additional articles. Outcomes investigated were early or late clinical and sub-clinical cardiotoxicity. Trial quality was assessed, and data were pooled through meta-analysis where appropriate.

**Results:**

Fifty-five published RCTs were included; the majority were on women with advanced breast cancer. A significantly greater risk of clinical cardiotoxicity was found with anthracycline compared with non-anthracycline regimens (OR 5.43 95% confidence interval: 2.34, 12.62), anthracycline versus mitoxantrone (OR 2.88 95% confidence interval: 1.29, 6.44), and bolus versus continuous anthracycline infusions (OR 4.13 95% confidence interval: 1.75, 9.72). Risk of clinical cardiotoxicity was significantly lower with epirubicin versus doxorubicin (OR 0.39 95% confidence interval: 0.20, 0.78), liposomal versus non-liposomal doxorubicin (OR 0.18 95% confidence interval: 0.08, 0.38) and with a concomitant cardioprotective agent (OR 0.21 95% confidence interval: 0.13, 0.33). No statistical heterogeneity was found for these pooled analyses. A similar pattern of results were found for subclinical cardiotoxicity; with risk significantly greater with anthracycline containing regimens and bolus administration; and significantly lower risk with epirubicin, liposomal doxorubicin versus doxorubicin but not epirubicin, and with concomitant use of a cardioprotective agent. Low to moderate statistical heterogeneity was found for two of the five pooled analyses, perhaps due to the different criteria used for reduction in Left Ventricular Ejection Fraction. Meta-analyses of any cardiotoxicity (clinical and subclinical) showed moderate to high statistical heterogeneity for four of five pooled analyses; criteria for any cardiotoxic event differed between studies. Nonetheless the pattern of results was similar to those for clinical or subclinical cardiotoxicity described above.

**Conclusions:**

Evidence is not sufficiently robust to support clear evidence-based recommendations on different anthracycline treatment regimens, or for routine use of cardiac protective agents or liposomal formulations. There is a need to improve cardiac monitoring in oncology trials.

## Background

Anthracyclines have been the key component of many cytotoxic regimens since their introduction in the 1960's and remain important in the treatment of many adult malignancies including breast cancer, sarcoma, lymphoma, and to a lesser extent, gynaecological cancer. In childhood cancers, anthracyclines are incorporated in more than 50% of regimens contributing to the overall survival rates in excess of 75%. For breast cancer long term survival is also greater than 70% across Europe [1]. This is certainly at least partly due to the widespread adoption of adjuvant anthracycline based chemotherapy after surgery for early breast cancer to reduce the risk of relapse and death.

For all cancers incidence increases with increasing age, therefore, increasing numbers of patients may have concomitant risk factors for cardiac disease at the time of diagnosis. In parallel, thresholds for offering treatment, including cytotoxic chemotherapy are becoming lower and survival is improving. This has resulted in more long term cancer survivors, including those who are cured and those with 'chronic' cancer requiring multiple drug interventions to control their disease. Longer survival highlights the importance of long term treatment related toxicity. Although paediatric malignancies are rare, the high cure rates achieved over the last two decades has highlighted the problems which may be encountered in adult life with relation to treatment induced late effects [2-4]. Anthracyclines are the drug class most closely associated with acute and late cardiac toxicity [5].

It has been known since the 1970s that anthracycline treatment is associated with an increased risk of heart failure, and that this is dependent on cumulative dose and schedule [6]. While the anti-cancer effects of anthracyclines are mediated primarily through inhibition of DNA synthesis, transcription and replication, they also generate oxygen-derived free radicals using iron as a co-factor and the mitochondrial respiratory chain. These free radicals cause direct damage to proteins, lipids and DNA and most available evidence suggests that myocyte apoptosis is related to increased oxidative stress caused by these processes [7]. However, cardiac myocytes do not increase in overall numbers after the postnatal period. Young adults have a mean of 8.2 billion myocyte nuclei [8], but around 52 million (0.6%) are lost each year, resulting in a 35% reduction in myocyte numbers during adult life. Ventricular wall thickness is maintained by increasing myocyte volume (110 μm^3^/year), but nevertheless there is an overall loss of ventricular mass of 0.9 g/year. With their very limited capacity for mitosis, any additional loss of myocytes will result in a permanent reduction in myocyte numbers, an increased reliance on adaptive mechanisms and increasing vulnerability during normal age-dependent cell loss.

## Rationale and aims of the systematic review

Many reviews of cardiotoxicity of chemotherapeutic agents have been published. However, these tend to have a very broad scope and cover many different agents and tend to be opinion pieces which may be biased in their selection and presentation of study results [5,9-11]. Several systematic reviews of the topic area have been published. These have a very specific focus on a particular type of cancer [12] or focus just on children [13,14]. Previous systematic reviews of anthracyclines and cardiotoxicity in adults have been conducted [15-17], however, they have a narrower focus than our review, and do not include detailed information on how cardiotoxicity outcomes were defined and measured. In addition, several relevant studies have since been published. The primary purpose of this review is to systemically analyze all available data from RCTs on the cardiac effects of anthracycline treatment for cancer in adults and children. To critically evaluate the cardiac risks associated with anthracyclines, and to demonstrate the current gaps in knowledge. This will facilitate design of future prospective studies collecting long-term data, and will allow accurate estimation of the lifetime risks and benefits of anthracycline treatment. In addition this review may inform long term surveillance programmes for those receiving anthracyclines.

## Methods

This systematic review was conducted according to a protocol based on published guidelines [18], available from the corresponding author upon request, and is reported according to the recent PRISMA guidelines [19] (see Additional file 1).

### Search strategy

Searches were conducted of Medline, EMBASE and the Cochrane Library in June 2008 using a combination of free text and thesaurus terms for individual anthracycline agents combined with terms for different tumour types, cardiotoxicity and randomised controlled trials (RCTs) (see Additional file 2). Additional studies were identified by screening reference lists of identified studies and reviews. Abstracts and posters were included only if they were reported in sufficient detail to enable full data extraction. Studies published in any language were eligible. Non-published articles were not sought.

### Selection criteria

Adults and children treated with an anthracycline for breast or ovarian cancer, sarcoma, non-Hodgkin's or Hodgkin's lymphoma and myeloma. The rationale for choosing these cancers was that they would be likely to have long-term survivors, and thus longer follow up on other important outcomes also. We excluded studies on participants with leukaemia due to the use of multiple chemotherapy agents known to cause cardiotoxicity confounding interpretation of results, and studies of lung and gastrointestinal (GI) cancers as they also have confounding factors with treatment coupled with poor long term outcomes making interpretation of results difficult.

Anthracyclines reviewed were: doxorubicin, epirubicin, daunorubicin and idarubicin. Mitoxantrone was included if it was compared with another anthracycline. RCTs comparing any anthracycline agent with another anthracycline agent in liposomal or non-liposomal formulation, or another non-anthracycline containing chemotherapy regimen were included. If chemotherapy regimens consisted of multiple agents, the treatment arms under comparison could only differ in the presence or absence of an anthracycline or type of anthracycline such that the other therapies being compared were the same between groups. Studies comparing an anthracycline in addition to a cardioprotective agent if compared with an anthracycline on its own were also included. All standard dosing regimens were included; we excluded high dose regimens. Any treatment duration was considered.

Any cardiotoxicity outcome including clinical and subclinical dysfunction was eligible for inclusion. We included cardiotoxicity evaluated using symptom checklists such as World Health Organisation (WHO) Common Toxicity Criteria (CTC) or New York Health Association (NYHA), cardiac function evaluated using MUGA scans and echocardiography and histological abnormalities by biopsy reported as Billingham scores. Both early and late cardiotoxicity were evaluated. We defined early toxicity as effects incurred during treatment or up to one year following treatment, and late toxicity as effects that occurred at least one year after treatment completion.

### Data extraction and outcomes

Two reviewers (LAS and VC) independently assessed whether individual studies met the inclusion criteria. Disagreements were resolved by discussion with co-authors. Reviewers were not blinded to the identities of the authors or institutions of the articles as this has not consistently been shown to affect the process (ref handbook).

Data from each trial report were recorded on a spreadsheet by one reviewer and checked by a second reviewer. For cardiotoxicity outcomes we recorded how they were defined, how they were evaluated, when they occurred (early or late) and whether they occurred on or off treatment. Where the timeframe for the outcome was not explicitly reported, we inferred this from average length of follow up in the trial. We used data for the intention-to-treat (ITT) population as defined by the authors of each study.

### Appraisal of trial quality

Each study was critically appraised for methodological quality using recognised criteria [20]. The reported design and conduct of each study were judged for four components that may introduce bias: method of generation of the random allocation, concealment of allocation at randomisation, blinding of trial participants and investigators, completeness of treatment and follow-up.

### Data synthesis

We categorised outcomes as clinical cardiotoxicity if the outcome was explicitly reported as congestive heart failure (CHF), and subclinical if the outcome was reported as a reduction in left ventricular ejection fraction (LVEF) or other abnormality in cardiac function determined using a diagnostic test. For outcomes not explicitly defined e.g. 'cardiotoxic event' we categorised them as a mixture of both clinical and subclinical. If sufficient data were available, summary estimates of treatment effects were produced using meta-analysis for each set of treatment comparisons. When the outcome was rare in one or more studies (< 3 events in each treatment arm or < 1% event rate) a Peto odds ratio (OR) fixed effects model was used for the meta-analysis which has been show to be the least biased method, and is a good approximation of the relative risk (RR) when the outcome is rare [21]. For outcomes that occurred more frequently, RR estimates were calculated and pooled using DerSimonian and Laird methods [22]. The I^2 ^was calculated to report the extent of heterogeneity detected [23]. The I^2 ^describes the proportion of statistical variability between studies in a meta-analysis that is greater than would be expected due to chance. Values in the region of 50% indicate moderate heterogeneity, and greater than 75% is considered substantial heterogeneity. Analyses were undertaken using the Stata V10.0 metan macro.

## Results

### Searches

**Figure 1:**
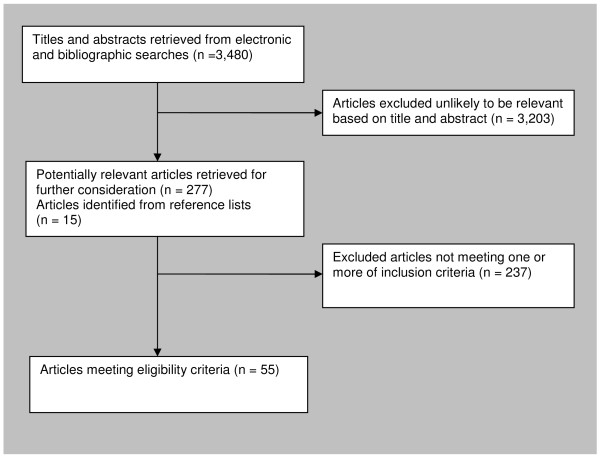
**Results of literature search and selection of randomised controlled trials for systematic review**.

Searches of electronic data bases identified 3,480 potentially relevant studies. After screening titles and abstracts, 277 full text reports were obtained and a further 15 studies were identified from reference lists of retrieved articles. Of these 292 articles, 55 RCTs met our inclusion criteria (Figure 1). The 237 articles excluded are listed in Additional file 3; Table S1.

### Characteristics of included studies

We included 55 RCTs reporting the treatment comparisons listed below. Four RCTs had three treatment groups, therefore, contributed data to more than one comparison:

• One anthracycline agent compared with either mitoxantrone (15) or a non-anthracycline containing chemotherapy regimen (8)

• Anthracycline intermittent/bolus dosing compared with continuous infusion (4)

• One anthracycline agent compared with another anthracycline (20)

• Anthracycline plus a cardioprotective agent compared with anthracycline alone or with placebo (12)

The majority of the studies were on women with advanced or metastatic breast cancer. Twenty-one studies included participants with myeloma, lymphoma, sarcoma or ovarian cancer, and three studies included a mixture of tumour types (Additional file 4; Table S2). Most studies specifically excluded patients with existing cardiac dysfunction; nonetheless, some patients had risk factors for poorer cardiac outcomes. These were mainly due to prior treatment with an anthracycline or radiotherapy. Nine studies specifically reported how many participants had radiotherapy to the chest - none reported if it had been delivered to the left side of the chest (Additional file 5; Table S3). Some (n = 22) studies specifically recruited patients with no prior anthracycline treatment, fewer (n = 11) recruited patients that were treatment naive. These tended to be the studies on people with lymphoma and osteosarcoma (Additional file 4; Table S2). Few pediatric studies (n = 4) met the inclusion criteria as the majority of cardiotoxicity studies have been performed on children with acute leukaemia [13].

### Quality assessment

The quality of the included trials was variable ranging from poor to high quality. Common limitations included: insufficient details to adequately assess fidelity of randomization, lack of blinding of outcome assessors, cardiotoxicity outcomes only reported in sub-sets of patients - not the whole randomised sample, and inadequate assessment and reporting of cardiotoxicity results, particularly in studies were this was not a primary endpoint. Definitions used for different cardiotoxicity outcomes varied from one study to another, the definition used was not always reported and the time point when outcomes occurred was not always clear (Additional file 6; Table S4 a-f). In addition, sample sizes were often too small to provide reliable estimates of relatively rare adverse events such as CHF and cardiac related death.

#### One anthracycline compared with either a non-anthracycline chemotherapy regimen or mitoxantrone

Eight studies compared an anthracycline with a non-anthracycline in women with breast cancer [24-27], ovarian cancer [28], children with lymphoma [29,30] and adults and children with osteosarcoma [31]. Treatment regimens varied across studies, and doxorubicin, epirubicin or daunomycin were given with other chemotherapy agents. Cardiotoxicity outcomes occurred early and on treatment [24,27,28]; late and off treatment [26,29,30], and some may have occurred late and off treatment [25,31].

**Figure 2:**
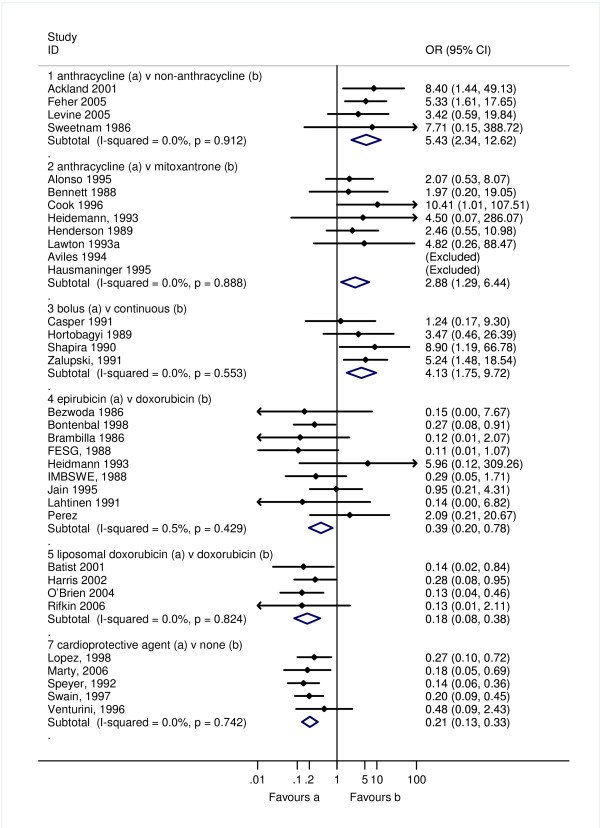
**Clinical cardiotoxicity defined as incidence of CHF in RCTs comparing different treatment regimens**. The open diamond represents the pooled Peto Odds Ratio and 95% confidence interval (CI) for each treatment comparison. I-squared represents the proportion of variability between studies in excess of that expected due to chance, and p = probability that differences between study estimates are due to chance.

**Figure 3:**
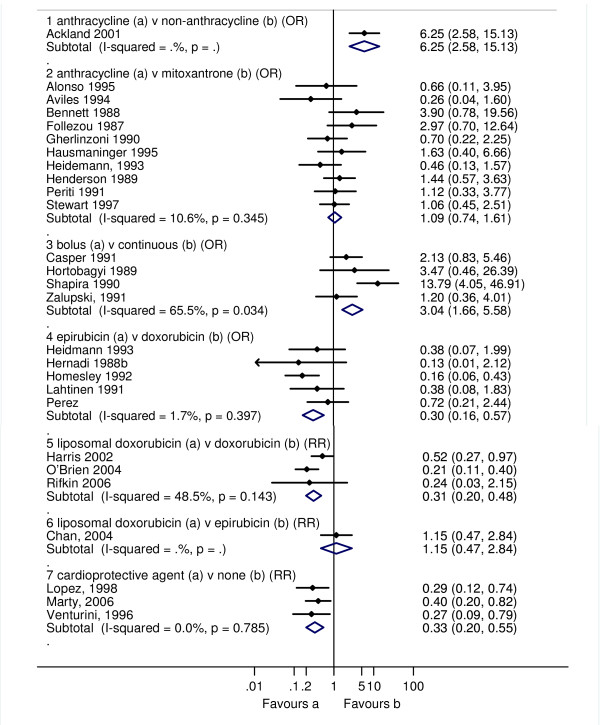
**Sub clinical cardiotoxicity defined as reduction LVEF in RCTs comparing different treatment regimens**. The open diamond represents the pooled Peto Odds Ratio and 95% CI for treatment comparisons 1-4, and relative risk (RR) with 95% CI for comparisons 5-7. I-squared represents the proportion of variability between studies in excess of that expected due to chance, and p = probability that the differences between study estimates are due to chance.

**Figure 4:**
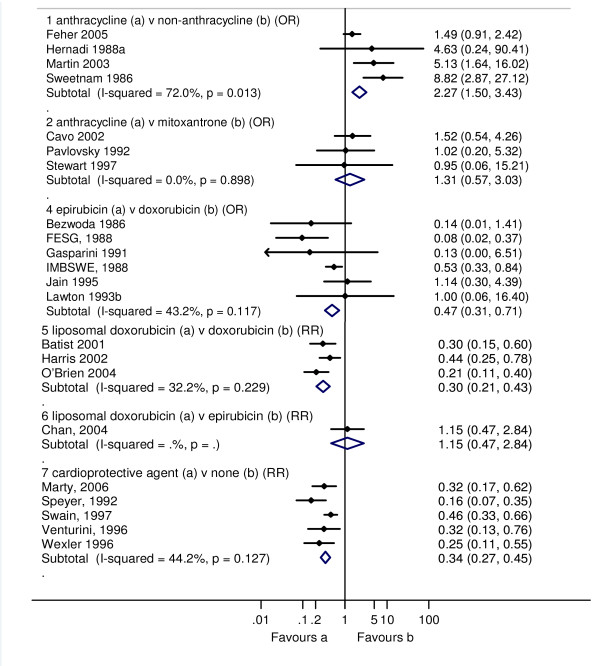
**Clinical and subclinical cardiotoxicity in RCTs where cardiotoxicity outcomes could not be categorised as one or the other**. The open diamond represents the pooled Peto Odds Ratio and 95% CI for treatment comparisons 1, 2 and 4, and relative risk (RR) with 95% CI for comparisons 5-7. I-squared represents the proportion of variability between studies in excess of that expected due to chance, and p = probability that differences between study estimates are due to chance.

Three studies reported the incidence of CHF, and one study reported discontinuation due to reduction is LVEF. We made a post-hoc decision to categorise the discontinuation due to reduction in LVEF as a clinical cardiotoxic event due to the implied harm being greater than for a reduction in LVEF alone (sub-clinical). An anthracycline containing regimen increased the risk of clinical cardiotoxicity -OR 5.43 (95% confidence interval (CI): 2.34, 12.62; p < 0.0001; I^2 ^= 0%); and subclinical cardiotoxicity OR 6.25 (95% CI: 2.58, 15.13; p < 0.0001). For any cardiotoxic event (clinical and sub-clinical) the pooled result is highly dependent on the choice of summary statistic; the Peto OR showed a significant increase in odds of cardiotoxicity with anthracycline compared with non-anthracycline: 2.27 (95% CI: 1.50, 3.43, p < 0.0001), whereas, the RR while higher, was not statistically significant, 4.23 (95% CI: 0.93, 19.38; p = 0.08). One possible explanation for these differences in effects is due to the different definitions of a cardiotoxic event used in each study giving rise to substantial heterogeneity, I^2 ^72.0% (Figures 2, 3 and 4).

Meta-analysis of four studies showed cardiac related deaths, though infrequent, were significantly higher with an anthracycline OR 4.94 (95% CI: 1.23, 19.87; p = 0.025; I^2 ^= 19.3%).

Fifteen studies compared an anthracycline with mitoxantrone in women with advanced or metastatic breast cancer [32-42], multiple myeloma [43], non-Hodgkin's lymphoma [44], Hodgkin's lymphoma and lymphoma [45,46]. Treatment regimens varied across studies and doxorubicin or epirubicin were given either as single agents or with other chemotherapy agents. Cardiotoxicity outcomes occurred early and on treatment [39,41]; in the remaining studies it was unclear when the cardiotoxicity outcomes occurred and may include both early and late outcomes.

An anthracycline containing regimen increased the risk of clinical cardiotoxicity OR 2.88 (95% CI: 1.29, 6.44; p = 0.01; I^2 ^= 0%) compared with a chemotherapy regimen containing mitoxantrone. There was little difference between the treatment groups for subclinical cardiotoxicity, and for any cardiotoxic event (clinical and sub-clinical): OR 1.09 (95% CI: 0.74, 1.61; p = 0.673; I^2 ^= 10.6%) and OR 1.31 (0.57, 3.03; p = 0.521; I^2 ^= 0%), respectively. Cardiac related deaths were reported by only one study in one patient in the doxorubicin group (Figures 2, 3 and 4).

#### Anthracycline intermittent/bolus compared with continuous infusion

Four RCTs compared bolus with a continuous infusion in women with advanced or metastatic breast and breast or ovarian cancer [47,48], and adults with recurrent or metastatic soft tissue sarcoma [49,50]. One study included participants previously treated with an anthracycline with unspecified cardiac risk factors in 10/25 (40%) and 15/25 (60%) of women in bolus and continuous groups, respectively [47]. Patients with known cardiac problems were excluded from the other studies. Duration of continuous infusion schedules were: 6, 48, 72 and 96 hours; cumulative doses of anthracyclines received are shown in Additional file 4; Table S2.

Cardiotoxicity outcomes reported in the RCTs are shown in Additional file 6; Table S4. It was not clear when the outcomes occurred in one study [50], and it is possible some events could be classified as late as median follow-up was five years; for the other studies outcomes occurred early and on treatment.

Epirubicin or doxorubicin given as a bolus significantly increased the risk of clinical cardiotoxicity, OR 4.13 (1.75, 9.72; p = 0.001; I^2 ^= 0%), and subclinical cardiotoxicity OR 3.04 (1.66, 5.58; p < 0.0001; I^2 ^= 65.5%), compared with continuous infusion (Figures 2, 3 and 4). For subclinical cardiotoxicity the pooled result was highly dependent on the choice of summary statistic - random effects RR 1.93 (95% CI: 0.84, 4.44). One possible explanation for the difference in the effect estimates is the different definition for LVEF reduction used in each study giving rise to substantial heterogeneity. Cardiac related deaths were infrequent in either treatment group in two studies (Additional file 6; Table S4).

There were no significant differences in response rates, remission or survival among patients in each study according to treatment group.

#### One anthracycline compared with another

Thirteen studies compared doxorubicin with epirubicin. The majority of the studies were on women with advanced or metastatic breast cancer [38,40,51-57], three were on women with ovarian cancer [28,58,59], and one studied patients with non-Hodgkin's lymphoma [60]. Some participants had cardiac risk factors at baseline, mainly due to prior treatment with an anthracycline or radiotherapy (Additional file 6; Table S4).

Cardiotoxicity outcomes reported in the studies are shown in Additional file 6; Table S4. Outcomes occurred early and on treatment in five studies; in the remaining it was not clear when they occurred and it is possible some events could be classified as late cardiotoxicity as follow-up was greater than one year.

Epirubicin significantly decreased the risk of clinical cardiotoxicity OR 0.39 (0.20, 0.78; p = 0.008; I^2 ^= 0.5%), subclinical cardiotoxicity OR 0.30 (0.16, 0.57; p < 0.0001; I^2 ^= 1.7%) and any cardiotoxic event (clinical and sub-clinical) OR 0.47 (0.31, 0.71; p < 0.0001; I^2 ^= 43.2%) compared with doxorubicin (Figures 2, 3 and 4). Cardiac related deaths were infrequent in either treatment group. There was no evidence of a difference in tumour response rate or survival between epirubicin and doxorubicin.

Four studies compared liposomal doxorubicin with conventional doxorubicin in women with metastatic breast cancer [61-63], and in men and women with multiple myeloma [64]. In the study of previously untreated patients with multiple myeloma, cardiac risk factors were absent. In two of the studies some women had cardiac dysfunction at baseline and in one, cardiac dysfunction was absent at baseline, however, risk factors due to prior treatment with chemotherapy and/or radiotherapy were present in some of the participants (Additional file 5; Table S3). One study compared liposomal doxorubicin with epirubicin in women with metastatic breast cancer [65]. Women had no cardiac dysfunction at baseline; however risk factors for cardiac dysfunction were present in some women due to prior radiotherapy to the chest and prior chemotherapy (Additional file 5; Table S3). The cardiotoxicity outcomes occurred early and on treatment (2 studies); in the others it was not clear when they occurred and it is possible some events could be classified as late (3 studies).

Liposomal doxorubicin compared with conventional doxorubicin decreased the risk of clinical cardiotoxicity OR 0.18 (0.08, 0.38; p < 0.0001; I^2 ^= 0%), subclinical cardiotoxicity RR 0.31 (0.20, 0.48; p < 0.0001; I^2 ^= 48.5%) and any cardiotoxic event (clinical and sub-clinical) RR 0.30 (0.21, 0.43; p < 0.0001; I^2 ^= 32.2%). There was little difference between liposomal doxorubicin and epirubicin for subclinical cardiotoxicity, and any cardiotoxic event RR 1.15 (0.47, 2.84; p = 0.754); no cases of CHF were reported in either group (Figure 2, 3 and 4). Cardiac related deaths were infrequent in any treatment group. There was no evidence that suggests a difference in tumour response rate or survival between liposomal doxorubicin and doxorubicin, and whilst time to treatment failure and time to disease progression were significantly longer with liposomal doxorubicin than with epirubicin at equimolar concentrations, survival was comparable.

One study each of doxorubicin [66] and epidoxorubicin [67] compared with idarubicin found little difference in risk of any cardiotoxic event (clinical and sub-clinical) with comparable therapeutic efficacy in patients with non-Hodgkin's lymphoma.

#### Anthracycline plus a cardioprotective agent compared with an anthracycline

Six RCTs evaluated the cardioprotective agent dexrazoxane; four on women with advanced breast cancer [68-71], one on young people aged no more than 25 years with sarcoma [72], and one with adults with breast cancer or sarcoma [72,73]. The ratio of dexrazoxane to anthracycline varied between studies from 10:1 to 20:1. Cumulative doses of anthracyclines received were similar between randomised groups (Additional file 4; Table S2). Risk factors for cardiotoxicity were present in some participants, largely due to prior treatment with either anthracyclines or radiotherapy to the chest area; none had cardiac dysfunction at baseline, (Additional file 5; Table S3). For two of the studies outcomes occurred early and on treatment; in the other four studies it was not clear when the outcomes occurred.

Dexrazoxane given either with doxorubicin or epirubicin significantly reduced the risk of clinical cardiotoxicity OR 0.21 (0.13, 0.33; p < 0.0001; I^2 ^= 0%), subclinical cardiotoxicity RR 0.33 (0.20, 0.55; p < 0.0001; I^2 ^= 0%) and any cardiotoxic event (clinical and sub-clinical) RR 0.34 (0.27, 0.45; p < 0.0001; I^2 ^= 44.2%) compared with doxorubicin or epirubicin with no cardioprotective agent. Cardiac related deaths were infrequent in either treatment group (Figure 2, 3 and 4). Meta-analysis of two studies showed no significant difference between groups, OR 0.39 (95% CI: 0.05, 2.76; p = 0.343).

For carvedilol [74], L-cartinine [75], prenylamine [76], amifostine [77] and acetylcysteine [78] there was only one RCT for each agent. No significant differences in cardiotoxic outcomes were detected between treatment groups.

Additional outcomes not suitable for meta-analysis are shown in (Additional file 6; Table S4.

The potential modifying effect of specific risk factors for cardiac outcomes at baseline remains an unanswered question in these RCTs. The presence or absence of specific risk factors was not consistently reported, and were not always balanced between treatment arms in the trials, potentially affecting results. We were unable to investigate the impact of specific risk factors as data were not reported separately for patients with and without specific characteristics. Generally there were insufficient studies in each pooled analyses to conduct meaningful subgroup analyses of different groups of trials according to the presence or absence of a specific risk factor such as prior anthracycline use or radiotherapy treatment.

## Discussion

Our analyses demonstrate that anthracyclines increased the risk of clinical cardiotoxicity by 5.43 fold, subclinical cardiotoxicity by 6.25 fold, any cardiotoxicity by 2.27 fold and the risk of cardiac death by 4.94 fold compared with non-anthracycline regimens. For clinical cardiotoxicity, the risk was 4.13 fold higher with bolus administration compared to continuous infusion, and was 61% lower with epirubicin compared to doxorubicin. The risk was also 22% lower with liposomal doxorubicin, which allows a more favourable tumour to normal tissue concentration ratio. Risk was also 79% lower with the use of the cardioprotective agent dexrazoxane, which is an iron chelating agent and is thought to decrease the cardiotoxic effect of doxorubicin through preventing free radical formation [79]. These data however do not allow us to comment on the absolute risks of early or late cardiac events after anthracyclines from this heterogeneous group of patients.

Despite an extensive literature search only 55 out of the 292 papers for which full text articles were obtained were eligible for this review. This reflects the focus of most oncology research on cause specific outcomes in relation to cancer, and only acute outcomes in relation to toxicity. The majority of the 55 included papers were on women with advanced breast cancer. As many of the risk factors for breast cancer are common to cardiovascular disease, the fact that breast cancer is numerically the largest group in this review may bias the results towards overestimating the cardiac risks, unless other competing co-morbidities are fully controlled for. Many patients with breast cancer had received prior chemotherapy, and only some patients with sarcoma, lymphoma and paediatric malignancy were treatment naïve. Within the breast cancer population, some patients had cardiac risk factors, of particular concern; the impact of left sided chest wall radiotherapy could not be assessed.

The quality of the papers in terms of determining cardiac outcome was variable and confounded by sample sizes which are inadequate to accurately estimate rare outcomes such as cardiotoxicity. Further limitations included the fact that they were reported only in subsets of participants in some studies, lack of common definitions for cardiac outcomes, and lack of common monitoring either in terms of modality to assess cardiac outcome or in terms of timing and duration of monitoring. This highlights the limitations of CTC reporting from a cardiology perspective.

A previous systematic review of RCTs and cohort studies in patients aged less than 18 years at cancer diagnosis has also addressed anthracycline cardiotoxicity [13]. Only four RCTs were identified, and the same methodological problems were encountered as in our review. Bryant and colleagues found dexrazoxane was noted to offset toxicity, but there was no benefit to longer infusion times in patients receiving moderate doses of anthracyclines.

A large observational study of cardiac complication rates of women aged 66 to 80 years old receiving adjuvant anthracyclines using the Surveillance, Epidemiology and End Results (SEER) database has been conducted [80]. A total of 43,338 women were identified, of whom 4,712 received adjuvant anthracyclines, and 3,012 women non-anthracycline containing regimens. For women aged 66 - 70 years at diagnosis, at 10 years post treatment, 29% of women who had no chemotherapy had been diagnosed with CHF, compared with 32.5% and 38.4% for women who received non-anthracycline and anthracycline based chemotherapy, respectively. The rates were significantly higher for anthracycline regimens compared with non-anthracycline chemotherapy -adjusted hazard ratio (HR) 1.26 (95% CI: 1.12, 1.42). For women aged 71 - 80 years at diagnosis the risk of CHF was not statistically significant between the three groups, although the cumulative rates of CHF were higher than in younger women in all three groups.

The similar rates of CHF in each group may be due to selection bias in the groups treated with adjuvant chemotherapy in this older age group, or reflect a less aggressive approach in those who did receive such treatment. The type of anthracycline used was not reported, but a previous report on a sub-set of the same data suggests that doxorubicin was used almost exclusively [81]. The results from our review suggest that these results cannot be extrapolated to regimens where epirubicin was used, or to younger women as the SEER data include a sub-set of older patients who are at higher risk solely due to their age [82]. However, the data do potentially reflect clinical practice more closely, in contrast with outcomes for the highly selected patients generally included in clinical trials.

Recommendations are that the cumulative dose of anthracycline should not exceed 600 mg/m^2 ^for doxorubicin and 900 mg/m^2 ^for epirubicin. Our data indicate a lower risk with continuous infusions compared with bolus dosing, but in practice this would neither allow dose escalation nor be a practical strategy to minimize cardiotoxicity in 'at risk' groups.

Other significant predictors of anthracycline associated cardiac toxicity include: pre-existing cardiovascular disease such as coronary artery disease, hypertension, peripheral vascular disease, and emphysema, diabetes, ethnicity, age [6,80,83,84]. Treatment related factors are higher cumulative doses of anthracycline, associated mediastinal radiation therapy and combination chemotherapy (trastuzumab, cyclophoshphamide, etoposide, melphalan, paclitaxel, mitoxantrone, idarubicin) [6,17,84]. Longer duration of survival is also a risk factor for cardiac toxicity, emphasising the importance of monitoring for long term effects in the growing population of cancer survivors [84]. This is borne out by paediatric studies indicating continuous deterioration of cardiac function for up to 15 - 30 years after treatment [4,85].

This systemic review and previous published studies have highlighted the potential cardiac sequelae of anthracyclines. As survival and indeed cure rates have increased, increased focus on lifetime risk of cancer and its treatment with strategies to limit short- and long-term toxicity without compromising efficacy is required. This is especially important in the paediatric, adolescent and young adult population where there is a long life expectancy. This requires the clinical assessment and investigation of pre-treatment cardiac risk with monitoring and proactive treatment of the cardiac effects of cancer treatment to become an essential component, not only of clinical trials, where cardiotoxicity may be an important secondary endpoint, but also of routine practice. This will require widespread agreement on cardiac monitoring techniques, and schedules with clear and appropriate cardiac endpoints in order to advance knowledge, and provide optimal cardiology care of oncology patients. Changes are necessary in trial protocols and in clinical practice. In particular:

• The CTC require revision to align them with modern cardiology evidence and practice.

• Cardiac function should be measured, and risk factors for cardiac dysfunction addressed prior to cancer treatment with cardiac toxic medication such as anthracyclines.

• Markers of cardiac damage and repeat measurements of cardiac function should be undertaken at intervals appropriate to the regimen prescribed.

• Cardiac follow-up should be continued long enough to accurately define the risk of long-term toxicity. Primary care colleagues should be alerted to the risks of cardiotoxicity in their patients, and if identified encouraged to inform the oncologist. This should be complimented by regular audit. Only then will it be possible to accurately define the competing life-time risks of cancer and its treatment which is essential to determine the optimal regimen for an individual patient, especially when given in the adjuvant setting.

Currently, cardiac monitoring primarily comprises measurement of LVEF with imaging techniques which have inherent limitations of inter-observer and inter-institution variation and of the late manifestation of ventricular dysfunction in the development of cardiac toxicity. The role of markers of cardiac damage or wall stress, such as cardiac troponins and natriuretic peptides, in predicting late cardiac effects and guiding treatment is being actively investigated in prospective research.

## Conclusions

Unfortunately, published data are not sufficiently robust to support clear evidence-based recommendations on different schedules of anthracyclines, or the routine use of cardiac protective agents or liposomal preparations in patients defined as at high risk for anthracycline cardiotoxicity, such as patients with known cardiac impairment and/or previous anthracycline exposure. Nonetheless, these approaches may have a role. An alternative strategy would be to avoid the use of anthracyclines in favour of newer cytotoxic drugs with equivalent efficacy, but a lower risk of cardiac effects. In breast cancer treatment this might be achieved by moving to taxane-based regimens which appear to have less cardiotoxicity in clinical trials. Currently, most taxane regimens also contain anthracyclines given either concurrently or sequentially, and further RCTs will be required to determine whether anthracyclines can be omitted without compromising efficacy, and that the anticipated reduction in late cardiac and other long-term adverse effects is observed.

Although many risk factors for coronary artery disease are known, and some of these overlap with risk factors for anthracycline induced cardiotoxicity, much less is known about pharmacogenomic risk factors which are important in the pathogenesis of many drug side effects including those of anthracyclines [86]). This is a potentially important area for research. The cardiac effects of cancer treatment usually occur too late to allow dose-adjustment between cycles of chemotherapy, but if patients' individual susceptibilities could be defined in advance of chemotherapy, then prospective modification of regimens could be made not only to reduce anthracycline exposure in high risk individuals, but also possibly to safely allow higher exposure in individuals at low risk of toxicity.

## Competing interests

An unrestricted educational grant was provided to Medical Research Matters by Sanofi Aventis. The funders did not have any role in the design, collection, analysis and interpretation of data, writing the manuscript or approval of the manuscript for publication.

## Authors' contributions

All authors developed the protocol. LS and VC collected the data. VC conducted the analysis and interpreted the results in collaboration with PC, AJ, GL, CP, LS and MV. All authors drafted and critically revised the manuscript and approved the final version.

## Pre-publication history

The pre-publication history for this paper can be accessed here:

http://www.biomedcentral.com/1471-2407/10/337/prepub

## Supplementary Material

Additional file 1 **PRISMA checklist**. Click here for file

Additional file 2 **Medline search strategy**. Click here for file

Additional file 3 **Table S1 Excluded studies**. Click here for file

Additional file 4 **Table S2 Characteristics of included studies**. Click here for file

Additional file 5 **Table S3 Risk factors for cardiotoxicity in included studies**. Click here for file

Additional file 6 **Table S4 Cardiotoxicity outcomes in included studies**. Click here for file

## References

[B1] Sant M Allemani C Santaquilani M Knijn A Marchesi F Capocaccia R EUROCARE Working Group EUROCARE-4. Survival of cancer patients diagnosed in 1995-1999. Results and commentary Eur J Cancer 2009 45 6 931 991 Epub 2009 Jan 2024 10.1016/j.ejca.2008.11.018 19171476

[B2] Mertens AC Liu Q Neglia JP Wasilewski K Leisenring W Armstrong GT Robison LL Yasui Y Cause-specific late mortality among 5-year survivors of childhood cancer: the Childhood Cancer Survivor Study J Natl Cancer Inst 2008 100 1368 1379 10.1093/jnci/djn310 18812549 PMC2556702

[B3] Mulrooney DA Neglia JP Hudson MM Caring for adult survivors of childhood cancer Curr Treat Options Oncol 2008 9 51 66 10.1007/s11864-008-0054-4 18363110

[B4] Mulrooney DA Yeazel MW Kawashima T Mertens AC Mitby P Stovall M Donaldson SS Green DM Sklar CA Robison LL Leisenring WM Cardiac outcomes in a cohort of adult survivors of childhood and adolescent cancer: retrospective analysis of the Childhood Cancer Survivor Study cohort BMJ 2009 339 b4606 10.1136/bmj.b4606 19996459 PMC3266843

[B5] Pai VB Nahata MC Cardiotoxicity of chemotherapeutic agents: incidence, treatment and prevention Drug Saf 2000 22 263 302 10.2165/00002018-200022040-00002 10789823

[B6] Von Hoff DD Layard MW Basa P Davis HL Jr Von Hoff AL Rozencweig M Muggia FM Risk factors for doxorubicin-induced congestive heart failure Ann Intern Med 1979 91 710 717 496103 10.7326/0003-4819-91-5-710

[B7] Ewer MS Lippman SM Type II chemotherapy-related cardiac dysfunction: time to recognize a new entity J Clin Oncol 2005 23 2900 2902 10.1200/JCO.2005.05.827 15860848

[B8] Olivetti G Melissari M Capasso JM Anversa P Cardiomyopathy of the aging human heart. Myocyte loss and reactive cellular hypertrophy Circ Res 1991 68 1560 1568 2036710 10.1161/01.res.68.6.1560

[B9] Yeh ET Tong AT Lenihan DJ Yusuf SW Swafford J Champion C Durand JB Gibbs H Zafarmand AA Ewer MS Cardiovascular complications of cancer therapy: diagnosis, pathogenesis, and management Circulation 2004 109 3122 3131 10.1161/01.CIR.0000133187.74800.B9 15226229

[B10] Murphy CA Dargie HJE Drug-induced cardiovascular disorders Drug Safety 2007 30 783 804 10.2165/00002018-200730090-00005 17722970

[B11] Frei BL Soefje SAE A review of the cardiovascular effects of oncology agents Journal of Pharmacy Practice 2008 21 146 158 10.1177/0897190008314776

[B12] Brandt L Kimby E Nygren P Glimelius B A systematic overview of chemotherapy effects in Hodgkin's disease Acta Oncologica 2001 40 185 197 10.1080/02841860151116240 11441931

[B13] Bryant J Picot J Baxter L Levitt G Sullivan I Clegg A Clinical and cost-effectiveness of cardioprotection against the toxic effects of anthracyclines given to children with cancer: A systematic review British Journal of Cancer 2007 96 226 230 10.1038/sj.bjc.6603562 17242696 PMC2360000

[B14] Kremer LC van der Pal HJ Offringa M van Dalen EC Voute PA Frequency and risk factors of subclinical cardiotoxicity after anthracycline therapy in children: a systematic review (Structured abstract) Annals of Oncology 2002 13 819 829 10.1093/annonc/mdf167 12123328

[B15] Van Dalen E Caron HN Dickinson HO Kremer LCME Cardioprotective interventions for cancer patients receiving anthracyclines Cochrane Database of Systematic Reviews 2008 2 CD003917 10.1002/14651858.CD003917.pub3 18425895

[B16] Van Dalen EC Michiels EMC Caron HN Kremer LCME Different anthracycline derivates for reducing cardiotoxicity in cancer patients Cochrane Database of Systematic Reviews 2010 5 CD005006 10.1002/14651858.CD005006.pub4 PMC6457588 20464735

[B17] Van Dalen EC Van Der Pal HJH Caron HN Kremer LCME Different dosage schedules for reducing cardiotoxicity in cancer patients receiving anthracycline chemotherapy Cochrane Database of Systematic Reviews 2009 4 CD005008 10.1002/14651858.CD005008.pub3 19821337

[B18] Higgins JPT Green S editors Cochrane Handbook for Systematic Reviews of Interventions Version 5.0.2 [updated September 2009] 2009 The Cochrane Collaboration

[B19] Moher D Liberati A Tetzlaff J Altman DG Preferred reporting items for systematic reviews and meta-analyses: the PRISMA statement J Clin Epidemiol 2009 62 1006 1012 10.1016/j.jclinepi.2009.06.005 19631508

[B20] Juni P Altman DG Egger M Systematic reviews in health care: Assessing the quality of controlled clinical trials BMJ 2001 323 42 46 10.1136/bmj.323.7303.42 11440947 PMC1120670

[B21] Bradburn MJ Deeks JJ Berlin JA Russell Localio A Much ado about nothing: a comparison of the performance of meta-analytical methods with rare events Stat Med 2007 26 53 77 10.1002/sim.2528 16596572

[B22] DerSimonian R Laird N Meta-analysis in clinical trials Control Clin Trials 1986 7 177 188 10.1016/0197-2456(86)90046-2 3802833

[B23] Higgins JP Thompson SG Deeks JJ Altman DG Measuring inconsistency in meta-analyses BMJ 2003 327 557 560 10.1136/bmj.327.7414.557 12958120 PMC192859

[B24] Ackland SP Anton A Breitbach GP Colajori E Tursi JM Delfino C Efremidis A Ezzat A Fittipaldo A Kolaric K Lopez M Viaro D Dose-intensive epirubicin-based chemotherapy is superior to an intensive intravenous cyclophosphamide, methotrexate, and fluorouracil regimen in metastatic breast cancer: a randomized multinational study Journal of Clinical Oncology 2001 19 943 953 11181656 10.1200/JCO.2001.19.4.943

[B25] Feher O Vodvarka P Jassem J Morack G Advani SH Khoo KS Doval DC Ermisch S Roychowdhury D Miller MA von Minckwitz G First-line gemcitabine versus epirubicin in postmenopausal women aged 60 or older with metastatic breast cancer: A multicenter, randomized, phase III study Annals of Oncology 2005 16 899 908 10.1093/annonc/mdi181 15821120

[B26] Levine MN Pritchard KI Bramwell VH Shepherd LE Tu D Paul N National Cancer Institute of Canada Clinical Trials G Randomized trial comparing cyclophosphamide, epirubicin, and fluorouracil with cyclophosphamide, methotrexate, and fluorouracil in premenopausal women with node-positive breast cancer: update of National Cancer Institute of Canada Clinical Trials Group Trial MA5 Journal of Clinical Oncology 2005 23 5166 5170 10.1200/JCO.2005.09.423 16051958

[B27] Martin M Villar A Sole-Calvo A Gonzalez R Massuti B Lizon J Camps C Carrato A Casado A Candel MT Albanell J Aranda J Munarriz B Campbell J Diaz-Rubio E Geicam Group S Doxorubicin in combination with fluorouracil and cyclophosphamide (i.v. FAC regimen, day 1, 21) versus methotrexate in combination with fluorouracil and cyclophosphamide (i.v. CMF regimen, day 1, 21) as adjuvant chemotherapy for operable breast cancer: a study by the GEICAM group Annals of Oncology 2003 14 833 842 10.1093/annonc/mdg260 12796019

[B28] Hernadi Z Juhasz B Poka R Lampe LG Randomised trial comparing combinations of cyclophosphamide and cisplatin without or with doxorubicin or 4'-epi-doxorubicin in the treatment of advanced ovarian cancer International Journal of Gynaecology & Obstetrics 1988 27 199 204 10.1016/0020-7292(88)90008-2 2903085

[B29] Sposto R Meadows AT Chilcote RR Steinherz PG Kjeldsberg C Kadin ME Krailo MD Termuhlen AM Morse M Siegel SE Comparison of long-term outcome of children and adolescents with disseminated non-lymphoblastic non-Hodgkin lymphoma treated with COMP or daunomycin-COMP: A report from the Children's Cancer Group Medical & Pediatric Oncology 2001 37 432 441 10.1002/mpo.1226 11745871

[B30] Sullivan MP Fuller LM Berard C Ternberg J Cantor AB Leventhal BG Comparative effectiveness of two combined modality regimens in the treatment of surgical stage III Hodgkin's disease in children. An 8-year follow-up study by the Pediatric Oncology Group American Journal of Pediatric Hematology/Oncology 1991 13 450 458 10.1097/00043426-199124000-00010 1785672

[B31] Barnes R Sweetnam DR Bleehen NM A trial of chemotherapy in patients with osteosarcoma. (A report to the Medical Research Council by the working party on bone sarcoma Br J Cancer 1986 53 513 518 2871860 10.1038/bjc.1986.81 PMC2001430

[B32] Alonso MC Tabernero JM Ojeda B Llanos M Sola C Climent MA Segui MA Lopez JJ A phase III randomized trial of cyclophosphamide, mitoxantrone, and 5-fluorouracil (CNF) versus cyclophosphamide, adriamycin, and 5-fluorouracil (CAF) in patients with metastatic breast cancer Breast Cancer Research & Treatment 1995 34 15 24 10.1007/BF00666487 7749156

[B33] Bennett JM Muss HB Doroshow JH Wolff S Krementz ET Cartwright K Dukart G Reisman A Schoch I A randomized multicenter trial comparing mitoxantrone, cyclophosphamide, and fluorouracil with doxorubicin, cyclophosphamide, and fluorouracil in the therapy of metastatic breast carcinoma Journal of Clinical Oncology 1988 6 1611 1620 3049953 10.1200/JCO.1988.6.10.1611

[B34] Cook AM Chambers EJ Rees GJ Comparison of mitozantrone and epirubicin in advanced breast cancer Clinical Oncology 1996 8 363 366 10.1016/S0936-6555(96)80079-3 8973851

[B35] Estaban E Lacave AJ Fernandez JL Corral N Buesa JM Estrada E Palacio I Vieitez JM Muniz I Alvarez E Phase III trial of cyclophosphamide, epirubicin, fluorouracil (CEF) versus cyclophosphamide, mitoxantrone, fluorouracil (CNF) in women with metastatic breast cancer Breast Cancer Research & Treatment 1999 58 141 150 10.1023/A:1006387801960 10674879

[B36] Follezou JY Palangie T Feuilhade F Randomized trial comparing mitoxantrone with adriamycin in advanced breast cancer Presse Medicale 1987 16 765 768 2954077

[B37] Hausmaninger H Lehnert M Steger G Sevelda P Tschurtschenthaler G Hehenwarter W Fridrik M Samonigg H Schiller L Manfreda D Randomised phase II study of epirubicin-vindesine versus mitoxantrone-vindesine in metastatic breast cancer European Journal of Cancer 31A 1995 10.1016/0959-8049(95)00489-0 8652237

[B38] Heidemann E Steinke B Hartlapp J Schumacher K Possinger K Kunz S Neeser EVIG Hossfeld D Caffier H Souchon R Waldmann R Blumner E Clark J Prognostic subgroups: The key factor for treatment outcome in metastatic breast cancer. Results of three-arm randomized multicenter trial comparing doxorubicin, epirubicin and mitoxantrone each in combination with cyclophosphamide Onkologie 1993 16 344 353 10.1159/000218287

[B39] Henderson IC Allegra JC Woodcock T Wolff S Bryan S Cartwright K Dukart G Henry D Randomized clinical trial comparing mitoxantrone with doxorubicin in previously treated patients with metastatic breast cancer Journal of Clinical Oncology 1989 7 560 571 2468745 10.1200/JCO.1989.7.5.560

[B40] Lawton PA Spittle MF Ostrowski MJ Young T Madden F Folkes A Hill BT MacRae K A comparison of doxorubicin, epirubicin and mitozantrone as single agents in advanced breast carcinoma Clinical Oncology 1993 5 80 84 10.1016/S0936-6555(05)80851-9 8481365

[B41] Periti P Pannuti F Della Cuna GR Mazzei T Mini E Martoni A Preti P Ercolino L Pavesi L Ribecco A Combination chemotherapy with cyclophosphamide, fluorouracil, and either epirubicin or mitoxantrone: a comparative randomized multicenter study in metastatic breast carcinoma Cancer Investigation 1991 9 249 255 10.3109/07357909109021321 1913227

[B42] Stewart DJ Evans WK Shepherd FA Wilson KS Pritchard KI Trudeau ME Wilson JJ Martz KE Cyclophosphamide and fluorouracil combined with mitoxantrone versus doxorubicin for breast cancer: Superiority of doxorubicin Journal of Clinical Oncology 1997 15 1897 1905 9164200 10.1200/JCO.1997.15.5.1897

[B43] Cavo M Benni M Ronconi S Fiacchini M Gozzetti A Zamagni E Cellini C Tosi P Baccarani M Tura S Melphalan-prednisone versus alternating combination VAD/MP or VND/MP as primary therapy for multiple myeloma: Final analysis of a randomized clinical study Haematologica 2002 87 934 942 12217805

[B44] Gherlinzoni F Guglielmi C Mazza P Amadori S Mandelli F Tura S Phase III comparative trial (m-BACOD v m-BNCOD) in the treatment of stage II to IV non-Hodgkin's lymphomas with intermediate- or high-grade histology Seminars in Oncology 1990 17 3 8 1701924

[B45] Aviles A Guzman R Talavera A Garcia EL Diaz-Maqueo JC Randomized study for the treatment of adult advanced Hodgkin's disease: epirubicin, vinblastine, bleomycin, and dacarbazine (EVBD) versus mitoxantrone, vinblastine, bleomycin, and dacarbazine (MVBD) Medical & Pediatric Oncology 1994 22 168 172 10.1002/mpo.2950220304 7505877

[B46] Pavlovsky S Santarelli MT Erazo A Diaz Maqueo JC Somoza N Lluesma Gonalons M Cervantes G Garcia Vela EL Corrado C Magnasco H Results of a randomized study of previously-untreated intermediate and high grade lymphoma using CHOP versus CNOP.[see comment] Annals of Oncology 1992 3 205 209 1586618 10.1093/oxfordjournals.annonc.a058153

[B47] Hortobagyi GN Yap HY Kau SW Fraschini G Ewer MS Chawla SP Benjamin RS A comparative study of doxorubicin and epirubicin in patients with metastatic breast cancer American Journal of Clinical Oncology 1989 12 57 62 10.1097/00000421-198902000-00014 2643296

[B48] Shapira J Gotfried M Lishner M Ravid M Reduced cardiotoxicity of doxorubicin by a 6-hour infusion regimen. A prospective randomized evaluation Cancer 1990 65 870 873 10.1002/1097-0142(19900215)65:4<870::AID-CNCR2820650407>3.0.CO;2-D 2297656

[B49] Casper ES Gaynor JJ Hajdu SI Magill GB Tan C Friedrich C Brennan MF A prospective randomized trial of adjuvant chemotherapy with bolus versus continuous infusion of doxorubicin in patients with high-grade extremity soft tissue sarcoma and an analysis of prognostic factors Cancer 1991 68 1221 1229 10.1002/1097-0142(19910915)68:6<1221::AID-CNCR2820680607>3.0.CO;2-R 1873773

[B50] Zalupski M Metch B Balcerzak S Fletcher WS Chapman R Bonnet JD Weiss GR Ryan J Benjamin RS Baker LH Phase III comparison of doxorubicin and dacarbazine given by bolus versus infusion in patients with soft-tissue sarcomas: a Southwest Oncology Group study Journal of the National Cancer Institute 1991 83 926 932 10.1093/jnci/83.13.926 2067035

[B51] French Epirubicin Study Group A prospective randomized phase III trial comparing combination chemotherapy with cyclophosphamide, fluorouracil, and either doxorubicin or epirubicin. French Epirubicin Study Group J Clin Oncol 1988 6 679 688 2895801 10.1200/JCO.1988.6.4.679

[B52] Bontenbal M Andersson M Wildiers J Cocconi G Jassem J Paridaens R Rotmensz N Sylvester R Mouridsen HT Klijn JG van Oosterom AT Doxorubicin vs epirubicin, report of a second-line randomized phase II/III study in advanced breast cancer. EORTC Breast Cancer Cooperative Group British Journal of Cancer 1998 77 2257 2263 9649142 10.1038/bjc.1998.375 PMC2150384

[B53] Brambilla C Rossi A Bonfante V Ferrari L Villani F Crippa F Bonadonna G Phase II study of doxorubicin versus epirubicin in advanced breast cancer Cancer Treatment Reports 1986 70 261 266 3456271

[B54] Gasparini G Dal Fior S Panizzoni GA Favretto S Pozza F Weekly epirubicin versus doxorubicin as second line therapy in advanced breast cancer. A randomized clinical trial American Journal of Clinical Oncology 1991 14 38 44 10.1097/00000421-199102000-00009 1987737

[B55] Jain KK Casper ES Geller NL Hakes TB Kaufman RJ Currie V Schwartz W Cassidy C Petroni GR Young CW A prospective randomized comparison of epirubicin and doxorubicin in patients with advanced breast cancer Journal of Clinical Oncology 1985 3 818 826 3859587 10.1200/JCO.1985.3.6.818

[B56] Perez DJ Harvey VJ Robinson BA Atkinson CH Dady PJ Kirk AR Evans BD Chapman PJ A randomized comparison of single-agent doxorubicin and epirubicin as first-line cytotoxic therapy in advanced breast cancer Journal of Clinical Oncology 1991 9 2148 2152 1960557 10.1200/JCO.1991.9.12.2148

[B57] Phase III randomized study of fluorouracil, epirubicin, and cyclophosphamide v fluorouracil, doxorubicin, and cyclophosphamide in advanced breast cancer: an Italian multicentre trial. Italian Multicentre Breast Study with Epirubicin J Clin Oncol 1988 6 976 982 2897433 10.1200/JCO.1988.6.6.976

[B58] Bezwoda WR Treatment of advanced ovarian cancer: a randomised trial comparing adriamycin or 4'epi-adriamycin in combination with cisplatin and cyclophosphamide Medical & Pediatric Oncology 1986 14 26 29 10.1002/mpo.2950140107 3512971

[B59] Homesley HD Harry DS O'Toole RV Hoogstraten B Franklin EW Cavanagh D Nahhas WA Smith JJ Lovelace JV Randomized comparison of cisplatin plus epirubicin or doxorubicin for advanced epithelial ovarian carcinoma. A multicenter trial American Journal of Clinical Oncology 1992 15 129 134 10.1097/00000421-199204000-00007 1553900

[B60] Lahtinen R Kuikka J Nousiainen T Uusitupa M Lansimies E Cardiotoxicity of epirubicin and doxorubicin: a double-blind randomized study European Journal of Haematology 1991 46 301 305 2044726 10.1111/j.1600-0609.1991.tb01543.x

[B61] Batist G Ramakrishnan G Rao CS Chandrasekharan A Gutheil J Guthrie T Shah P Khojasteh A Nair MK Hoelzer K Tkaczuk K Park YC Lee LW Reduced cardiotoxicity and preserved antitumor efficacy of liposome-encapsulated doxorubicin and cyclophosphamide compared with conventional doxorubicin and cyclophosphamide in a randomized, multicenter trial of metastatic breast cancer.[see comment] Journal of Clinical Oncology 2001 19 1444 1454 11230490 10.1200/JCO.2001.19.5.1444

[B62] Harris L Batist G Belt R Rovira D Navari R Azarnia N Welles L Winer E Groupmedline TDS Liposome-encapsulated doxorubicin compared with conventional doxorubicin in a randomized multicenter trial as first-line therapy of metastatic breast carcinoma Cancer 2002 94 25 36 10.1002/cncr.10201 11815957

[B63] O'Brien ME Wigler N Inbar M Rosso R Grischke E Santoro A Catane R Kieback DG Tomczak P Ackland SP Orlandi F Mellars L Alland L Tendler C Groupmedline CBCS Reduced cardiotoxicity and comparable efficacy in a phase III trial of pegylated liposomal doxorubicin HCl (CAELYX/Doxil) versus conventional doxorubicin for first-line treatment of metastatic breast cancer Annals of Oncology 2004 15 440 449 10.1093/annonc/mdh097 14998846

[B64] Rifkin RM Gregory SA Mohrbacher A Hussein MA Pegylated liposomal doxorubicin, vincristine, and dexamethasone provide significant reduction in toxicity compared with doxorubicin, vincristine, and dexamethasone in patients with newly diagnosed multiple myeloma: A phase III multicenter randomized trial Cancer 2006 106 848 858 10.1002/cncr.21662 16404741

[B65] Chan S Davidson N Juozaityte E Erdkamp F Pluzanska A Azarnia N Lee LW Phase III trial of liposomal doxorubicin and cyclophosphamide compared with epirubicin and cyclophosphamide as first-line therapy for metastatic breast cancer Annals of Oncology 2004 15 1527 1534 10.1093/annonc/mdh393 15367414

[B66] Zinzani PL Martelli M Storti S Musso M Cantonetti M Leone G Cajozzo A Papa G Iannitto E Perrotti A Phase III comparative trial using CHOP vs CIOP in the treatment of advanced intermediate-grade non-Hodgkin's lymphoma Leukemia & Lymphoma 1995 19 329 335 10.3109/10428199509107906 8535227

[B67] Federico M Clo V Brugiatelli M Carotenuto M Gobbi PG Vallisa D Lombardo M Avanzini P Di Renzo N Dini D Baldini L Silingardi V Efficacy of two different ProMACE-CytaBOM derived regimens in advanced aggressive non-Hodgkin's lymphoma. Final report of a multicenter trial conducted by GISL Haematologica 1998 83 800 811 9825577

[B68] Marty M Espie M Llombart A Monnier A Rapoport BL Stahalova V Dexrazoxane Study G Multicenter randomized phase III study of the cardioprotective effect of dexrazoxane (Cardioxane) in advanced/metastatic breast cancer patients treated with anthracycline-based chemotherapy Annals of Oncology 2006 17 614 622 10.1093/annonc/mdj134 16423847

[B69] Speyer JL Green MD Zeleniuch-Jacquotte A Wernz JC Rey M Sanger J Kramer E Ferrans V Hochster H Meyers M ICRF-187 permits longer treatment with doxorubicin in women with breast cancer.[erratum appears in J Clin Oncol 1992 May;10(5):867] Journal of Clinical Oncology 1992 10 117 127 1727913 10.1200/JCO.1992.10.1.117

[B70] Swain SM Whaley FS Gerber MC Weisberg S York M Spicer D Jones SE Wadler S Desai A Vogel C Speyer J Mittelman A Reddy S Pendergrass K Velez-Garcia E Ewer M Bianchine J Gams R Cardioprotection with dexrazoxane for doxorubicin-containing therapy in advanced breast cancer Journal of Clinical Oncology 1997 15 1318 1332 9193323 10.1200/JCO.1997.15.4.1318

[B71] Venturini M Michelotti A Del Mastro L Gallo L Carnino F Garrone O Tibaldi C Molea N Bellina RC Pronzato P Cyrus P Vinke J Testore F Guelfi M Lionetto R Bruzzi P Conte PF Rosso Rm Multicenter randomized controlled clinical trial to evaluate cardioprotection of dexrazoxane versus no cardioprotection in women receiving epirubicin chemotherapy for advanced breast cancer Journal of Clinical Oncology 1996 14 3112 3120 8955656 10.1200/JCO.1996.14.12.3112

[B72] Wexler LH Andrich MP Venzon D Berg SL Weaver-McClure L Chen CC Dilsizian V Avila N Jarosinski P Balis FM Poplack DG Horowitz ME Randomized trial of the cardioprotective agent ICRF-187 in pediatric sarcoma patients treated with doxorubicin Journal of Clinical Oncology 1996 14 362 372 8636745 10.1200/JCO.1996.14.2.362

[B73] Lopez M Vici P Di Lauro K Conti F Paoletti G Ferraironi A Sciuto R Giannarelli D Maini CL Randomized prospective clinical trial of high-dose epirubicin and dexrazoxane in patients with advanced breast cancer and soft tissue sarcomas Journal of Clinical Oncology 1998 16 86 92 9440727 10.1200/JCO.1998.16.1.86

[B74] Kalay N Basar E Ozdogru I Er O Cetinkaya Y Dogan A Inanc T Oguzhan A Eryol NK Topsakal R Ergin AE Protective Effects of Carvedilol Against Anthracycline-Induced Cardiomyopathy Journal of the American College of Cardiology 2006 48 2258 2262 10.1016/j.jacc.2006.07.052 17161256

[B75] Waldner R Laschan C Lohninger A Gessner M Tuchler H Huemer M Spiegel W Karlic H Effects of doxorubicin-containing chemotherapy and a combination with L-carnitine on oxidative metabolism in patients with non-Hodgkin lymphoma Journal of Cancer Research & Clinical Oncology 2006 132 121 128 10.1007/s00432-005-0054-8 PMC12161048 16283381

[B76] Milei J Marantz A Ale J Vazquez A Buceta JE Prevention of adriamycin-induced cardiotoxicity by prenylamine: a pilot double blind study Cancer Drug Deliv 1987 4 129 136 3427552 10.1089/cdd.1987.4.129

[B77] Gallegos-Castorena S Martinez-Avalos A Mohar-Betancourt A Guerrero-Avendano G Zapata-Tarres M Medina-Sanson A Toxicity prevention with amifostine in pediatric osteosarcoma patients treated with cisplatin and doxorubicin Pediatric Hematology & Oncology 2007 24 403 408 10.1080/08880010701451244 17710657

[B78] Myers C Bonow R Palmeri S Jenkins J Corden B Locker G Doroshow J Epstein S A randomized controlled trial assessing the prevention of doxorubicin cardiomyopathy by N-acetylcysteine Semin Oncol 1983 10 53 55 6340204

[B79] Cvetkovic RS Scott LJ Dexrazoxane: a review of its use for cardioprotection during anthracycline chemotherapy Drugs 2005 65 1005 1024 10.2165/00003495-200565070-00008 15892593

[B80] Pinder MC Duan Z Goodwin JS Hortobagyi GN Giordano SH Congestive heart failure in older women treated with adjuvant anthracycline chemotherapy for breast cancer J Clin Oncol 2007 25 3808 3815 10.1200/JCO.2006.10.4976 17664460

[B81] Doyle JJ Neugut AI Jacobson JS Grann VR Hershman DL Chemotherapy and cardiotoxicity in older breast cancer patients: a population-based study J Clin Oncol 2005 23 8597 8605 10.1200/JCO.2005.02.5841 16314622

[B82] Bristow MR Billingham ME Mason JW Daniels JR Clinical spectrum of anthracycline antibiotic cardiotoxicity Cancer Treat Rep 1978 62 873 879 667861

[B83] Hershman DL McBride RB Eisenberger A Tsai WY Grann VR Jacobson JS Doxorubicin, cardiac risk factors, and cardiac toxicity in elderly patients with diffuse B-cell non-Hodgkin's lymphoma J Clin Oncol 2008 26 3159 3165 10.1200/JCO.2007.14.1242 18591554

[B84] Carver JR Shapiro CL Ng A Jacobs L Schwartz C Virgo KS Hagerty KL Somerfield MR Vaughn DJ American Society of Clinical Oncology clinical evidence review on the ongoing care of adult cancer survivors: cardiac and pulmonary late effects J Clin Oncol 2007 25 3991 4008 10.1200/JCO.2007.10.9777 17577017

[B85] Pein F Sakiroglu O Dahan M Lebidois J Merlet P Shamsaldin A Villain E De Vathaire F Sidi D Hartmann O Cardiac abnormalities 15 years and more after adriamycin therapy in 229 childhood survivors of a solid tumour at the Institut Gustave Roussy British Journal of Cancer 2004 91 37 44 10.1038/sj.bjc.6601904 15162142 PMC2364747

[B86] Wojnowski L Kulle B Schirmer M Schluter G Schmidt A Rosenberger A Vonhof S Bickeboller H Toliat MR Suk EK Tzvetkov M Kruger A Seifert S Kloess M Hahn H Loeffler M Nurnberg P Pfreundschuh M Trumper L Brockmoller J Hasenfuss G NAD(P)H oxidase and multidrug resistance protein genetic polymorphisms are associated with doxorubicin-induced cardiotoxicity Circulation 2005 112 3754 3762 10.1161/CIRCULATIONAHA.105.576850 16330681

