# Supplementary material for: Cardiotoxicity of anthracycline agents for the treatment of cancer: Systematic review and meta-analysis of randomised controlled trials
Source: BMC Cancer. 2010 Jun 29;10:337. doi: 10.1186/1471-2407-10-337 (PMC2907344; doi:10.1186/1471-2407-10-337)
Supplement: Additional file 1 — PRISMA checklist. [file 1471-2407-10-337-S1.DOC]

Additional file 2: Medline search

Ovid MEDLINE(R) 1950 to June Week 4 2008

| # | Searches | Results |
| --- | --- | --- |
| 1 | exp Anthracyclines/ | 41792 |
| 2 | anthracycline*.mp. [mp=title, original title, abstract, name of substance word, subject heading word] | 8043 |
| 3 | exp Idarubicin/ | 1153 |
| 4 | 4-demethoxydaunorubicin.mp. [mp=title, original title, abstract, name of substance word, subject heading word] | 141 |
| 5 | 4 demethoxydaunorubicin.mp. [mp=title, original title, abstract, name of substance word, subject heading word] | 141 |
| 6 | IMI 30.mp. [mp=title, original title, abstract, name of substance word, subject heading word] | 9 |
| 7 | IMI30.mp. [mp=title, original title, abstract, name of substance word, subject heading word] | 1 |
| 8 | IMI-30.mp. [mp=title, original title, abstract, name of substance word, subject heading word] | 9 |
| 9 | idarubicin hydrochloride.mp. [mp=title, original title, abstract, name of substance word, subject heading word] | 15 |
| 10 | hydrochloride, idarubicin.mp. [mp=title, original title, abstract, name of substance word, subject heading word] | 0 |
| 11 | NSC 256439.mp. [mp=title, original title, abstract, name of substance word, subject heading word] | 4 |
| 12 | NSC-256439.mp. [mp=title, original title, abstract, name of substance word, subject heading word] | 4 |
| 13 | NSC256439.mp. [mp=title, original title, abstract, name of substance word, subject heading word] | 0 |
| 14 | idarubicin.mp. [mp=title, original title, abstract, name of substance word, subject heading word] | 1433 |
| 15 | idarubic*.mp. [mp=title, original title, abstract, name of substance word, subject heading word] | 1452 |
| 16 | 4 desmethoxydaunorubicin.mp. [mp=title, original title, abstract, name of substance word, subject heading word] | 0 |
| 17 | 4-desmethoxydaunorubicin.mp. [mp=title, original title, abstract, name of substance word, subject heading word] | 0 |
| 18 | (pegylated or pegyl* or encapsulated or encapsul* or liposomal or liposom*).mp. [mp=title, original title, abstract, name of substance word, subject heading word] | 52982 |
| 19 | exp Epirubicin/ | 3399 |
| 20 | 4'-epiadriamycin.mp. [mp=title, original title, abstract, name of substance word, subject heading word] | 33 |
| 21 | 4' epiadriamycin.mp. [mp=title, original title, abstract, name of substance word, subject heading word] | 33 |
| 22 | 4'-epidoxorubicin.mp. [mp=title, original title, abstract, name of substance word, subject heading word] | 149 |
| 23 | 4' epidoxorubicin.mp. [mp=title, original title, abstract, name of substance word, subject heading word] | 149 |
| 24 | 4'-epi-doxorubicin.mp. [mp=title, original title, abstract, name of substance word, subject heading word] | 93 |
| 25 | 4' epi doxorubicin.mp. [mp=title, original title, abstract, name of substance word, subject heading word] | 93 |
| 26 | 4'-epi-adriamycin.mp. [mp=title, original title, abstract, name of substance word, subject heading word] | 49 |
| 27 | 4' epi adriamycin.mp. [mp=title, original title, abstract, name of substance word, subject heading word] | 49 |
| 28 | 4'-epi-DXR.mp. [mp=title, original title, abstract, name of substance word, subject heading word] | 0 |
| 29 | 4' epi DXR.mp. [mp=title, original title, abstract, name of substance word, subject heading word] | 0 |
| 30 | epirubicin hydrochloride.mp. [mp=title, original title, abstract, name of substance word, subject heading word] | 31 |
| 31 | hydrochloride,epirubicin.mp. [mp=title, original title, abstract, name of substance word, subject heading word] | 0 |
| 32 | farmorubicin.mp. [mp=title, original title, abstract, name of substance word, subject heading word] | 65 |
| 33 | IMI-28.mp. [mp=title, original title, abstract, name of substance word, subject heading word] | 0 |
| 34 | IMI 28.mp. [mp=title, original title, abstract, name of substance word, subject heading word] | 0 |
| 35 | IMI28.mp. [mp=title, original title, abstract, name of substance word, subject heading word] | 1 |
| 36 | NSC256942.mp. [mp=title, original title, abstract, name of substance word, subject heading word] | 0 |
| 37 | NSC-256942.mp. [mp=title, original title, abstract, name of substance word, subject heading word] | 1 |
| 38 | NSC256942.mp. [mp=title, original title, abstract, name of substance word, subject heading word] | 0 |
| 39 | epirubic*.mp. [mp=title, original title, abstract, name of substance word, subject heading word] | 4235 |
| 40 | exp Doxorubicin/ | 33277 |
| 41 | adriablastine.mp. [mp=title, original title, abstract, name of substance word, subject heading word] | 14 |
| 42 | adriblastin.mp. [mp=title, original title, abstract, name of substance word, subject heading word] | 60 |
| 43 | adriablastin.mp. [mp=title, original title, abstract, name of substance word, subject heading word] | 42 |
| 44 | adriamycin.mp. [mp=title, original title, abstract, name of substance word, subject heading word] | 12527 |
| 45 | DOX-SL.mp. [mp=title, original title, abstract, name of substance word, subject heading word] | 9 |
| 46 | DOX SL.mp. [mp=title, original title, abstract, name of substance word, subject heading word] | 9 |
| 47 | DOXSL.mp. [mp=title, original title, abstract, name of substance word, subject heading word] | 0 |
| 48 | doxorubicin hydrochloride.mp. [mp=title, original title, abstract, name of substance word, subject heading word] | 398 |
| 49 | hydrochloride,doxorubicin.mp. [mp=title, original title, abstract, name of substance word, subject heading word] | 6 |
| 50 | doxorubic*.mp. [mp=title, original title, abstract, name of substance word, subject heading word] | 36351 |
| 51 | adriamyc*.mp. [mp=title, original title, abstract, name of substance word, subject heading word] | 12716 |
| 52 | doxil.mp. [mp=title, original title, abstract, name of substance word, subject heading word] | 194 |
| 53 | caelyx.mp. [mp=title, original title, abstract, name of substance word, subject heading word] | 128 |
| 54 | liposomal doxorubicin.mp. [mp=title, original title, abstract, name of substance word, subject heading word] | 725 |
| 55 | doxrubicin,liposomal.mp. [mp=title, original title, abstract, name of substance word, subject heading word] | 0 |
| 56 | daunorubicin.mp. [mp=title, original title, abstract, name of substance word, subject heading word] | 7838 |
| 57 | exp Daunorubicin/ | 39444 |
| 58 | dauno-rubidomycine.mp. [mp=title, original title, abstract, name of substance word, subject heading word] | 0 |
| 59 | dauno rubidomycin.mp. [mp=title, original title, abstract, name of substance word, subject heading word] | 2 |
| 60 | dauno-rubidomycin.mp. [mp=title, original title, abstract, name of substance word, subject heading word] | 2 |
| 61 | rubidomycin.mp. [mp=title, original title, abstract, name of substance word, subject heading word] | 110 |
| 62 | rubomycin.mp. [mp=title, original title, abstract, name of substance word, subject heading word] | 240 |
| 63 | daunomycin.mp. [mp=title, original title, abstract, name of substance word, subject heading word] | 1698 |
| 64 | cerubidine.mp. [mp=title, original title, abstract, name of substance word, subject heading word] | 5 |
| 65 | daunoblastine.mp. [mp=title, original title, abstract, name of substance word, subject heading word] | 8 |
| 66 | daunorubicin hydrochloride.mp. [mp=title, original title, abstract, name of substance word, subject heading word] | 20 |
| 67 | hydrochloride, daunorubicin.mp. [mp=title, original title, abstract, name of substance word, subject heading word] | 2 |
| 68 | daunorubic*.mp. [mp=title, original title, abstract, name of substance word, subject heading word] | 7862 |
| 69 | rubidomyc*.mp. [mp=title, original title, abstract, name of substance word, subject heading word] | 113 |
| 70 | NSC-82151.mp. [mp=title, original title, abstract, name of substance word, subject heading word] | 52 |
| 71 | NSC 82151.mp. [mp=title, original title, abstract, name of substance word, subject heading word] | 52 |
| 72 | NSC82151.mp. [mp=title, original title, abstract, name of substance word, subject heading word] | 0 |
| 73 | daunoxome.mp. [mp=title, original title, abstract, name of substance word, subject heading word] | 86 |
| 74 | daunosom*.mp. [mp=title, original title, abstract, name of substance word, subject heading word] | 1 |
| 75 | 1 or 2 or 3 or 4 or 5 or 6 or 7 or 8 or 9 or 10 or 11 or 12 or 13 or 14 or 15 or 16 or 17 | 44970 |
| 76 | 18 and 75 | 1757 |
| 77 | 19 or 20 or 21 or 22 or 23 or 24 or 25 or 26 or 27 or 28 or 29 or 30 or 31 or 32 or 33 or 34 or 35 or 36 or 37 or 38 or 39 | 4335 |
| 78 | 18 and 77 | 58 |
| 79 | 40 or 41 or 42 or 43 or 44 or 45 or 46 or 47 or 48 or 49 or 50 or 51 | 41969 |
| 80 | 18 or 52 or 53 or 54 or 55 | 53003 |
| 81 | 79 and 80 | 1728 |
| 82 | 57 or 58 or 59 or 60 or 61 or 62 or 63 or 64 or 65 or 66 or 67 or 68 or 69 or 70 or 71 or 72 | 40974 |
| 83 | 18 or 73 or 74 | 52987 |
| 84 | 82 and 83 | 1682 |
| 85 | 75 or 76 or 77 or 78 or 79 or 81 or 82 or 84 | 53954 |
| 86 | exp Breast Cancer/ | 159104 |
| 87 | exp Ovary Cancer/ | 49163 |
| 88 | exp CLASSICAL HODGKIN LYMPHOMA/ or exp NONHODGKIN LYMPHOMA/ or exp LYMPHOMA/ | 122951 |
| 89 | exp SARCOMA/ | 97389 |
| 90 | exp multiple myeloma/ or exp myeloma/ | 24725 |
| 91 | lymphoma*.mp. [mp=title, original title, abstract, name of substance word, subject heading word] | 142944 |
| 92 | non-hodgkin*.mp. [mp=title, original title, abstract, name of substance word, subject heading word] | 38813 |
| 93 | hodgkin*.mp. [mp=title, original title, abstract, name of substance word, subject heading word] | 65591 |
| 94 | myeloma*.mp. [mp=title, original title, abstract, name of substance word, subject heading word] | 35632 |
| 95 | waldenstrom*.mp. [mp=title, original title, abstract, name of substance word, subject heading word] | 4762 |
| 96 | myelomatosis.mp. [mp=title, original title, abstract, name of substance word, subject heading word] | 671 |
| 97 | sarcoma*.mp. [mp=title, original title, abstract, name of substance word, subject heading word] | 79941 |
| 98 | (breast adj1 cancer).mp. [mp=title, original title, abstract, name of substance word, subject heading word] | 105669 |
| 99 | (breast adj1 carcinoma).mp. [mp=title, original title, abstract, name of substance word, subject heading word] | 15684 |
| 100 | (ovarian adj1 cancer).mp. [mp=title, original title, abstract, name of substance word, subject heading word] | 20767 |
| 101 | (ovarian adj1 carcinoma).mp. [mp=title, original title, abstract, name of substance word, subject heading word] | 8097 |
| 102 | (ovary adj1 cancer).mp. [mp=title, original title, abstract, name of substance word, subject heading word] | 136 |
| 103 | (ovary adj1 carcinoma).mp. [mp=title, original title, abstract, name of substance word, subject heading word] | 93 |
| 104 | (gynaecologic* adj1 cancer).mp. [mp=title, original title, abstract, name of substance word, subject heading word] | 445 |
| 105 | (gynaecologic* adj1 carcinoma).mp. [mp=title, original title, abstract, name of substance word, subject heading word] | 23 |
| 106 | (gynecologic* adj1 cancer).mp. [mp=title, original title, abstract, name of substance word, subject heading word] | 1596 |
| 107 | (gynecologic* adj1 carcinoma).mp. [mp=title, original title, abstract, name of substance word, subject heading word] | 100 |
| 108 | Randomized Controlled Trial/ | 261258 |
| 109 | 86 or 87 or 88 or 89 or 90 or 91 or 92 or 93 or 94 or 95 or 96 or 97 or 98 or 99 or 100 or 101 or 102 or 103 or 104 or 105 or 106 or 107 | 521736 |
| 110 | 85 and 109 | 21374 |
| 111 | 108 and 110 | 1973 |
| 112 | limit 111 to humans | 1955 |
