# Supplementary material for: Cardiotoxicity of anthracycline agents for the treatment of cancer: Systematic review and meta-analysis of randomised controlled trials
Source: BMC Cancer. 2010 Jun 29;10:337. doi: 10.1186/1471-2407-10-337 (PMC2907344; doi:10.1186/1471-2407-10-337)
Supplement: Additional file 2 — Medline search strategy. [file 1471-2407-10-337-S2.DOC]

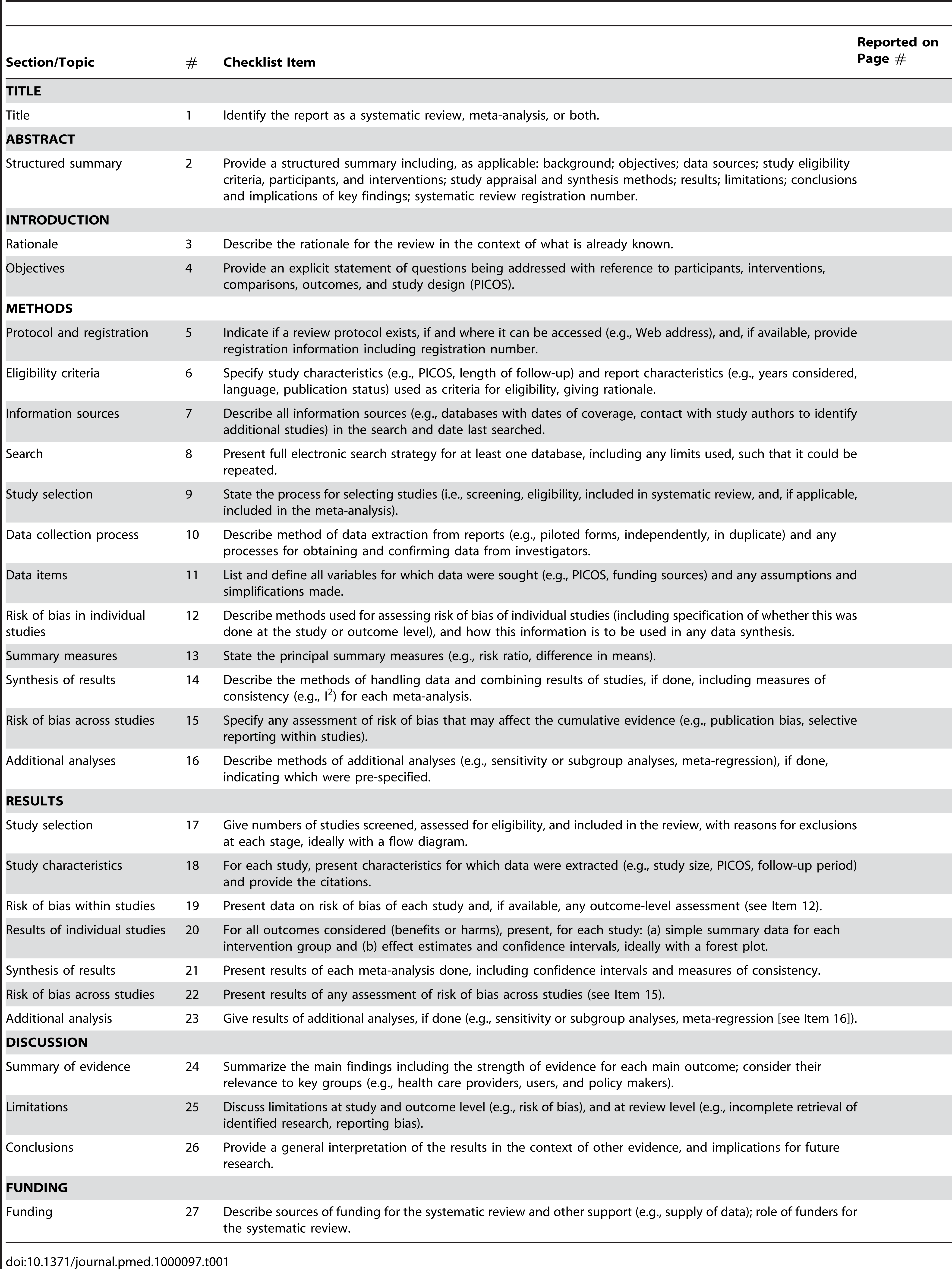


| **Item** | **Page** | **Item** | **Page** | **Item** | **Page** | **Item** | **Page** |
| --- | --- | --- | --- | --- | --- | --- | --- |
| 1 | 1 | 8 | 6 and appendix | 15 | None specified | 22 | No sensitivity analyses |
| 2 | 2 - 3 | 9 | 8 | 16 | None specified | 23 | No sensitivity analyses |
| 3 | 5 - 6 | 10 | 8 | 17 | 10 and figure 1 | 24 | 19 - 25 |
| 4 | 5 - 6 | 11 | 7 - 8 | 18 | 10 & 11 & Table 2 & 4 | 25 | 19 - 25 |
| 5 | 6 | 12 | 9 | 19 | 11 & 12 | 26 | 19 - 25 |
| 6 | 6 - 7 | 13 | 9 | 20 | 13 – 18, Tables 3a-f, figures 2-4 | 27 | 25 |
| 7 | 6 | 14 | 9 | 21 | 13 – 18, figures 2-4 |  |  |
