# Supplementary material for: Cardiotoxicity of anthracycline agents for the treatment of cancer: Systematic review and meta-analysis of randomised controlled trials
Source: BMC Cancer. 2010 Jun 29;10:337. doi: 10.1186/1471-2407-10-337 (PMC2907344; doi:10.1186/1471-2407-10-337)
Supplement: Additional file 3 — Table S1 Excluded studies. [file 1471-2407-10-337-S3.DOC]

**Table 1: Studies excluded from the systematic review**

| Reference | Reason for exclusion |
| --- | --- |
| Aapro M. D et al. Seminars in Oncology 1998;25(5 SUPPL. 12):7-11. | Preliminary findings |
| Abu-Khalaf MM et al.Breast Cancer Research and Treatment 2007;104(3):341-349. | Evaluates paclitaxel |
| Abu-Khalaf MM, et al.Oncology 2005;69(5):372-383. | Not RCT |
| Aisner J WV, et al. J Clin Oncol, Issn: 0732 183x. 1987;5(10):1523-1533. | Cumulative dose not reported and response different between groups |
| Akpek G, et al. Cancer 1368;86(7):1368-1376. | Not RCT |
| Al Ismail SA, et al. European journal of cancer & clinical oncology 1987;23(9):1379-84. | Numbers randomized not reported |
| Alba E, et al. Breast Cancer Research and Treatment 2003;77(1):1-8. | Not RCT |
| Alberts DS, et al. Drugs 1997;54(SUPPL. 4):30-35. | Not primary study |
| Alberts DS, et al. Oncology 1997;11(10 SUPPL. 11):54-62. | Review |
| Alberts DS, et al. Seminars in Oncology 2004;31(SUPPL. 13):53-90. | Review |
| Aleman BMP, et al. Blood 1878;109(5):1878-1886. | Cohort study |
| Allegra JC, et al. Investigational New Drugs 1985;3(2):153-61. | Dose received not reported |
| Alliot CE. et al.. Journal of Clinical Oncology 2005;23(21):4797-4799. | No relevant comparison group |
| Amadori D, et al. Breast Cancer Research and Treatment 1998;49(3):209-217. | No relevant comparison group |
| Amat S, et al. Clinical Breast Cancer 2006;7(3):262-269. | Evaluates docetaxel |
| Andersson M DS et al., Cancer treatment reports 1986;70(10):1181-6. | Control group recruitment closed due to safety issues |
| Andersson M, et al.European Journal of Cancer 1999;35(1):39-46. | Tamoxifen |
| Andre M, et al. Hematology and Cell Therapy 1997;39(2):59-65. | Not RCT |
| Andre M,. et al. Blood 1222;103(4):1222-1228. | Cohort study |
| Anonymous m. et al. Journal of Clinical Oncology 1991;9(2):305-12. | Different doses epirubicin evaluated |
| Anonymous m. et al. Journal of Clinical Oncology 2000;18(17):3115-24. | Different doses epirubicin based schedules evaluated |
| Antman K, et al. Journal of Clinical Oncology 1984;2(6):601-8. | Preliminary results |
| Aoki S, T et al. Journal of Experimental and Clinical Cancer Research 1998;17(4):465-470. | Dose received not reported, response different in the two groups |
| Assikis V, et al. Cancer 2003;97(11):2716-2723. | Evaluates tamoxifen |
| Augustson BM, et al. Journal of Clinical Oncology 2005;23(36):9219-9226. | Review |
| Aviles A, et al. Leukemia and Lymphoma 1997;25(3-4):319-325. | Different doses compred |
| Aviles A, et al. European Journal of Haematology 2002;68(3):144-9. | Evaluates addition of radiotherapy |
| Aviles A, et al. Leukemia and Lymphoma 2001;42(4):631-637. | Review |
| Aviles A, et al. Annals of Hematology 2002;81(7):368-373. | High versus low dose |
| Aviles A, et al. Cancer Research Therapy and Control 2000;10(1-2):119-122. | Not RCT |
| Aviles A, et al. Cancer Biotherapy and Radiopharmaceuticals 2007;22(2):194-199. | No anthracycline group |
| Aviles A, et al. Anti Cancer Drugs 1997;8(10):937-42. | Evaluates dose escalation |
| Aviles A,. et al. Leukemia and Lymphoma 2000;39(3-4):311-319. | Review |
| Aviles A, et al. Leukemia & Lymphoma 1023;46(7):1023 | Does not report treatment received |
| Aviles A,. et al. Medical & Pediatric Oncology 1995;24(3):171-5. | Duplicate of Aviles 1994 included |
| Bailey NP, et al. Annals of Oncology 1998;9(6):633-638. | Weekly versus cyclical therapy |
| Baldini E, et al. Clinical Breast Cancer 2004;5(5):358-363. | Secondary report of RCT, no cardiotoxicity outcomes |
| Baldini E,. et al. Annals of Oncology 2003;14(2):227-232. | Standard dose versus dose dense therapy |
| Baldini E, et al. British Journal of Cancer 2004;91(1):45-9. | Evaluates paclitaxel |
| Baldini E, et al. American society of clinical oncology 2003;22:37. | Evaluates paclitaxel |
| Ballova V, et al. Annals of Oncology 2005;16(1):124-131. | No non-doxorubicin arm |
| Barni S, et al. Anticancer Research 2007;27(4 C):3019-3024. | Not RCT |
| Basser RL, et al. Journal of Clinical Oncology 2006;24(3):370-8. | Dose intense versus standard dose |
| Bastholt L, et al. Journal of Clinical Oncology 1146;14(4):1146-1155. | Dose response |
| Bates M,.et al. Clinical Therapeutics 1997;19(1):167-184. | Duplicate Swain 1997 – pharmacoeconomic analysis |
| Batist G, et al. Cancer Res 1985;45(11 Pt 2):5900-3. | Not all patients randomised, not all on anthracyclines, no cardiac outcomes |
| Becher R, et al. Seminars in Oncology 1996;23(3 Suppl 7):28-33. | No appropriate control group |
| Bennett JM,. et al. Investigational New Drugs 1985;3(2):179-85. | Interim results, no numbers reported for cardiotiotoxicity, may be Bennet 1988 |
| Bentzen SM, et al. Journal of Clinical Oncology 2007;25(26):4096-4103. | Review |
| Berruti A, et al. Journal of Clinical Oncology 2002;20(20):4150-4159. | No non-anthracycline arm |
| Bertini M, et al. Haematologica 1997;82(3):309-13. | No cardiac outcomes |
| Beslija S,. Annals of Oncology 2007;18(2):215-225. | Consensus treatment guidelines |
| Bessell EM, et al. Annals of Oncology 2003;14(2):258-267. | Insufficient data reported on cardiotoxicity as not prospectively collected |
| Bianco Ar CA, et al. Cancer Chemotherapy & Pharmacology 1985;14(Suppl 1):S6. | Unavailable from British Library |
| Biganzoli L, et al. Critical Reviews in Oncology/Hematology 2007;61(1):84-89. | Retrospective analysis looking at age as a predictor of outcome |
| Biganzoli L, et al. Cancer 2003;97(1):40-5. | Evaluates paclitaxel |
| Blade J, et al. Blood 2005;106(12):3755-3759. | High versus standard dose |
| Blajman C, et al. Cancer 1091;85(5):1091-7. | No appropriate control arm |
| Bliss Jm Annals of Oncology 1990;1(Suppl):20. | Evaluates tamoxifen |
| Blum RH. et al.. Oncology 1669;11(11):1669-1677. | Secondary report |
| Bonadonna G, et al. Jama 1995;273(7):542-7. | Cumulative dose not reported |
| Bonneterre J, et al. Journal of Clinical Oncology 2005;23(12):2686-93. | Dose response study |
| Bonneterre J,. et al. Journal of Clinical Oncology 2004;22(15):3070-3079. | Dose response study, long term follow-up, observational |
| Bonneterre J, et al. Oncology 2004;66(3):185-191. | Evaluates paclitaxel |
| Bonneterre JM, et al. Oncology 2004;18(14 Suppl 14):56-8. | Dose response |
| Bontenbal M, et al. Journal of Clinical Oncology 2005;23(28):7081-8. | Evaluates docetaxel |
| Brain EGC, et al. Journal of the American Medical Association 2005;293(19):2367-2371. | Evaluates docetaxel |
| Bramwell VH, et al. European Journal of Cancer & Clinical Oncology 1983;19(8):1097-104. | Patients crossover treatment groups if indicated and cardiotoxicity reported for both treatment periods combined |
| Brandt L,. et al. Acta Oncologica 2001;40(2-3):185-197. | Systematic Review |
| Bria E, et al.. Breast Cancer Research and Treatment 2008;109(2):231-239. | No anthracycline group |
| Brufman G, et al. Annals of Oncology 1997;8(2):155-162. | High dose versus low dose |
| Brugiatelli M, et al. Haematologica 1993;78(5):306-12. | Preliminary results |
| Bryant J, et al. British Journal of Cancer 2007;96(2):226-230. | Systematic review |
| Bryant J, et al. Health Technology Assessment 2007;11(27). | Systematic review |
| Burgert EO, Jr. et al.,. Journal of Clinical Oncology 1990;8(9):1514-24. | Does not report treatment recieved |
| Burton C,. et al. British Journal of Cancer 2006;94(6):806-813. | No appropriate comparison group |
| Burton C, et al. British Journal of Haematology 2005;130(4):536-41. | CHOP versus CIOP, inferior efficacy, RCT terminated early |
| Büyükünal E et al. Chemioterapia : 1987;6(5):377-9. | Clinical trial, not randomised, baseline imbalances in prior chemo- and radiotherapy |
| Buzdar AU, et al.. Journal of Clinical Oncology 1992;10(10):1540-6. | Evaluates addition of alpha interferon |
| Buzdar AU, et al.. Journal of Clinical Oncology 1999;17(11):3412-3417. | Evaluates paclitaxel |
| Buzdar AU, Clinical Cancer Research 2007;13(1):228-233. | Evaluates paclitaxel |
| Buzzoni R, et al. Journal of Clinical Oncology 1991;9(12):2134-40. | Evaluates one dosing stategy versus another |
| Cameron A, et al. Cochrane Database of Systematic Reviews 2008;1. | Protocol for systematic review |
| Campos S. et al. Community Oncology 2005;2(SUPPL. 1):8-16. | Review |
| Cantu MG, et al. Journal of Clinical Oncology 1232;20(5):1232-1237. | Evaluates paclitaxel |
| Capotorto AM, et al. Journal of Chemotherapy 2003;15(2):184-191. | Time intense therapy versus standard therapy |
| Carde P, et al. Journal of Clinical Oncology 1993;11(11):2258-72. | No appropriate comparison group |
| Cardinale D,. Circulation 2006;114(23):2474-81. | Not all patients on anthracyclines – mix of chemotherapy regimens |
| Carrio I, et al. J Nucl Med 1993;34(9):1503-7. | Not RCT |
| Carmo-Pereira J, et al. British Journal of Cancer 1987;56(4):471-3. | Two doses compared |
| Carotenuto M et al. Leuk Lymphoma 1992;7(Suppl. 2):25-28. | Preliminary results |
| Casper ES. et al. Clin Trials J 1987;24(Suppl. 1):57-67. | Duplicate of Casper 1987b |
| Casper ES. et al. Clin. Trials J. 1987;24(Suppl. 1):139-141. | Insufficient information on doses received |
| Cassier PA,. et al. Breast Cancer Research and Treatment 2008;109(2):343-350. | Docetaxel versus paclitaxel |
| Chan S, et al. Journal of Clinical Oncology 1999;17(8):2341-54. | Evaluates docetaxel |
| Chan Sm. et al. Oncology 1997;11(8 Suppl 8):19-24. | Evaluates docetaxel |
| Chang Ae. et al. Journal of clinical oncology : 1988;6(9):1491-500. | High versus low dose |
| Chang P et al. Procamerasscancer Res 1975;16(66):No.326. | No appropriate control group |
| Colozza M, et al. European Journal of Cancer 2002;38(17):2279-2288. | Tamoxifen also given |
| Cottin Y, et al. Clinical Cardiology 1998;21(9):665-670. | Not RCT |
| Cowan JD,. et al. Investigational New Drugs 1985;3(2):149-52. | Interim results of an RCT |
| Cresta S, et al. Annals of Oncology 2004;15(3):433-9. | Evaluates docetaxel |
| Dando TM,. et al. American Journal of Cancer 2005;4(3):193-206. | Review |
| De Lena M et al. Clinical Trials Journal 1987;24(Suppl 1):223-229. | Cardiac outcomes not reported fully |
| De Lena M, et al. Medical Oncology & Tumor Pharmacotherapy 1989;6(2):163-9. | Duplicate De Lena 1987 |
| Di Trolio R, et al. International Journal of Immunopathology and Pharmacology 2006;19(2):253-263. | Systematic review |
| Dogliotti L, et al. Journal of Clinical Oncology 1165;14(4):1165-1172. | No appropriate non anthracycline group |
| Dranitsaris G, et al.. Breast Cancer Research and Treatment 2008;107(3):443-450. | Models risk from O’Brien RCT |
| Duchateau Pharmaceutisch Weekblad 1155;134(33):1155-1158. | Secondary report of a study |
| Ec van Dalen et al. Cochrane Database of Systematic Reviews: Protocols 2007 Issue 3 John Wiley & Sons, Ltd Chichester, UK DOI: 10.1002/14651858.CD006647 2007(3). | Systematic review |
| Ejlertsen B, et al. European Journal of Cancer 2007;43(5):877-84. | Not received from British Library |
| el Mawla Ng. et al. Anti cancer drugs 1991;2(4):371-4. | Dose comparison study |
| Engert A, et al.. Journal of Clinical Oncology 2007;25(23):3495-3502. | Evaluates addition of chemo to radiotherapy |
| Erkisi M, et al. Journal of Chemotherapy 1997;9(6):442-445. | Treatment received in each group unclear |
| Fenk R,. et al. British Journal of Haematology 2005;130(4):588-594. | Dose comparison study |
| Frasci G, et al. Oncology 2005;68(4-6):391-397. | Evaluates docetaxel |
| French Adjuvant Study Group. Journal of Clinical Oncology 2001;19(3):602-11. | Dose comparison study |
| Frei BL, et al. Soefje SAE.. Journal of Pharmacy Practice 2008;21(2):146-158. | Review |
| Fumoleau P, et al.. Journal of Clinical Oncology 2003;21(6):1190-1191. | Letter |
| Fumoleau P, et al. Drugs 1993;2:38-45. | Dose comparison study |
| Fumoleau P et al. Proceedings of the American Society of Clinical Oncology 2001;20(Pt 1):28a. | Evaluates docetaxel |
| Fumoleau P, et al. l. Journal of Clinical Oncology 2003;21(2):298-305. | Dose comparison study |
| Galetta F, et al. Biomedicine & Pharmacotherapy 2005;59(10):541-4. | Insufficient data for cardiac outcomes |
| Ganz Pa et al. Journal of clinical oncology : 2008;26(8):1223-30. | Long term follow up of selected patients from RCTs, observational study |
| Gianni AM, et al. New England Journal of Medicine 1290;336(18):1290-1297. | Dose comparison study |
| Giordano SH, et al.. Journal of the National Cancer Institute 2007;99(5):340-341. | Editorial |
| Giordano SH, et al. Journal of the National Cancer Institute 2008;100(4):230-231. | Editorial |
| Gill PS, et al.. Journal of Clinical Oncology 1996;14(8):2353-64. | Kaposi’s sarcoma |
| Glenn J, et al. Cancer 1206;55(6):1206-14. | Cardiotoxicity for RCT and non-RCT patients combined |
| Glenn J, et al. Surgery 1985;97(3):316-25. | Cardiotoxicity for RCT and non-RCT patients combined |
| Gregory RK, et al. European Journal of Cancer 2000;36(4):503-507. | Not RCT |
| Guglielmi C, et al.. Haematologica 1989;74(6):563-9. | Duplicate study of 145 |
| Hamberg P, et al.. European Journal of Cancer 1514;43(10):1514-1528. | Review |
| Hardenbergh PH, et al. International Journal of Radiation Oncology, Biology, Physics 1999;45(1):69-72. | Chemotherapy and radiotherapy |
| Hasan SP, et al. Journal of the National Medical Association 2004;96(2 SUPPL):196-199. | Retrospective analysis |
| Healey Bird BRJ, et al. Clinical Cancer Research 2008;14(1):14-24. | Review |
| Heidemann E, et al. Onkologie 1990;13(1):24-7. | Duplicate Heidemann 1993 |
| Hequet O, et al. Journal of Clinical Oncology 1864;22(10):1864-1871. | Not RCT |
| Iarussi D,. et al. Molecular Aspects of Medicine 1994;15(12). | Acute lymphoblastic leukemia |
| Iscoe N,. et al. Current Oncology 2003;10(1):27-35. | Kaposi’s sarcoma |
| Jensen BV. et al. Seminars in Oncology 2006;33(SUPPL. 8):15-21. | Editorial – no primary data |
| Jurcut R, et al. Supportive Care in Cancer 2008;16(5):437-445. | Review |
| Keiling R, et al. Onkologie 1986;9 Suppl 1:8-10. | Duplicate FESG 1988 |
| Kinoshita T, et al.. International Journal of Hematology 2004;80(4):341-350. | Dose comparison study |
| Kleeberg URE. et al. Gynakologische Praxis 2008;32(1):134-136. | Review |
| Knight E et al. Procamerasscancer Res 1975;16(66):No.766. | Dose comparison study |
| Kremer L C et al. Annals of Oncology 2002;13(6):819-829. | Systematic review |
| Krupicka J, et al.. Leukemia & Lymphoma 2002;43(12):2325-9. | Before and after comparison in participants from RCTs |
| Leonard Rc et al. Journal of clinical oncology : 1987;5(7):1056-63. | Cross over study, no useable outcomes |
| Linden HM, et al. Journal of Clinical Oncology 2007;25(6):656-661. | Sequential versus simultaneous dosing |
| Lipshultz SE, et al. Heart 2008;94(4):525-533. | Not primary study, narrative review |
| Lopez M, et al.. Cancer 1989;64(12):2431-6. | Dose received not reported, difference in response |
| Macioch T, et al. Family Medicine and Primary Care Review 2006;8(3):997-999. | Systematic review |
| Lopez M,. Oncology 1989;46(1):1-5. | Duplicate of IMBSWE 1988 |
| Magnus B et al., European Journal of Haematology 2008;80(6):477-482. | Meta-analysis |
| Mano M,. et al. Breast Cancer Research and Treatment 2005;89(1):81-90. | Review |
| Martoni A, et al. Oncology 1990;47(5):427-32. | Cross overs and dose received not clear |
| Massidda B, et al. Anticancer Research 1997;17(1B):663-8. | Cardiac function not defined |
| Maung K, et al. Clinical Breast Cancer 2002;3(3):183-184. | Secondary report of RCT |
| Mellqvist UH, et al. Cancer 2008;112(1):129-135. | Not an appropriate comparison group |
| Merli F, et al. Leukemia and Lymphoma 2007;48(2):367-373. | No appropriate comparison group |
| Meyer RM, et al. Journal of Clinical Oncology 1995;13(9):2386-93. | Weekly versus standard dosing |
| Mori M, et al.. International Journal of Hematology 2005;81(3):246-54. | No appropriate comparison group |
| Michelotti A, et al. Breast Cancer Research & Treatment 2000;59(2):133-9. | Duplicate Venturini 1996 |
| Mori M, et al. Leukemia and Lymphoma 2001;41(3-4):359-366. | Not RCT |
| Moser EC, et al. N Clinical Lymphoma & Myeloma 2005;6(2):122-30. | Not RCT |
| Mouridsen HT, et al. Eur J Cancer Clin Oncol 1987;23(10):1477-83. | No cardiotoxicity outcomes |
| Murphy CA, et al. Drug Safety 2007;30(9):783-804. | Review |
| Muss HB, et al. Cancer 1982;50(11):2269-74. | No appropriate comparison group |
| Nair R, et al. Cancer 1998;82(11):2282-8. | Treatment received unclear |
| Nakamae H,. et al. Cancer 2005;104(11):2492-8. | Not an RCT |
| Namer M et al. European journal of cancer (Oxford, England : 1990) 2001;37(9):1132-40. | No appropriate comparison group |
| Neidhart JA, et al. Journal of Clinical Oncology 1986;4(5):672-7. | Patients crossover treatment groups if indicated and cardiotoxicity reported for both treatment periods combined |
| Neidhart JA, et al. Cancer Treatment Reviews 1983;10:41-6. | Patients crossover treatment groups if indicated and cardiotoxicity reported for both treatment periods combined |
| Neidhart JA, et al. Seminars in Oncology 1984;11(3 Suppl 1):11-4. | Preliminary results of Niedhart 1986 |
| Nemoto T, et al. Cancer 1978;41(6):2073-7. | No appropriate comparison group |
| Nielsen D, et al. Cancer Chemotherapy & Pharmacology 2000;46(6):459-66. | No appropriate comparison group |
| Nielsen OS, et al. British Journal of Cancer 1998;78(12):1634-9. | High dose regimen |
| Nielsen OS, et al. Sarcoma 2000;4(1-2):31-35. | Duplicate of Nielsen 1998 |
| Osby E, et al. Blood 2003;101(10):3840-8. | No cardiac outcomes reported |
| Palumbo A, et al. Annals of Oncology 1160;19(6):1160-1165. | Not RCT |
| Pangalis GA, et al. Leukemia & Lymphoma 2003;44(4):635-44. | No cardiac outcomes |
| Parmar Mkb et al. Lancet 1998;352(9140):1571-6. | No cardiac outcomes, just reports that they were ‘rare’ |
| Parveen S,. et al. Journal of the College of Physicians and Surgeons Pakistan 2002;12(5):292-296. | No cardiotoxicity outcomes |
| Pavone V, et al. Annals of Oncology 2008;19(4):763-8. | No appropriate control group |
| Perez EA, et al. Journal of Clinical Oncology 2004;22(18):3700-3704. | Before and after comparison, no non anthracycline comparison |
| Pfreundschuh M, et al.. Blood 2004;104(3):634-641. | No appropriate control group |
| Phillips JK, et al. Leukemia & Lymphoma 1995;17(5-6):465-72. | No useable data for cardiotoxicity, gives mean treatment received in those with cardiotoxicity, not number of patients |
| Pfreundschuh M, et al. Annals of Oncology 2008;19(3):545-552. | Dose comparison study |
| Piccart MJ. et al. Annals of Oncology 2001;12(SUPPL. 1):S89-S94. | Guidelines |
| Pignata S, et al. BMC Cancer 2006;6(202). | Evaluates paclitaxel |
| Pinedo Hm, et al. Clin Trials J 1987;24(Suppl. 1):231-241. | No cardiac outcomes |
| Piver MS, et al. European Journal of Gynaecological Oncology 1998;19(1):5-10. | Evaluates paclitaxel |
| Ploner F, et al. Onkologie 2003;26(2):115-119. | No cardiac outcomes |
| Raynov J, et al. Archive of Oncology 1997;5(1):15-16. | Not RCT |
| Roche H, et al. Journal of Clinical Oncology 2006;24(36):5664-71. | Evaluates docetaxel |
| Rosenfeld CS et al. Proceedings of ASCO 1992;Vol. 11:62. | Duplicate Swain 1997 |
| Rozencweig M, et al. Journal of Clinical Oncology 1984;2(4):275-81. | No appropriate comparison group |
| Santoro A. et al. Journal of clinical oncology :1995;13(7):1537-45. | No appropriate comparison group |
| Schoenfeld Da et al. 1982;50(12):2757-2762. | Cardiotoxicity not an outcome |
| Semiglazov Vf et al. Voprosy onkologii 2000;46(2):160-6. | Russian |
| Speyer JL, et al. M. New England Journal of Medicine 1988;319(12):745-52. | Duplicate Speyer 1992, no additional data |
| Speyer JL, et al. Cancer Treatment Reviews 1990;17(2-3):161-3. | Duplicate Speyer 1992, no additional data |
| Stebbing J, et al. Breast Cancer Research and Treatment 2008;107(3):451-453. | Editorial |
| Stewart DJ, Seminars in Oncology 1984;11(3 Suppl 1):23-7. | Preliminary results |
| Stohr W, et al. Journal of Cancer Research and Clinical Oncology 2006;132(1):35-40. | Long term followup of participants in RCT but not all patients |
| Sukel MPP,. et al. Pharmacoepidemiology and Drug Safety 2008;17(2):125-134. | Observational study |
| Sutton GP, et al.. Journal of Clinical Oncology 1989;7(2):223-9. | No appropriate control arm – one dose versus another |
| Taguchi Tet al. [Gan to Kagaku Ryoho [Japanese Journal of Cancer & Chemotherapy] 1986;13(9):2820-8. | Japanese language |
| Tallarico D et al. Angiology 2003;54(2):219-227. | Unclear if randomised |
| Tebbi CK,et al. Journal of Clinical Oncology 2007;25(5):493-500. | No cardiac outcomes |
| Tirelli U,. et al. Critical Reviews in Oncology/Hematology 2001;37(2):153-158. | Review |
| Tirelli U, et al. Journal of Clinical Oncology 1998;16(1):27-34. | No appropriate comparison group |
| Torti FM, et al. Ann Intern Med 1983;99(6):745-9. | Not an RCT |
| Tranum Bl 1982;49(5):835-839. | No appropriate comparison group |
| Tsimberidou AM, et al. Blood 2002;100(13):4351-4357. | No cardiac outcomes |
| Tsurumi H, et al. Journal of Cancer Research and Clinical Oncology 2004;130(2):107-113. | Treatment received not clear, difference in response |
| Unverferth DV, et al. Semin Oncol 1983;10(1 Suppl 1):49-52. | No cardiac outcomes |
| Uthayakumar S, et al. Aids 1996;10(5):515-9. | Kaposi’s sarcoma |
| Van Dalen EC, et al. Cochrane Database of Systematic Reviews 2006;4. | Systematic review |
| Van Toorn DW, et al. Breast Cancer Research and Treatment 2000;60(1):57-62. | Dose comparison study |
| Ventura GJE. et al. Journal of Clinical Oncology 2005;23(12). | Letter – no primary data |
| Verdonck LF, et al. Blood 2007;109(7):2759-2766. | Dose comparison study |
| Vici P,. Clinica Terapeutica 1998;149(921):15-20. | Duplicate Lopex 1998 |
| Weisberg SR et al. Proceedings of ASCO 1992;;Vol. 11:91. | Duplicate Swain 1997 |
| Wilson Re. et al. Archives of surgery (Chicago, Ill. : 1960) 1986;121(11):1354-9. | Does not report treatment received |
| Wolff AC, et al. Surgical Oncology 1999;8(2):93-101. | Review |
