# Supplementary material for: Cardiotoxicity of anthracycline agents for the treatment of cancer: Systematic review and meta-analysis of randomised controlled trials
Source: BMC Cancer. 2010 Jun 29;10:337. doi: 10.1186/1471-2407-10-337 (PMC2907344; doi:10.1186/1471-2407-10-337)
Supplement: Additional file 4 — Table S2 Characteristics of included studies. [file 1471-2407-10-337-S4.DOC]

**Table 2: Participant characteristics and treatment schedules**

**Anthracycline chemotherapy versus non-anthracycline chemotherapy**

| **Study** | **Participants** | **Interventions** | **Schedule** |
| --- | --- | --- | --- |
| Ackland 2001 [24] | Women aged 18-70 years with metastatic breast cancer, no prior anthracycline, WHO performance status 0-2, LVEF normal for institution, no cardiac disease or ECG abnormalities | CEF | Cyclophosphamide 400 mg/m^2^ IV, epirubicin 50 mg/m^2^, 5-FU 500 mg/m^2^ IV days 1 and 8, cycles repeated every 3-4 weeks maximum 6 cycles, median cycles received 6 (range 1-9) |
|  |  | CMF | Cyclophosphamide 500 mg/m^2^ IV, methotrexate 40 mg/m^2^, 5-FU 600 mg/m2 IV days 1 and 8, cycles repeated every 3-4 weeks, maximum 6 cycles, median cycles received 6 (range 1-10) |
| Feher 2005 [25] | Women aged > 60 years with metastatic breast cancer, no prior anthracyclines, non-anthracycline therapy and/or radiotherapy allowed, Karnofsky performance ≥ 60, no active cardiac disease | Epirubicin | 35 mg/m^2^ IV days 1, 8 and 15 of 28 day cycle, maximum 8 cycles (840 mg/m^2^). Lifetime cumulative dose not to exceed 900 mg/m^2^ or 750 mg/m^2^ if radiotherapy to mediastinal area, median cumulative dose 340 mg/m^2^ (range 120-390) |
|  |  | Gemcitabine | 1200 mg/m^2^ IV days 1, 8 and 15 of 28 day cycle, maximum 12 cycles, median cumulative dose 1100 mg/m^2^ (range 343-1266) |
| Hernadi 1988 [28] | Women aged < 70 years, stage III or IV ovarian cancer, no previous irradiation or chemotherapy. | CAP | 600 mg/m^2^ cyclophosphamide + 60 mg/m^2^ cisplatin + 60 mg/m^2^ epirubicin at 3 week intervals for up to 10 cycles OR 70 mg/m^2^ doxorubicin bolus injection at 3 week intervals. Reassessed after 3 cycles. total number of cycles decided by physician, median 8 cycles received both groups |
|  |  | CEP |  |
|  |  | CP | 1000 mg/m^2^ cyclophosphamide + 60 mg/m^2^ cisplatin at 3 week intervals for up to 10 cycles, median 9 cycles received |
| Levine 2005 [26] | Women with node positive breast cancer, no cardiac disease | CEF | Cyclophosphamide 75 mg/m^2^ PO days 1 - 14, epirubicin 60 mg/m^2^ IV days 1 and 8, 5-FU 500 mg/m^2^ IV days 1 and 8, median cumulative dose 607.9 mg/m^2^ |
|  |  | CMF | Cyclophosphamide 100 mg/m^2^ PO days 1 - 14, methotrexate 40 mg/m^2^ IV days 1-8 5-FU 600 mg/m^2^ IV days 1 and 8, median cumulative dose 469.8 mg/m^2^ |
| Martin 2003 [27] | Women aged 18 to 72 years with operable breast cancer, no cardiac disease (MI, cardiac insufficiency, arrhythmia) | FAC | 5-FU 500 mg/m^2^ day 1, doxorubicin 50 mg/m^2^ day 1, cyclophosphamide 600 mg/m^2^, day 1 every 3 weeks for 6 cycles, 87% received planned dose |
|  |  | CMF | Cyclophosphamide 600 mg/m^2^, day 1, methotrexate 60 mg/m^2^ day 1, 5-FU 600 mg/m^2^ day 1 every 3 weeks for 6 cycles, 85% received planned dose |
| Sposto 2001 [29] | Children aged < 21 years with NLB NHL | Daunomycin-COMP | Two phase chemotherapy 30 day induction and 15 cycles maintenance. Daunomycin 50 mg/m^2^ on day 15 of induction, and day 14 for first 6 cycles. All other chemotherapy identical in both arms |
|  |  | COMP |  |
| Sweetnam 1986 [31] | Adults or children with osteosarcoma treated by amputation or radiotherapy, aged ≤ 40 years, no metastases | Doxorubicin  Vincristine  Methotrexate | Vincristine 2 mg/m^2^ (max 2 mg), methotrexate 200 mg/m^2^, doxorubicin 60 mg/m^2^, folinic acid rescue every 6 weeks for maximum of 54 weeks |
|  |  | Vincristine  Methotrexate | Vincristine 2 mg/m^2^ (max 2 mg), methotrexate 200 mg/m^2^, folinic acid rescue every 3 weeks for maximum of 54 weeks |
| Sullivan 1991[30] | Children aged < 18 years, with Hodgkin's lymphoma, no prior therapy or major illnesses | A-COPP + radiotherapy | Cyclophosphamide substituted for Mustargen and preceded by adriamycin 6 cycles with radiotherapy sandwiched between second and third courses, adriamycin 60 mg/m^2^ all patients received >= 90% therapy |
|  |  | MOPP + bleomycin + radiotherapy |  |
| **Anthracycline chemotherapy versus mitoxantrone** | | | |
| Alonso 1995 [32] | Women aged ≤ 75 years with metastatic breast cancer, ECOG 0-3, no prior anthracyclines or radiotherapy to left chest, no CHF or hypertension, LVEF normal | CAF | Cyclophosphamide 600 mg/m^2^, 5-FU 600 mg/m^2^, adriamycin 50 mg/m^2^ IV every 3 weeks, mximum 10 cycles, |
|  |  | CMF | Cyclophosphamide 600 mg/m^2^, 5-FU 600 mg/m^2^, mitoxantrone 12mg/m^2^ IV every 3 weeks, mximum 10 cycles, |
| Aviles 1994 [45] | Adults with stage IIIB to IVB Hodgkin's disease, ECOG 0-2, no prior treatment, no CHF, LVEF > 50% | EVBD | Epirubicin 25 mg/m^2^ IV, vinblastine 6 mg/m^2^, bleomycin 15 mg/m^2^ days 1 and 8, every 28 days, maximum number cycles 9 received by all patients |
|  |  | MVBD | Mitoxantrone 5 mg/m^2^ IV, vinblastine 6 mg/m^2^, bleomycin 15 mg/m^2^ days 1 and 8, every 28 days, maximum number cycles 9 received by all patients |
| Bennett 1988 [33] | Women with metastatic breast cancer, Zubrod 0-2, prior adjunct chemotherapy allowed, no prior anthracycline, prior radiotherapy allowed, no cardiac disease, LVEF normal | CAF | Cyclophosphamide 500 mg/m^2^, 5-FU 500 mg/m^2^, doxorubicin 50 mg/m^2^ IV every 3 weeks, maximum dose 450 mg/m^2^ , dose received decreased over time from 96% to 68% of initial planed dose |
|  |  | CNF | Cyclophosphamide 500 mg/m^2^, 5-FU 500 mg/m^2^,mitoxantrone 10 mg/m^2^ IV every 3 weeks, no upper dose limit, dose received decreased over time from 99% to 71% of initial planed dose |
| Cavo 2002 [43] | Adults with symptomatic multiple myeloma, excluded if > 80 years or severe heart disease | VAD/MP | Vincristine 0.4 mg/d, doxorubicin 9 mg/d days 1-4, dexamethazone 40 mg/d for 4 days, Courses repeated every 28 days for 8 courses, mean dose intensity 0.86 for doxorubicin |
|  |  | VND/MP | Identical to VAD except mitoxantrone substituted for doxorubicin 3mg/m^2^/d days 1-4. Courses repeated every 28 days for 8 courses, mean dose intensity 0.86 for mitoxantrone |
| Cook 1996 [34] | Women aged < 75 years with advanced or metastatic breast cancer, Karnofsky ≥ 60, no prior chemotherapy or evidence of heart failure | Epirubicin | 75 mg/m^2^ every 3 weeks for maximum duration of 1 year or disease progression, then crossed over to other drug |
|  |  | Mitoxantrone | 14 mg/m^2^ very 3 weeks for maximum duration of 1 year or disease progression, then crossed over to other drug |
| Esteban 1999 [35] | Women aged < 75 years with metastatic breast cancer, Karnofsky ≥ 50, normal LVEF, ECG, prior chemotherapy allowed, no prior anthracyclines, no prior heart disease | CEF | Cyclophosphamide 100 mg/m^2^ PO days 1-14, epirubicin 30 mg/m^2^ days 1 and 8, fluorouracil 500 mg/m^2^ IV days 1 and 8 every 4 weeks until unacceptable toxicity or progression, median cumulative dose 506 mg/m^2^ (range 44-1.096) |
|  |  | CNF | Cyclophosphamide 100 mg/m^2^ PO days 1-14, mitoxantrone 6 mg/m^2^ days 1 and 8, fluorouracil 500 mg/m^2^ IV days 1 and 8 every 4 weeks until unacceptable toxicity or progression, median cumulative dose 61.5 mg/m^2^ (range 8-162) |
| Follezou 1987 [36] | Women with advanced breast cancer, normal ejection fraction, no cardiac disease | CAF | Cyclophosphamide 600 mg/m^2^, 5-FU 750 mg/m2, adriamycin 45 mg/m^2^ every 21 days |
|  |  | CNF | Cyclophosphamide 600 mg/m^2^, 5-FU 750 mg/m^2^, mitoxantrone 13 mg/m^2^ every 21 days |
| Gherlinzoni 1990 [44] | Adults with stage II-IV Non-Hodgkin's lymphoma, previously untreated, ECOG 0-2, no CHF or IHD within 2 years, resting LVEF ≥ 50% (echo) | m-BACOD | Cyclophosphamide 600 mg/m^2^, Bleomycin 4 mg/m^2^, vincristine 1 mg/m^2^, dexamethazone 6 mg/m^2^, methotrexate 200 mg/m^2^, leucovorin 15 mg/m^2^ PO plus doxorubicin 45 mg/m^2^, 60% received 10 cycles |
|  |  | m-BNCOD | Cyclophosphamide 600 mg/m^2^, Bleomycin 4 mg/m^2^, vincristine 1 mg/m^2^, dexamethazone 6 mg/m^2^, methotrexate 200 mg/m^2^, leucovorin 15 mg/m^2^ PO plus mitoxantrone 10 mg/m^2^, 70% received 10 cycles |
| Hausmaninger 1995 [37] | Women aged ≤ 75 years with metastatic breast cancer, Karnofsky ≥ 50, no prior anthracyclines, no cardiac problems, | Epirubicin + vindesine | Epirubicin 40 mg/m^2^, vindesine 3 mg/m^2^ every 3 weeks for 3 cycles then every 4 weeks, maximum cumulative epirubicin dose 1000 mg/m^2^, cycles received (range 1-17) |
|  |  | Mitoxantrone + vindesine | Mitoxantrone 10mg/m^2^, vindesine 3 mg/m^2^ every 3 weeks for 3 cycles then every 4 weeks, maximum cumulative mitoxantrone 160 mg/m^2^, cycles received (range 1-21) |
| Henderson 1989 [39] | Women with metastatic breast cancer, Zubrod 0-2, prior adjunct therapy allowed, no prior anthracycline, prior radiotherapy allowed, normal LVEF, no cardiac disease | Doxorubicin | 75 mg/m^2^ IV every 3 weeks, crossover to mitoxantrone if progression, maximum dose 450 mg/m^2^ |
|  |  | Mitoxantrone | 14 mg/m^2^ every 3 weeks, crossover to doxorubicin if progression, no upper dose limit |
| Lawton 1993 [40] | Women with breast cancer with proven local recurrence or distant metastases, WHO 1-3; previous adjuvant chemotherapy allowed provided it had been given at least 3 months previously. | Doxorubicin | 70 mg/m^2^ doxorubicin bolus injection at 3 week intervals, reassessed after 3 cycles, total number of cycles decided by physician total cycles given 120, range per person 1-8 |
|  |  | Epirubicin | 70 mg/m^2^ epirubicin bolus injection at 3 week intervals, reassessed after 3 cycles, total number of cycles decided by physician, total cycles given 153, range per person 1-10 |
|  |  | Mitoxantrone | 14 mg/m^2^ mitoxantrone bolus injection at 3 week intervals, reassessed after 3 cycles, total number of cycles decided by physician, total cycles given 31, range per person 1-15 |
| Heidemann 1993 [38] | Women with breast cancer aged ≤ 66 years, WHO ≤ 2, LVEF normal, no prior adjuvant treatment (≥ 6 months) with anthracycline, clinical signs of cardiac insufficiency or cardiac arrhythmias, or MI within 6 months | Adriamycin  cyclophosphamide | Adriamycin 40 mg/m^2^ + 600 mg/m^2^ of cyclophosphamide every 3 weeks until max cumulative dose reached (550 mg/m^2^). |
|  |  | Epirubicin  Cyclophosphamide | Epirubicin 40 mg/m^2^ + 600 mg/m^2^ of cyclophosphamide every 3 weeks until max cumulative dose reached (1000 mg/m^2^). |
|  |  | Mitoxantrone  Cyclophosphamide | Mitoxantrone 12 mg/m^2^ + 600mg/m^2^ of cyclophosphamide every 3 weeks until max cumulative dose reached (200 mg/m^2^). |
| Pavlovsky 1992 [46] | Aged > 15 years with intermediate or high grade lymphoma, no prior treatment, performance status 0-2, no previous MI | CHOP | Cyclophosphamide 750 mg/m^2^, vincristine 1.4 mg/m^2^, doxorubicin 50 mg/m^2^ day 1 and prednisone 50 mg/m^2^ days 1 to 5. Given every 21-28 days for 6 cycles |
|  |  | CNOP | Cyclophosphamide 750 mg/m^2^, vincristine 1.4 mg/m^2^, mitoxantrone 10 mg/m^2^ day 1 and prednisone 50 mg/m^2^ days 1 to 5. Given every 21-28 days for 6 cycles |
| Periti 1991 [41] | Women with advanced or metastatic breast cancer aged ≤ 75 years, WHO ≤ 2, normal cardiac function, LVEF ≥ 50%, prior chemotherapy allowed, no prior anthracyclines, , prior radiotherapy allowed, no cardiac dysfunction within last 6 months | FEC | Cyclophosphamide 500 mg/m^2^, fluorouracil 500 mg/m^2^, epirubicin 50 mg/m^2^ once every 3 weeks, minimum 3 courses, maximum 12 courses, median cumulative dose 330 mg/m^2^ |
|  |  | FNC | Cyclophosphamide 500 mg/m^2^, fluorouracil 500 mg/m^2^, mitoxantrone 10 mg/m^2^ once every 3 weeks, minimum 3 courses, maximum 12 courses, median cumulative dose 52 mg/m^2^ |
| Stewart 1997 [42] | Women with advanced or metastatic breast cancer, prior adjuvant chemo allowed, no prior anthracyclines, prior radiotherapy allowed, ECOG 0-2, no history heart disease, LVEF at least 50% | CAF | Cyclophosphamide 500 mg/m^2^, fluorouracil 500 mg/m^2^, doxorubicin 50 mg/m^2^, courses every 3 weeks, maximum cumulative dose 450 mg/m^2^ |
|  |  | CMF | Cyclophosphamide 500 mg/m^2^, fluorouracil 500 mg/m^2^, mitoxantrone 50 mg/m^2^, courses every 3 weeks, no defined upper dose limit mitoxantrone |

**Bolus versus continuous infusion**

| **Study** | **Participants** | **Interventions** | **Schedule** |
| --- | --- | --- | --- |
| Casper 1991 [49] | Adults with primary or recurrent non-metastatic soft tissue sarcoma, prior radiotherapy permitted, no prior chemotherapy, history heart disease or abnormal LVEF | Doxorubicin bolus | 60 mg/m^2^ every 3 weeks for 9 cycles, initiated after surgical healing and acute radiation injury, median cumulative dose 420 mg/m^2^ (range 60 to 540 mg/m^2^), 37% received complete planned dose of 540mg/m^2^ |
|  |  | Doxorubicin continuous infusion | 60 mg/m^2^ every 3 weeks for 9 cycles 72 hour continuous infusion initiated after surgical healing and acute radiation injury, Cumulative range 120 to 540 mg/m^2^, 64% completed the planned course |
| Zalupski 1991 [50] | Adults with soft-tissue sarcoma with metastatic disease, Karnofsky score 50-100, normal cardiac function. Concurrent radiotherapy not permitted (radiotherapy more than 1 month prior to study entry was allowed) | Doxorubicin + dacarbazine bolus injection. | On day 1, patients received bolus treatment of doxorubicin 60 mg/m^2^ and dacarbazine 750 mg/m^2^, every 3 weeks. Patients determined to have stable disease or partial response after 3 or 6 cycles of treatment were eligible for cytoreductive surgery. Following recovery from surgery, chemotherapy was resumed until total dose of 450 mg/m^2^ was reached. Responders could receive doxorubicin more than 450, but LVEF monitored closely, median cumulative dose 240 mg/m^2^ |
|  |  | Doxorubicin + dacarbazine continuous infusion | Doxorubicin 60 mg/m^2^ and dacarbazine 750 mg/m^2^ infusion for 96 hours then same as above, median cumulative dose 221 mg/m^2^ |
| Shapira 1990 [48] | Women with metastatic breast or advanced ovarian cancer, stage III or IV Excluded women with history of valvular or coronary heart disease. | Doxorubicin bolus | Short infusion over 15 to 20 minutes of doxorubicin 50 mg/m^2^ + cyclophosphamide 500 mg/m^2^ + 5-fluorouracil 500 mg/m^2^ or cisplatin 50 mg/^2^. Mean cumulative dose doxorubicin received 410 mg/m^2^ |
|  |  | Doxorubicin continuous infusion | Prolonged infusion over 6 hrs of doxorubicin 50 mg/m^2^ + cyclophosphamide 500 mg/m^2^ + 5-fluorouracil 500 mg/m^2^ or cisplatin 50 mg/^2^. Mean cumulative dose doxorubicin received 428 mg/m^2^ |
| Hortobagyi 1989 [47] | Women with metastatic breast cancer treated with four of fewer prior chemotherapy regimens; previous anthracycline treatment allowed, normal LVEF or endocardial biopsy grade 2 or lower and no history of major cardiac disease | Epirubicin bolus | 90 mg/m^2^ of epirubicin as a 15 min infusion (bolus) every 21 days. Median cumulative dose 540 mg/m^2^ includes previous therapy (420 mg/m^2^ in doxorubicin equivalent) |
|  |  | Doxorubicin continuous infusion | 60 mg/m^2^ of doxorubicin as a 48 hr continuous infusion repeated every 21 days. Median cumulative dose 630 mg/m^2^ including previous therapy 70 mg/m^2^ |
|  |  | Epirubicin continuous infusion | 90 mg/m^2^ of epirubicin as a 48 hr continuous infusion repeated every 21 days. Median cumulative dose 630 mg/m^2^ includes previous therapy (560 mg/m^2^ doxorubicin equivalent) |

One anthracycline versus another

| **Study** | **Participants** | **Interventions** | **Schedule** |
| --- | --- | --- | --- |
| Bezwoda 1986 [58] | Women aged ≤70 years with stage IIB, III, IV ovarian cancer, no prior chemotherapy, excluded if aged > 70 years, no history of CHF. | PAC | Cisplatin 20 mg/m^2^ IV daily x 3, adriamycin 40mg/m^2^ IV x 1, cyclophosphamide 600 mg/m^2^ IV x 1 every 4 weeks for 12 cycles. |
|  |  | PEC | Cisplatin 20 mg/m^2^ IV daily x 3, 4'epi-adriamycin 50 mg/m^2^ IV x 1, cyclophosphamide 600 mg/m^2^ IV x 1 every 4 weeks for 12 cycles. |
| Bontenbal 1998 [52] | Women with metastatic breast cancer, WHO > 3, no prior anthracyclines, CHF, serious arrhythmia, bilateral bundle branch block or history of MI | Doxorubicin | 75 mg/m^2^, every 3 weeks, if no response within 4 cycles could cross over to alternative therapy, cumulative dose received 383 mg/m^2^ (47-911) |
|  |  | Epirubicin | 90 mg/m^2^ every 3 weeks, if no response within 4 cycles could cross over to alternative therapy, cumulative dose received 447 mg/m^2^ (88-1452) |
| Brambilla 1986 [53] | Women with advanced breast cancer with inoperable disease or distant metastases, Karnofsky ≥ 60, no history of MI, no cardiac arrhythmias, LVEF > 50%, radiotherapy to chest possible | Doxorubicin | 75 mg/m^2^ given bolus IV injection, every 3 weeks up to a max cumulative dose of 600 mg/m^2^ in absence of tumour progression, median cumulative dose received 540 mg/m^2^ |
|  |  | Epirubicin | 75 mg/m^2^ given bolus IV injection, every 3 weeks up to a max cumulative dose of 600 mg/m^2^ in absence of tumour progression, median cumulative dose received 565 mg/m^2^ |
| FESG 1988 [51] | Women with advanced breast cancer with metastatic or recurrent disease aged ≤ 70 years, performance status ≤2, no prior anthracycline or pre-existing heart disease, chest radiotherapy possible. | Fluorouracil 500mg/m^2^, cyclophosphamide 500mg/m^2^, doxorubicin 50mg/m^2^ (FAC) | Treatment cycles repeated every 21 days with all three drugs administered IV on day 1 of each cycle. Treatment to progression, unacceptable toxicity or cumulative dose 550 mg/m^2^ for doxorubicin or 720 mg/m^2^ for epirubicin. Mean dose received 368 (+/- 30), 28 received 550 mg/m2 (n=113) doxorubicin, mean dose received 382 (+/- 37) 20 received 720 mg/m^2^ (n=117) epirubicin |
|  |  | Fluorouracil 500mg/m^2^, cyclophosphamide 500mg/m^2^, epirubicin 50mg/m^2^ (FEC) |  |
| Gasparini 1991 [54] | Women with advanced breast cancer, Karnofsky score ≥ 50. No history of MI, angina pectoris, cardiac arrhythmias, hypertension, or prior anthracycline treatment. Prior chest radiotherapy to chest possible. | Doxorubicin | 20 mg/m^2^ every week given by bolus IV injection until cumulative max dose reached (550 mg/m^2^), median cumulative dose received 240 mg/m^2^ range (160-860), number receiving > 450 mg/m^2^ = 3 (>550 mg/m2) |
|  |  | Epirubicin | 20mg/m^2^ every week given by bolus IV injection until cumulative max dose reached (550 mg/m^2^), median cumulative dose received 220 mg/m^2^ (range 160-620), number receiving > 450 mg/m^2^ = 2 (>550 mg/m^2^) |
| Heidemann 1993 [38] | Women with breast cancer, age ≤ 66 years, WHO performance status ≤ 2, minimum expected survival ≥ 3 months. LVEF normal, excluded if prior adjuvant treatment (≥ 6months) with anthracycline, clinical signs of cardiac insufficiency or cardiac arrhythmias, MI within 6 months | Adriamycin  cyclophosphamide | Adriamycin 40 mg/m^2^ + 600 mg/m^2^ of cyclophosphamide every 3 weeks until max cumulative dose reached (550 mg/m^2^). |
|  |  | Epirubicin  Cyclophosphamide | Epirubicin 40 mg/m^2^ + 600 mg/m^2^ of cyclophosphamide every 3 weeks until max cumulative dose reached (1000 mg/m^2^). |
| Hernadi 1988 [28] | Women aged < 70 years with stage III or IV ovarian cancer. No previous irradiation or chemotherapy. | CAP | 600 mg/m^2^ cyclophosphamide + 60 mg/m^2^ cisplatin + 60 mg/m^2^ adriamycin at 3 week intervals for up to 10 cycles, median cycles received 8 |
|  |  | CEP | 600 mg/m^2^ cyclophosphamide + 60 mg/m^2^ cisplatin + 60 mg/m^2^ epirubicin at 3 week intervals for up to 10 cycles, median cycles received 8 |
| Homesley 1992 [59] | Women, 18-75 years, with stage III, IV epithelial ovarian cancer, with no prior chemotherapy or radiation, excluded if MI in previous 6 months, active ischemic heart disease, uncontrolled hypertension, evidence of heart failure | Doxorubicin + cisplatin | Cisplatin 60 mg/m^2^, doxorubicin 60 mg/m^2^ to be given every 4 weeks for a maximum of eight courses, 73% received planned dose |
|  |  | Epirubicin + cisplatin | Cisplatin 60 mg/m^2^, epirubicin 75 mg/m^2^ to be given every 4 weeks for a maximum of eight course, 77% received planned dose |
| IMBSWE 1988 [57] | Women with advanced breast cancer. Karnofsky >40; and aged < 75 yrs, excluded participants with myocardiopathy, presence of brain metastases, and previous treatment with anthracyclines, radiotherapy to chest possible | FAC | Treatment cycles repeated every 21 days with all three drugs administered IV on day 1 of each cycle and flurouracil 500 mg/m^2^ on day 8 also. Patient continued on treatment until disease progression or cumulative dose 550 mg/m^2^ for doxorubicin, median cumulative dose 311 mg/m2, number receiving > 450 mg/m^2^ = 77 (31.2%) |
|  |  | FEC | Treatment cycles repeated every 21 days with all three drugs administered IV on day 1 of each cycle and flurouracil 500 mg/m^2^ on day 8 also. Patient continued on treatment until disease progression or cumulative dose 700 mg/m^2^ for epirubicin, median cumulative dose 330 mg/m^2^, number receiving > 450 mg/m^2^ = 74 (29.6%) |
| Jain 1985 [55] | Women with advanced breast cancer, Karnofsky ≥ 50%, no prior anthracycline treatment, active cardiac disease or LVEF < 50%, radiotherapy to chest possible | Doxorubicin | 60 mg/m^2^ IV every 3 weeks, 86% received planned dose |
|  |  | Epirubicin | 85 mg/m^2^ IV every 3 weeks, 100% received planned dose |
| Lahtinen 1991 [60] | Non-Hodgkin lymphoma patients in whom CHOP therapy was indicated. No previous history, symptoms or signs of heart disease, radiotherapy to chest possible, no prior anthracyclines | CHOP | Cyclophosphamide 750 mg/m^2^, doxorubicin 50 mg/m^2^, vincristine 1.4 mg/m^2^, prednisolone 100 mg/m^2^, every 3 weeks until max cumulative dose (500 mg/m^2^). |
|  |  | CEOP | Cyclophosphamide 750 mg/m^2^, epirubicin 50 mg/m^2^, vincristine 1.4 mg/m^2^, prednisolone 100 mg/m^2^, every 3 weeks until max cumulative dose (500 mg/m^2^) |
| Lawton 1993 [40] | Patients with breast cancer who had proven local recurrence or distant metastases, WHO 1-3; previous adjuvant chemotherapy did not exclude patients provided it had been given at least 3 months previously. | Doxorubicin | 70 mg/m^2^ doxorubicin bolus injection at 3 week intervals. Reassessed after 3 cycles. Total number of cycles decided by physician - 120 total cycles given |
|  |  | Epirubicin | 70 mg/m^2^ epirubicin bolus injection at 3 week intervals. Reassessed after 3 cycles. Total number of cycles decided by physician - 153 total cycles given |
| Perez 1991 [56] | Women with advanced breast cancer, metastatic disease, adequate cardiac function confirmed by ECG or scan. No prior chemotherapy including adjuvant chemotherapy | Doxorubicin | 60 mg/m^2^ IV every 3 weeks for at least 6 cycles, max cumulative dose 550 mg/m^2^, median cycles received 5 (range 1 to 12) |
|  |  | Epirubicin | 90 mg/m^2^ IV every 3 weeks for at least 6 cycles. No limit on cumulative dose allowed, median cycles received 5, (range 1 to 9) |
| **Liposomal doxorubicin versus non-liopsomal doxorubicin** | | | |
| Batist 2001 [61] | Women with metastatic breast cancer ECOG ≥ 2, resting LVEF ≥ 50% and no documented history of CHF, serious arrhythmia or myocardial infarction (within 6 months). Adjuvant chemotherapy, including doxorubicin if the cumulative dose did not exceed 300 mg/m^2^, was allowed if more than 6 months had elapsed. Prior radiation was permitted if the dose to the mediastinal area did not exceed 35 Gy and no more than 50% of the bone marrow was involved. Women were excluded if they had other serious medical risk factors | Liposomal doxorubicin + cyclophosphamide | Liposome-encapsulated doxorubicin 60 mg/m^2^ with cyclophosphamide 600 mg/m^2^ every 3 weeks. Median cumulative dose 360 mg/m^2^ range (60-2220mg/m^2^), number receiving > 500 mg n=33 |
|  |  | Doxorubicin + cyclophosphamide | Conventional doxorubicin 60 mg/m^2^ with cyclophosphamide 600 mg/m^2^. Median cumulative dose 360 mg/m^2^ (60-660 mg/m^2^), number receiving > 500 mg n=9 |
| Harris 2002 [62] | Women with metastatic breast caner; ECOG 0-2; resting LVEF ≥ 50%; adjuvant doxorubicin up to a maximum lifetime dose of 300 mg/m^2^ allowed, but no adjuvant treatment with other anthracycline or anthracenediones; Excluded if any chemotherapy within 6 months of entering the study; patients who had prior radiation > 3500 centigrays (cGy) to the mediastinal area or radiation to greater than 50% of the bone marrow; a history of CHF, serious cardiac arrhythmia or MI previous 6 months | Liposomal doxorubicin | Liposomal doxorubicin 75 mg/m^2^ every 3 weeks, mean dose intensity 25.4 mg/m2/week, number receiving > 500 mg/m^2^, n=75, |
|  |  | Conventional doxorubicin | Conventional doxorubicin 75 mg/m^2^ every 3 weeks, mean dose intensity 26.3 mg/m^2^/week, number receiving > 500 mg/m^2^, n=51 |
| O'Brien 2004 [63] | Women ≥ 18 yrs of age with metastatic breast cancer, WHO ≤ 2 stages IIIB or IV MBC, prior hormonal or adjuvant anthracycline therapy permitted with a cumulative doxorubicin (or doxorubicin equivalent) dose of ≤ 300 mg/m^2^ and an adjuvant chemotherapy-free interval of > 12 months. Normal cardiac (LVEF) function, excluded if history of ischaemic heart disease or arrhythmia requiring treatment, clinically significant valvular disease or LVEF below LLN for institution, radiotherapy to chest possible | Pegylated liposomal doxorubicin (PLD) | 50 mg/m^2^ PLD infusion for up to 60 min every 4 weeks, cumulative dose 398 mg/m^2^ in those included in cardiotoxicity analysis |
|  |  | Doxorubicin | 60 mg/m^2^ Doxorubicin infusion for 60 mins every 3 weeks, cumulative dose 421 mg/m^2^ in those included in cardiotoxicity analysis. |
| Rifkin 2006 [64] | Adults with untreated multiple myeloma, Karnofsky score ≥ 60%, excluded if NYHA grade II cardiac disease, MI in past 6 months, unstable angina, hypertension, cardiac arrhythmias | Pegylated liposomal doxorubicin | 40 mg/m^2^ IV over 1 hour, vincristine 1.4 mg/m^2^ to a max of 2.0 mg, dexamethazone 40 mg/day po 1-4 of each cycle, total 474 cycles received |
|  |  | Doxorubicin | 9 mg/m^2^ over 96 hours, vincristine 0.4 mg/day, dexamethazone 40 mg/po days 1-4, total 420 cycles received |
| **Liposomal doxorubicin versus non-liopsomal epirubicin** | | | |
| Chan 2004 [65] | Women with breast cancer with measurable metastatic disease; ECOG ≤ 2; resting LVEF ≥ 50%, excluded if had a history of significant cardiac problems. Prior radiotherapy to chest possible. | Liposomal doxorubicin + cyclophosphamide | Liposomal doxorubicin 75 mg/m^2^ plus cyclophosphamide 600 mg/m^2^ every 3 weeks for up to 8 cycles. Number receiving > 500 mg/m^2^, n=47 |
|  |  | Epirubicin + cyclophosphamide | Epirubicin 75 mg/m^2^ plus cyclophosphamide 600 mg/m^2^ every 3 weeks for up to 8 cycles. Number receiving > 500 mg/m^2^, n=49 |
| **Doxorubicin or epidoxorubicin versus idarubicin** | | | |
| Federico 1998a [67] | Adults and children with intermediate-grade (IG) or high-grade (HG) NHL other than lymphoblastic lymphoma; no prior treatment; clinical stage II, III and IV or clinical stage I with bulky disease; age over 12 yrs; patients over 70 yrs were also included on the basis of good performance status and in absence of underlying coronary artery or pulmonary disease. | ProME(epidoxorubicin)CE-CytaBOM (PE-C)  cyclophosphamide + etoposide + epidoxorubicin | 650 mg/m^2^ cyclophosphamide + 120 mg/m^2^ etoposide + 30 mg/m^2^ epidoxorubicin on day 1. 60 mg/m^2^ prednisolone on days 1 to 14, 300 mg/m^2^ cytarabine, 5 mg/m^2^ bleomycin, 1.4 mg/m^2^ vincristine, 120 mg/m^2^ methotrexate on day 8 with 10 mg/m^2^ leucovorin for 5 doses beginning 24 hrs after methotrexate administration, cycles repeated every 3 weeks for 6 planned cycles, 94% received planned dose |
|  |  | Pro-MI(idarubicin)CE-CytaBOM (PI-C)  cyclophosphamide + etoposide + idarubicin | 650 mg/m^2^ cyclophosphamide + 120 mg/m^2^ etoposide + 6 mg/m^2^ idarubicin on day 1. 60 mg/m^2^ prednisolone on days 1 to 14, 300 mg/m^2^ cytarabine, 5 mg/m^2^ bleomycin, 1.4 mg/m^2^ vincristine, 120 mg/m^2^ methotrexate on day 8 with 10 mg/m^2^ leucovorin for 5 doses beginning 24 hrs after methotrexate administration, cycles repeated every 3 weeks for 6 planned cycles, 93% received planned dose |
| Zinzani 1995 [66] | 18- 70 years old, intermediate grade non-Hodgkin's lymphoma (stage II-IV), LVEF > 50% | CHOP | Cyclophosphamide 750 mg/m^2^, doxorubicin 50mg/m^2^, vincristine 1.4 mg/m^2^ on day 1, prednisolone 100 mg/m^2^ on days 1 to 5, every 3 weeks for 8-10 courses, 71% received planned dose |
|  |  | CIOP | Cyclophosphamide 750 mg/m^2^, idarubicin 10 mg/m^2^, vincristine 1.4 mg/m^2^ on day 1, prednisolone 100 mg/m^2^ on days 1 to 5, every 3 weeks for 8-10 courses, 68% received planned dose |

**Anthracycline plus chemoprotective agent versus anthracycline**

| **Study** |  | **Interventions** | **Schedule** |
| --- | --- | --- | --- |
| **Dexrazoxane** | | | |
| Lopez 1998 [73] | Advanced breast or soft tissue sarcoma, aged 14 to 75 years, WHO ≤ 3, resting LVEF ≥ 45%. Excluded patients with cardiac disease (MI, CHF, angina pectoris) other malignancies, CNS involvement, prior anthracycline use, prior radiotherapy >20 Gy on mediastinal area possible | Epirubicin + dexrazoxane | Epirubicin 160 mg/m^2^ by IV every 3 weeks, dexrazoxane 1000 mg/m^2^ IV over 15 mins , 30 mins before epirubicin. Epirubicin given for a max of 8 cycles discontinued when disease progressed, unacceptable toxicity or patient refusal. Median cumulative dose 960 mg/m^2^ |
|  |  | Epirubicin | Median cumulative dose 880 mg/m^2^ |
| Marty 2006 [68] | Women >18 years with advanced/metastatic breast carcinoma; prior anthracycline exposure but anthracycline-free for at least 6 months prior study start; LVEF LLN for the centre, prior radiotherapy possible | Anthracycline (doxorubicin or epirubicin) based combination chemotherapy + dexrazoxane (20:1 dextrazoxane:doxorubicin dose ratio, or 10:1 epirubicin dose ratio) | Chemotherapy treatment cycles repeated every 3 weeks. Patients with stable disease, or better, continued assigned treatment for a maximum of 6 cycles. Treatment to progression, CHF, cardiac event or unacceptable toxicity. 30 minutes before infusion of the anthracycline, dexrazoxane was infused over approx 15 min. Dexrazoxane was given from the first dose for a minimum of two cycles. Median cumulative dose 669 (range 247-936) mg/m^2^ |
|  |  | Anthracycline (doxorubicin or epirubicin) based combination chemotherapy | Median cumulative dose 608 (244-900) mg/m^2^ |
| Speyer 1992 [69] | Women with advanced breast cancer, prior adjuvant cyclophosphamide, methotrexate and 5FU (CMF) if terminated at least 6 months before randomisation. LVEF > 0.5, no prior anthracyclines, chest radiotherapy possible | FDC + dexrazoxane 1000 mg/m^2^ | 5FU 500mg/m^2^ administered by IV bolus, doxorubicin 50 mg/m^2^ administered by slow 5-10 min IV infusion, cyclophosphamide 500 mg/m^2^, dexrazoxane 1000 mg/m^2^ admin 30 mins before FDC over 15 mins 20:1 dexrazoxane to doxorubicin, Median cumulative dose 500 mg/m^2^ |
|  |  | FDC | Median cumulative dose 441 mg/m^2^ |
| Swain 1997 [70] | Women aged ≥ 18 years with stage IIIB or IV breast cancer, prior adjuvant chemo allowed if non-anthracycline and finished at least 6 months prior to study start. ECOG 0-2, LVEF > LLN for institution. No prior anthracyclines, CHF or cardiomyopathy, current arrhythmia or MI within 6 months, prior chest radiotherapy possible | FDC + dexrazoxane | FDC administered on 1st day of each treatment, dexrazoxane given 50 mL/m^2^ 30 mins before doxorubicin, every 3 weeks, 10:1 ratio of dextrazoxane:doxorubicin, (initial ratio 20:1 but changed by committee due to higher number of deaths in dexrazoxane arm) |
|  |  | FDC + placebo |  |
| Venturini 1996 [71] | Women with advanced, locally advanced IIIB or inflammatory breast cancer, ECOG 0-2. LVEF ≥ 50%. Prior anthracyclines and chest radiotherapy possible | CEF+ dexrazoxane or high dose epirubicin + dexrazoxane | **Patients who DID receive prior anthracyclines:** cyclophosphamide 600 mg/m^2^ (IV), epirubicin 60 mg/m^2^ (IV) and fluorouracil 600 mg/m^2^ (IV) on day 1 given every 3 weeks (CEF) and dexrazoxane 600 mg/m^2^ 30 mins prior to epirubicin (10:1 dextrazoxane:epirubicin ratio) **Patient who DID NOT receive prior anthracyclines:**  high dose epirubicin 120 mg/m^2^ IV on day 1 given every 3 weeks and dexrazoxane 1,200 mg/m^2^ 30 mins prior HD-Epi  Median cumulative dose 720 mg/m^2^ |
|  |  | CEF+ placebo or high dose epirubicin + placebo | Median cumulative dose 720 mg/m^2^ |
| Wexler 1996 [72] | Adults or children aged ≤ 25 years with one of Ewing's family of sarcomas, no prior anthracyclines or cardiac radiotherapy | Doxorubicin + dexrazoxane 20:1 | Doxorubicin 70 mg/m^2^ cycles 1,3, 5 with vincristine 2 mg/m^2^ max 2 mg, cyclophosphamide 1,800 mg/m^2^, cycles 9, 11, 13 and 15 doxorubicin 50 mg/m^2^. vincristine 2 mg/m^2^ and cyclophosphamide 1200 mg/m^2^, cycles 2,4,8,7,8,10,12,14,16,17,18 ifosfamide 1,800 mg/m^2^/d plus etoposide 100 mg/m^2^/d. 20:1 dextrazoxane:doxorubicin ratio given 15 minutes before doxorubicin. Cycles repeated every 3 weeks, median cumulative dose doxorubicin 410 (range 140-410) mg/m^2^ |
|  |  | Doxorubicin | Median cumulative dose doxorubicin 310 (range 70 - 410) mg/m^2^ |
| **L-Cartinine** | | | |
| Waldner 2006 [75] | Patients with non-Hodgkin lymphoma. | CHOP + L-cartinine | Chemotherapy consisted of 6 CHOP cycles (day 1: cyclophosphamide 750 mg/m^2^, vincristine 1.4 mg/m^2^, max. 2mg; doxorubicin 50 mg/m^2^; days 2-5: prednisolone 100 mg p.o.), 3 g L-cartinine before each chemotherapy cycle IV, followed by oral administration of 1 g L-cartinine /day during the following 21 days. Cumulative doxorubicin dose 600 mg/m^2^ both groups. |
|  |  | CHOP + placebo |  |
| **Carvedilol** | | | |
| Kalay 2006 [74] | Malignancy (breast, lymphoma and other). No prior chemotherapy, radiotherapy, CHF symptoms or established dilated or restrictive CMP, coronary arterial disease history, moderate or severe mitral or aortic valve disease in ECG or other co-morbidity | ANT + carvedilol | Carvedilol 12.5 mg once daily. Cumulative doxorubicin and epirubicin dose 525.3 mg/m^2^ and 787.9 mg/m^2^  Cumulative doxorubicin and epirubicin dose 525.3 mg/m^2^ and 787.9 mg/m^2^ |
|  |  | ANT + placebo | Cumulative doxorubicin and epirubicin dose 513.6 mg/m^2^ and 770.4 mg/m^2^ |
| **Prenylamine** | | | |
| Milei 1987 [76] | Adults with breast, ovarian, lung, renal, pancreatic or colon cancer. No prior chest radiotherapy, or risk factors for myocardiopathy | Adriamycin +  Prenylamine | ADM 40-50 mg/m^2^ every 3 weeks IV + PNL 200mg/ day PO until cumulative max dose ADM reached (550 mg/m^2^). Median cumulative dose received 400 mg/m^2^ |
|  |  | Adriamycin | Median cumulative dose received 360 mg/m^2^ |
| **Amifostine** | | | |
| Gallegos-Castorena 2007 [77] | Children with osteosarcoma and no prior therapy received | Cisplatin + doxorubicin + amifostine | Cisplatin 150 mg/m^2^ every 2 weeks, IV doxorubicin 75 mg/m^2^ every 4 weeks before surgery, 4 cycles, amifostine 740 mg/m^2^ 15 min IV infusion before cisplatin, cumulative doxorubicin dose both groups 150 mg/m^2^ |
|  |  | Cisplatin + doxorubicin |  |
| **N-acetylcycsteine** | | | |
| Meyers 1983 [78] | Breast cancer, lymphoma, metastatic soft tissue sarcoma or other tumour, may have received prior radiotherapy up to a max of 600 rads, no prior cardiac dysfunction. | Doxorubicin + N-acetylcysteine | Doxorubicin 75 mg/m2 + N-acetylcysteine 5.5 gm/m^2^ PO before doxorubicin, only patients who received at least 4 cycles analysed both groups |
|  |  | Doxorubicin |  |

Abbreviations: A-COPP= cyclophosphamide , vincristine, prednisone, procarbazine ANT= Adriamycin or epirubicin; CAF= cyclophosphamide, adriamycin, 5-FU; CEF= cyclophosphamide, epirubicin, 5-FU; CMF= cyclophosphamide, methotrexate, 5-FU; CAP= cyclophosphamide, cisplatin, adriamycin; CEP= cyclophosphamide, cisplatin, epirubicin; CP= cyclophosphamide, cisplatin; CHF= congestive heart failure; CEOP= CHOP= cyclophosphamide, vincristine, doxorubicin, prednisone; COMP= cyclophosphamide, vincristine, methotrexate, prednisone; CNOP= cyclophosphamide, vincristine, mitoxantrone, prednisone; COMP=; CMF= cyclophosphamide, fluorouracil, mitoxantrone; CNF= cyclophosphamide, 5-FU, mitoxantrone; ECG=Electrocardiogram; ECOG=European Cooperative Oncology Group; EVBD=epirubicin, vinblastine, bleomycin; MVBD=mitoxantrone, vinblastine, bleomycin FAC=fluorouracil, cyclophosphamide, doxorubicin; FEC= Fluorouracil, cyclophosphamide, epirubicin; FDC=Fluorouracil (5FU), doxorubicin, cyclophosphamide; IV=intravenous; FNC= cyclophosphamide, fluorouracil, mitoxantrone; LVEF=Left Ventricular Ejection Fraction; MI= myocardial infarction; MOPP=mustargen, vincristine, prednisone, procarbazine; M-BACOD= cyclophosphamide, bleomycin, vincristine, dexamethazone, methotrexate, leucovorin, doxorubicin; m-BNCOD= cyclophosphamide, bleomycin, vincristine, dexamethazone, methotrexate, leucovorin, mitoxantrone; NLBNHL=non-lymphoblastic non-Hodgkins lymphoma; PAC=Cisplatin, Adriamycin, cyclophosphamide; PEC=Cisplatin, 4'epi-adriamycin, Cyclophosphamide; PO=oral; VAD = vincristine, doxorubicin, dexamethazone; VND/MP= vincristine, mitoxantrone, dexamethazone; WHO=World Health Organization;
