# Supplementary material for: Cardiotoxicity of anthracycline agents for the treatment of cancer: Systematic review and meta-analysis of randomised controlled trials
Source: BMC Cancer. 2010 Jun 29;10:337. doi: 10.1186/1471-2407-10-337 (PMC2907344; doi:10.1186/1471-2407-10-337)
Supplement: Additional file 5 — Table S3 Risk factors for cardiotoxicity in included studies. [file 1471-2407-10-337-S5.DOC]

**Table 3: Risk factors for cardiotoxicity in RCTs evaluating anthracyclines**

| Study | Age: Mean, SD | Age: Range | Gender: n, (%) Male | Postmenopausal n (%) | Prior treatment with anthracyclines n (%) | Prior radiation therapy n (%) | Prior radiation therapy to chest area n (%) | Prior adjuvant chemo n (%) | Prior exposure to unspecified chemotherapy n (%) | Smoking status n (%) | Diabetes n (%) | Prior history hypertension n (%) | Prior antihypertensive treatment n (%) | Prior history of cardiac disease n (%) | Cardiac risk factors n (%) |
| --- | --- | --- | --- | --- | --- | --- | --- | --- | --- | --- | --- | --- | --- | --- | --- |
| **Continuous versus bolus** | | | | | | | | | | | | | | | |
| Casper 1991 [49] | 56 | 25-87 | 20 (53) |  |  | 18 (47) |  |  | 0 |  |  |  |  | 0 |  |
|  | 51 | 18-76 | 23 (52) |  |  | 19 (43) |  |  | 0 |  |  |  |  | 0 |  |
| Hortobagyi 1989 [47] | 56 | 28-74 |  | 23 | 21 |  |  |  |  |  |  |  |  |  | 21 |
|  | 48 | 30-67 |  | 11 | 12 |  |  |  |  |  |  |  |  |  | 14 |
| Shapira 1990 [48] | 55 |  |  |  |  |  |  |  |  |  |  |  |  | 0 |  |
|  | 53 |  |  |  |  |  |  |  |  |  |  |  |  | 0 |  |
| Zalupski 1991 [50] | 53 | 26-69 | 62 |  |  |  |  |  |  |  |  |  |  |  |  |
|  | 52 | 26-67 | 65 |  |  |  |  |  |  |  |  |  |  |  |  |
| **Cardioprotective agent versus none** | | | | | | | | | | | | | | | |
| Lopez 1998 [73] |  | 28-75 | sar 11 (61) | 38 (84) |  | breast 10 (22), sar 8 (44) |  | 19 (42) |  |  |  |  |  |  |  |
|  |  | 14-75 | sar 9 (56) | 44 (88) |  | breast 8 (16), sar 5 (31) |  | 18 (36) |  |  |  |  |  |  |  |
| Marty 2006 [68] | 50 | 31-76 | (50) |  |  | 74 (87) |  |  |  | 12% |  |  |  |  |  |
|  | 52 | 30-71 | (50) |  |  | 62 (78) |  |  |  | 9% |  |  |  |  |  |
| Speyer 1992 [69] | 56 | 32-76 |  |  |  |  |  | 23 (30) |  |  |  |  |  |  | 41 (54) |
|  | 56 | 27-75 |  |  |  |  |  | 22 (30) |  |  |  |  |  |  | 43 (58) |
| Swain 1997 [70]  (08001) | 58 | 26-84 |  |  |  | 75 (45) | 20 (12) | 72 (43) |  |  | 10 (6) | 46 (27) |  |  |  |
|  | 56 | 25-82 |  |  |  | 71 (39) | 14 (8) | 63 (35) |  |  | 21 (12) | 45 (25) |  |  |  |
| (08006) | 56 | 35-76 |  | 72 (89) |  | 26 (32) | 21 (26) | 30 (37) |  |  | 5 (6) | 21 (26) |  |  |  |
|  | 60 | 23-79 |  | 83 (80) |  | 41 (39) | 36 (35) | 36 (35) |  |  | 8 (8) | 36 (35) |  |  |  |
| Venturini 1996 [71] | 57 | 32 - 73 |  | 68 (81) |  |  | 30 (36) | 28 (33) |  |  | 1 (1.2) | 8 (9.5) |  |  | 32 (38) |
|  | 57 | 34 - 74 |  | 64 (82) |  |  | 36 (46) | 22 (28) |  |  | 2 (2.6) | 11 (14.1) |  |  | 30 (39) |
| Wexler 1996 [72] | 18.5 | 4-24 |  |  |  |  |  |  |  |  |  |  |  |  |  |
|  | 15.5 | 9-24 |  |  |  |  |  |  |  |  |  |  |  |  |  |
| Gallegos-Castoreno 2007 [77] | 11.6 | 7-15 | 15 (50) |  |  |  |  |  |  |  |  |  |  |  |  |
| Kalay 2006 [74] | 46.8 (14) |  | 3 (12) |  |  |  |  |  |  |  |  |  |  |  |  |
|  | 49 (9.8) |  | 4 (16) |  |  |  |  |  |  |  |  |  |  |  |  |
| Milei 1987 [76] | 60 |  | 31 |  |  |  |  |  |  |  |  |  |  |  |  |
|  | 64 |  | 38 |  |  |  |  |  |  |  |  |  |  |  |  |
| Meyers 1983 [78] | nr |  |  |  |  |  |  |  |  |  |  |  |  |  |  |
| Waldner 2006 [75] | 66 |  |  |  |  |  |  |  |  |  |  |  |  |  | 3 |
|  | 64 |  |  |  |  |  |  |  |  |  |  |  |  |  | 3 |
| One anthracycline versus another | | | | | | | | | | | | | | | |
| Bezwoda 1986 [58] | nr |  |  |  |  |  |  |  |  |  |  |  |  |  |  |
| Brambilla 1986 | 51 | 28-69 |  |  |  |  | 1 |  |  |  |  |  |  |  |  |
|  | 53 | 31-64 |  |  |  |  | 4 |  |  |  |  |  |  |  |  |
| Bontenbal 1998 [52] | 56 | 31-75 |  | 100% | 0 |  |  |  | 115 (98) |  |  |  |  |  |  |
|  | 56 | 34-73 |  | 110 (97) | 0 |  |  |  | 110 (97) |  |  |  |  |  |  |
| FESG 1988 [51] | 54 | 26-70 |  |  |  |  | 59 (52) |  |  |  |  |  |  |  |  |
|  | 54 | 31-70 |  |  |  |  | 65 (55) |  |  |  |  |  |  |  |  |
| Gasparini 1991 [54] | 60 | 30-77 |  |  | 0 |  | 64 |  |  |  |  |  |  |  |  |
|  | 55 | 45-76 |  |  | 0 |  | 57 |  |  |  |  |  |  |  |  |
| Heidmann 1993 [38] |  |  |  | 62 |  | 32 |  | 67 |  |  |  |  |  |  |  |
|  |  |  |  | 54 |  | 31 |  | 83 |  |  |  |  |  |  |  |
|  |  |  |  | 60 |  | 46 |  | 78 |  |  |  |  |  |  |  |
| Homesley 1992 [59] | nr |  |  |  |  |  |  |  |  |  |  |  |  |  |  |
| Hernadi 1988 [28] | 50 | 30-67 |  |  |  |  |  |  |  |  |  |  |  |  |  |
|  | 51 | 29-68 |  |  | 0 | 0 |  |  |  |  |  |  |  |  |  |
|  | 49 | 23-66 |  |  | 0 | 0 |  |  |  |  |  |  |  |  |  |
| IMBSWE 1988 [57] | 53.9 | 30-74 |  | 151 (68) | 0 | 83 ( 38) |  | 61  (28) |  |  |  |  |  |  |  |
|  | 55.1 | 28-75 |  | 154 (69) | 0 | 90 (41) |  | 65 (29) |  |  |  |  |  |  |  |
| Jain 1995 [55] | 52 | 33-63 |  |  |  |  |  |  |  |  |  |  |  |  |  |
|  | 52 | 36-74 |  |  |  |  | 14 |  | 100 |  |  | 6 |  |  |  |
| Lahtinen 1991 [60] | 49 | 35-68 | 50% |  |  | 8 | 8 |  | 100 |  |  | 4 |  |  |  |
|  | 49 | 32-65 | 50% |  |  | 16 | 0 |  |  |  |  |  |  |  |  |
| Lawton 1993 [40] | 51 |  |  | 20 |  |  |  |  |  |  |  |  |  |  |  |
|  | 47 |  |  | 17 |  |  |  |  |  |  |  |  |  |  |  |
|  | 50 |  |  | 21 |  |  |  |  |  |  |  |  |  |  |  |
| Perez 1991 [56] | 57 | 32-69 |  |  |  |  |  |  |  |  |  |  |  |  |  |
|  | 53 | 25-71 |  |  |  |  |  |  |  |  |  |  |  |  |  |
| Batist 2001 [61] | 55 | 30-80 |  |  | 14 (10) | 48 (34) | 15 | 8 | 46 (32) |  |  |  |  | 5 |  |
|  | 54 | 22-88 |  |  | 15 (10), | 59 (38) | 19 | 10 | 61 (39) |  |  |  |  | 3 |  |
| Harris 2002 [62] | 58 | 26-85 |  |  | 18 (17) | 47 (44) |  |  | 43 (40) |  |  |  |  |  |  |
|  | 58 | 29-82 |  |  | 21 (18) | 51 (44) |  |  | 47 (41) |  |  |  |  |  |  |
| O’Brien 2004 [63] | 58 | 28-82 |  | 175 (68.8) | 15% | 120 (47.2) |  |  |  |  |  |  |  |  | 122 (48%) |
|  | 57 | 25-82 |  | 159 (62.3) | 16% | 126 (49.4) |  |  |  |  |  |  |  |  | 121 (47.4%) |
| Rifkin 2006 [64] | 60 | 37-84 | 59% |  |  |  |  |  |  |  |  |  |  |  |  |
|  | 60 | 44-81 | 61% |  |  |  |  |  |  |  |  |  |  |  |  |
| Chan 2004 [65] | 54 | 19-78 |  |  |  | 47 (59) |  |  | 26 (33) |  |  |  |  |  |  |
|  | 54 | 26-82 |  |  |  | 53 (66) |  |  | 28 (35) |  |  |  |  |  |  |
| Federico 1998 [67] |  |  | 82 (64) |  |  |  |  |  |  |  |  |  |  |  |  |
|  |  |  | 69 (57) |  |  |  |  |  |  |  |  |  |  |  |  |
| Zinzani 1995 [66] | 53 | 26-69 | 62% |  |  |  |  |  |  |  |  |  |  |  |  |
|  | 52 | 26-67 | 65% |  |  |  |  |  |  |  |  |  |  |  |  |
| Anthracycline versus non-anthracycline | | | | | | | | | | | | | | | |
| Ackland 2001 [24] | 55 | 26-71 |  | (75) |  |  |  | 20 |  |  |  |  |  |  |  |
|  | 55 | 22-71 |  | (69) |  |  |  | 20 |  |  |  |  |  |  |  |
| Feher 2005 [25] | 68 | 60-85 |  |  |  | 97 (48.7) |  |  | 39 (19.6) |  |  |  |  |  |  |
|  | 69 | 59-91 |  |  |  | 103 (52.0) |  |  | 39 (19.7) |  |  |  |  |  |  |
| Hernadi 1988 [28] | 50 | 30-67 |  |  |  |  |  |  |  |  |  |  |  |  |  |
|  | 51 | 29-68 |  |  | 0 | 0 |  |  |  |  |  |  |  |  |  |
|  | 49 | 23-66 |  |  | 0 | 0 |  |  |  |  |  |  |  |  |  |
| Levine 2005 [26] |  |  |  |  |  |  | 0 |  |  |  |  |  |  |  |  |
|  |  |  |  |  |  |  | 0 |  |  |  |  |  |  |  |  |
| Martin 2003 [27] | 321 > 50 |  |  | 338 | 0 | 84 |  |  |  |  |  |  |  |  |  |
|  | 298 >50 |  |  | 315 | 0 | 78 |  |  |  |  |  |  |  |  |  |
| Sposto 2001 [29] |  |  | 69% |  |  |  |  |  |  |  |  |  |  |  |  |
| Sweetnam 1986 [31] |  |  | 57% |  |  |  |  |  |  |  |  |  |  |  |  |
|  |  |  | 54% |  |  |  |  |  |  |  |  |  |  |  |  |
| Sullivan 1991 [30] | nr |  |  |  |  |  |  |  |  |  |  |  |  |  |  |
| Anthracycline versus mitoxaztrone | | | | | | | | | | | | | | | |
| Alonso 1995 [32] | 55 | 30-72 |  | 37 |  | 3 (6) |  | 14 (28) |  |  |  |  |  |  |  |
|  | 55 | 31-74 |  | 39 |  | 4 (8) |  | 6 (12) |  |  |  |  |  |  |  |
| Aviles 1994 [45] | 38 |  | 15 |  |  |  |  |  |  |  |  |  |  |  |  |
|  | 35 |  | 17 |  |  |  |  |  |  |  |  |  |  |  |  |
| Bennett 1988 [33] |  |  |  | 120 (73%) |  | 56 (34%) |  | 43 (26%) |  |  |  |  |  |  |  |
|  |  |  |  | 115 (69%) |  | 71 (43%) |  | 40 (24%) |  |  |  |  |  |  |  |
| Cavo 2002 [43] | nr |  |  |  |  |  |  |  |  |  |  |  |  |  |  |
| Cook 1996 [34] | 59 |  |  | 14 |  |  |  |  |  |  |  |  |  |  |  |
|  | 49 |  |  | 9 |  |  |  |  |  |  |  |  |  |  |  |
| Esteban 1999 [35] | 58 | 33-73 |  | 57 (78) |  | 40 (55) |  |  | 13 (18) |  |  |  |  |  |  |
|  | 58 | 30-70 |  | 60 (83) |  | 53 (74) |  |  | 19 (26) |  |  |  |  |  |  |
| Follezou 1987 [36] | 56 | 32-74 |  | 51 |  | 50 |  |  | 15 |  |  |  |  |  |  |
|  |  |  |  | 62 |  | 51 |  |  | 18 |  |  |  |  |  |  |
| Gherlinzoni 1990 [44] | 51 | 19-78 | 25 |  |  |  |  |  |  |  |  |  |  |  |  |
|  | 50 | 23-73 | 24 |  |  |  |  |  |  |  |  |  |  |  |  |
| Hausmaninger 1995 [37] | 57 | 29-75 |  | 95 (74) |  |  |  | 46 (36) |  |  |  |  |  |  |  |
|  | 56 | 27-75 |  | 97 (77) |  |  |  | 32 (25) |  |  |  |  |  |  |  |
| Henderson 1989 [39] |  |  |  | 137 (86%) |  | 98 (61%) |  | 55 (34%) |  |  |  |  |  |  |  |
|  |  |  |  | 141 (85%) |  | 86 (52%) |  | 60 (36%) |  |  |  |  |  |  |  |
| Lawton 1993 [40] | 51 |  |  | 20 |  |  |  |  |  |  |  |  |  |  |  |
|  | 47 |  |  | 17 |  |  |  |  |  |  |  |  |  |  |  |
|  | 50 |  |  | 21 |  |  |  |  |  |  |  |  |  |  |  |
| Heidemann, 1993 [38] |  |  |  | 62 |  | 32 |  | 67 |  |  |  |  |  |  |  |
|  |  |  |  | 54 |  | 31 |  | 83 |  |  |  |  |  |  |  |
|  |  |  |  | 60 |  | 46 |  | 78 |  |  |  |  |  |  |  |
| Pavlovsky 1992 [46] | 51 | 19-75 | 22 |  |  |  |  |  |  |  |  |  |  |  |  |
|  | 58 | 23-88 | 25 |  |  |  |  |  |  |  |  |  |  |  |  |
| Periti 1991 [41] |  |  |  | 26 | 0 | 6 |  |  | 7 |  |  |  |  |  |  |
|  |  |  |  | 21 | 0 | 8 |  |  | 7 |  |  |  |  |  |  |
| Stewart 1997 [42] | 57 | 31-78 |  | 81% |  | 73 (57) |  |  | 32 (25) |  |  |  |  |  |  |
|  | 58 | 23-75 |  | 85% |  | 66 (55) |  |  | 31 (26) |  |  |  |  |  |  |
