# Supplementary material for: Cardiotoxicity of anthracycline agents for the treatment of cancer: Systematic review and meta-analysis of randomised controlled trials
Source: BMC Cancer. 2010 Jun 29;10:337. doi: 10.1186/1471-2407-10-337 (PMC2907344; doi:10.1186/1471-2407-10-337)
Supplement: Additional file 6 — Table S4 Cardiotoxicity outcomes in included studies. [file 1471-2407-10-337-S6.DOC]

**Table 4: Cardiotoxicity outcomes in RCTs evaluating anthracyclines regimens**

**4a: Anthracycline chemotherapy versus non-anthracycline chemotherapy**

| **Study** | **Cardiac outcomes** | **Definition and measurement** | **Group** | number analysed | Cardiotoxic event | CHF | cumulative dose to cardiotoxic event | decrease in LVEF | death (cardiac related) | ECG changes | Discontinuation due to LVEF abnormality | **Length of follow up** | **Additional comments, and when outcomes occurred** |
| --- | --- | --- | --- | --- | --- | --- | --- | --- | --- | --- | --- | --- | --- |
| Ackland 2001 [24] | Cardiotoxicity, reduction LVEF | Cardiotoxicity defined as reduction ≥ 20% or ≥ 10% below LLN or CHF, MUGA or echo, ECG, physical | CEF | 156 |  | 5 |  | 19 |  |  |  | Median at least 20 months | no cardiac deaths |
|  |  |  | CMF | 172 |  | 0 |  | 2 |  |  |  |  | 3 CHF on, 2 off treatment |
| Feher 2005 [25] | Reduction LVEF, cardiac event of clinical concern, withdrawal due to cardiac event | Reduction LVEF to <45%, or > 45% if decrease ≥10% from baseline,  cardiac events of clinical concern, MUGA or echo | Epirubicin | 199 | 47 |  |  |  | 0 |  | 10 | 19.1 months | Death due to MI, probably early but may include late as follow-up > 1 year, unclear if on or off treatment |
|  |  |  | Gemcitabine | 198 | 34 |  |  |  | 1 |  | 1 | 11.8 months |  |
| Hernadi 1988 [28] | Cardiotoxicty | Cardiotoxicity not pre-defined ( observed cardiac rhythm disorders & ST depression) | CAP | 16 | 2 |  |  |  |  |  |  | 29 months | the 2 cases were transient and resolved |
|  |  |  | CEP | 16 | 0 |  |  |  |  |  |  |  |  |
|  |  |  | CP | 16 | 0 |  |  |  |  |  |  |  |  |
| Levine 2005 [26] | CHF | MUGA | CEF | 351 |  | 4 |  |  |  |  |  | 60 months | CHF occurred within 5 years of follow-up, unclear if on or off treatment |
|  |  |  | CMF | 359 |  | 1 |  |  |  |  |  |  |  |
| Martin 2003 [27] | Cardiac outcomes | WHO CTC (0-4) | FAC | 505 | 11 |  |  |  |  |  |  | 77.7 months | 8 (grade 1-2)  3 (grade 3) |
|  |  |  | CMF | 480 | 1 |  |  |  |  |  |  |  | 1 (grade 1-2)  0 (grade 3), probably early and on therapy |
| Sposto 2001 [29] | Deaths | Cardiac related late (during long-term follow-up) | Daunomycin-COMP | 135 |  |  |  |  | 3 |  |  | 10 years | 3 cardiac related deaths off treatment and during long term follow up; due to: CHF, diffuse coronary heart disease awaiting heart transplant, collapsed during sport |
|  |  |  | COMP | 149 |  |  |  |  | 0 |  |  |  |  |
| Sweetnam 1986 [31] | Cardiotoxicity | clinical and sub clinical, ECG | Doxorubicin  Vincristine  Methotrexate | 95 | 13 | 1 |  |  | 2 | 10 |  | 5 years | ECG changes - 4 (changed treatment) 6 no change in treatment, deaths due to cardiomyopathy, off treatment, CHF off treatment, ECG changes on treatment |
|  |  |  | Vincristine  Methotrexate | 99 | 0 | 0 |  |  | 0 | 0 |  |  |  |
| Sullivan 1991 [30] | Death | Cardiac related late (8-10 years follow-up) | A-COPP + radiotherapy | 39 |  |  |  |  | 2 |  |  | 10 years | 1 death due to hypertension, 1 due to cardiopathy, 1 cardiac abnormality under treatment (late and off-treatment) in both groups |
|  |  |  | MOPP + bleomycin + radiotherapy | 45 |  |  |  |  | 0 |  |  |  |  |

Table 4b: anthracycline chemotherapy versus mitoxantrone

| **Study** | **Cardiac outcomes** | **Definition and measurement** | **Group** | **Number analysed** | **Cardiotoxic event** | **CHF** | **ECG alterations** | **Decrease in LVEF** | **Death (cardiac related)** | **Length of follow up** | **Additional comments, and when outcomes occurred** |
| --- | --- | --- | --- | --- | --- | --- | --- | --- | --- | --- | --- |
| Alonso 1995 [32] | LVEF  CHF | LVEF reduction to < 45%, clinical cardiotoxicity defined as any symptomatic dysfunction that required therapy | CAF | 50 |  | 6 |  | 2 |  | 5 years | Probably on treatment, could be late or off treatment |
|  |  |  | CMF | 50 |  | 3 |  | 3 |  |  |  |
| Aviles 1994 [45] | Cardiac toxicity | reduction LVEF >10% | EVBD | 35 |  | 0 |  | 1 |  | Median 36 months | no clinical evidence CHF, may include early and late, unclear if on or off, probably on treatment |
|  |  |  | MVBD | 33 |  | 0 |  | 4 |  |  |  |
| Bennett 1988 [33] | Reduction LVEF  CHF | Reduction LVEF by 15% to ≤45% (moderate), LVEF <30% (severe) MUGA, ECG, physical | CAF | 162 |  | 2 |  | 5 |  | > 1 year | symptoms of CHF resolved in all 3 patients after treatment  on treatment, probably early but may include >1 year |
|  |  |  | CNF | 163 |  | 1 |  | 1 |  |  |  |
| Cavo 2002 [43] | cardiac toxicity | WHO criteria grade 3 or 4 | VAD/MP | 174 | 9 |  |  |  |  | Median 31.5 months | cardiovascular events included CHF (14), unstable angina (2), MI (1), dns which groups, unclear if early or late or on or off treatment |
|  |  |  | VND/MP | 174 | 6 |  |  |  |  |  |  |
| Cook 1996 [34] | Cardiotoxicity | Clinical assessment of cardiac failure | Epirubicin | 18 | 3 |  |  |  |  | Unclear | 2 cardiac failures were cardiomyopathy, during or soon after long courses of epirubicin |
|  |  |  | Mitoxantrone | 22 | 0 |  |  |  |  |  |  |
| Esteban 1999 [35] | Cardiotoxicity | Moderate or severe LVEF disease according to Alexandre criteria or WHO grade 3 or 4 changes in cardiac rhythm and/or clinical instrumental signs of CHF | CEF |  | 7 | 1 | 3 |  |  | 5 years | Unclear if on or off treatment, only 80 patients tested for cardiotoxicity and unclear how many in each group |
|  |  |  | CNF |  | 8 | 2 | 3 |  |  |  |  |
| Follezou 1987 [36] | LVEF | Not clinically significant (moderate reduction) | CAF | 43 |  | 0 |  | 6 |  | Unclear |  |
|  |  |  | CNF | 43 |  | 0 |  | 2 |  |  |  |
| Gherlinzoni 1990 [44] | Cardiotoxicity | WHO CTC, LVEF mild, moderate or severe according to Alexandre criteria (echo) clinical and subclinical | m-BACOD | 35 |  |  |  | 6 |  | Unclear | 4 mild, 2 moderate (m-BNCOD)  8 mild (m-BNCOD) |
|  |  |  | m-BNCOD | 35 |  |  |  | 8 |  |  |  |
| Hausmaninger 1995 [37] | LVEF, CHF | moderate reduction LVEF (moderate not defined) | Epirubicin + vindesine | 129 |  | 0 |  | 5 |  | 3105 months | LVEF on treatment, late possible, 9% and 12% experienced transient and asymptomatic alterations in heart rhythm |
|  |  |  | Mitoxantrone + vindesine | 126 |  | 0 |  | 3 |  |  |  |
| Henderson 1989 [39] | LVEF, CHF | Reduction LVEF by 15% to ≤S45% (moderate), LVEF <30% (severe), ECG, MUGA or echo, physical | Doxorubicin | 154 |  | 5 |  | 11 |  | Unclear | LVEF reduction moderate for 8 in each group, early and on treatment as pre-crossover  Cumulative dose to CE (p=0.0005) |
|  |  |  | Mitoxantrone | 158 |  | 2 |  | 8 |  |  |  |
| Lawton 1993 [40] | cardiac complications  MI | not pre-specified: cardiac complications (cardiomyopathy & angina) | Doxorubicin | 28 | 1 |  |  |  | 1 | 6 weeks | Cause of death – MI  not clear if early or late, on or off treatment |
|  |  |  | Epirubicin | 28 | 1 |  |  |  | 0 |  |  |
|  |  |  | Mitoxantrone | 31 | 0 |  |  |  | 0 |  |  |
| Heidemann, 1993 [38] | LVEF  CHF | Toxicity assessed using WHO criteria  Exercise LVEF: reduction of >10%  ECG, MUGA or ECHO | Adriamycin  cyclophosphamide | 55 |  | 0 |  | 4 |  |  | ( LVEF shortening of less than 30%: 4, 4 and 5 for each group respectively)  Unclear when occurred |
|  |  |  | Epirubicin  Cyclophosphamide | 70 |  | 1 |  | 2 |  |  |  |
|  |  |  | Mitoxantrone  Cyclophosphamide | 63 |  | 0 |  | 6 |  |  |  |
| Pavlovsky 1992 [46] | Cardiac outcomes | toxicity criteria grade 1-4 | CHOP | 44 | 3 |  |  |  |  | Median 42 months | unclear when outcomes occurred, grade 1 (3) with CHOP, and grade 1 (2) and grade 4 (1) with CNOP |
|  |  |  | CNOP | 45 | 3 |  |  |  |  | Median 40 months |  |
| Periti 1991 [41] | Cardiotoxicity | Reduction in LVEF (grade 1-3) MUGA | FEC | 31 |  |  |  | 7 |  | Unclear | 7 (grade 1)  6 (5 grade 1, 1 grade 2)  Did not require treatment interruption  On therapy, early |
|  |  |  | FNC | 29 |  |  |  | 6 |  |  |  |
| Stewart 1997 [42] | Cardiac event, reduction in LVEF | Grade 3 or 4 cardiac events definite, probable possible or unknown relation to treatment,  LVEF reduction ≥10% MUGA | CAF | 128 | 1 |  |  | 21/56 |  | 5 years | unclear if on or off or late or early, potentially early as MUGA after max dose reached |
|  |  |  | CMF | 121 | 1 |  |  | 13/36 |  |  |  |

**Table 4c: anthracyclines given by bolus compared with continuous infusion**

| **Study** | **Cardiac outcomes** | **Definition and measurement** | **Group** | number analysed | Cardiotoxic event | discontinuation due to LVEF | CHF | cumulative dose to cardiotoxic event | decrease in LVEF | LVEF (%) pre mean (SD) | LVEF (%) post mean (SD) | death (cardiac related) | cardiotoxic event (clinically evident) | **Length of follow up** | **Additional comments, and when outcomes occurred** |
| --- | --- | --- | --- | --- | --- | --- | --- | --- | --- | --- | --- | --- | --- | --- | --- |
| Casper 1991 [49] | Cardiac toxicity   CHF | 10% or greater decrease in LVEF at rest by MUGA scan  CHF not defined | Bolus | 31 | 19 | 10 | 2 | log rank p= 0.002 | 19 |  |  | 1 |  | 50months | early and "during" treatment   CHF not subset of cardiotoxic event |
|  |  |  | Continuous | 38 | 16 | 2 | 2 |  | 16 |  |  | 1 |  |  |  |
| Zalupski, 1991 [50] | Cardiotoxicity Death | Cardiotoxicity according to SWOG criteria (worst grade) includes a reduction in LVEF, or clinically evident cardiac events, method not reported  Death cardiac related | Bolus | 117 | 15 |  |  |  | 6 |  |  | 2 | 9 | 5 years | Cardiotoxic event includes clinical and sub-clinical events, does not state whether early or late or on or off treatment |
|  |  |  | Continuous | 116 | 6 |  |  |  | 5 |  |  | 1 | 1 |  |  |
| Shapira 1990 [48] | Reduction LVEF  CHF | 20% or greater reduction in LVEF by MUGA  CHF not defined | Bolus | 28 | 13 |  | 4 |  | 13 | 0.6 (0.03) | 0.48 (0.05) |  |  | unclear | CHF subset of those with reduction LVEF  on therapy and early events |
|  |  |  | Continuous | 30 | 0 |  | 0 |  | 0 | 0.61 (0.03) | 0.58 (0.05) |  |  |  |  |
| Hortobagyi 1989 [47] | Reduction LVEF  CHF | 15% or greater reduction in LVEF, cardiac scan or ECHO  CHF not defined | Bolus | 23 |  |  | 3 |  | 3 | 69 | 67 |  |  | unclear | early and on treatment |
|  |  |  | Continuous | 27 |  |  | 1 |  | 1 | 68 | 61 |  |  |  |  |

**Table 4d: doxorubicin versus epirubicin**

| **Study** | **Cardiac outcomes** | **Definition and measurement** | **Groups** | number analysed | cardiotoxic event | decrease in LVEF | CHF | LVEF baseline value (%) | LVEF final value (%) | ECG abnormalities | Cardiac related death | Cardiac damage (Billingham  score 2.5 or higher) | PEP/LVET ratio | **Length of follow up** | **Additional comments, and when outcomes occurred** |
| --- | --- | --- | --- | --- | --- | --- | --- | --- | --- | --- | --- | --- | --- | --- | --- |
| Bezwoda 1986 [58] | Cardiotoxicity | Cardiotoxicity according to WHO criteria, MUGA scans | PEC | 24 | 0 |  | 0 |  |  |  |  |  |  | Unclear | Cardiotoxicity (grade 1), 2 (grade 2), 1 (grade 3)  CHF a subset of cardiotoxic event, controlled with medication, others had persistent asymptomatic reduction of LVEF 4-14 months after discontinuation. |
|  |  |  | PAC | 27 | 3 |  | 1 |  |  |  |  |  |  |  |  |
| Brambilla 1986 [53] | 1) Cardiac function  2) PEP/LVET ratio  3) LVEF | 1) Minor axis shortening (MAS) measured by ECG, 2) PEP/LVET ratio using Weissler technique  3) LVEF determined by radionuclide angiocardiography | Doxorubicin | 8 |  |  | 2 | 70.8 | 59.9 |  |  |  | pre (0.333 (se0.02)), post (0.383(se(0.02)) | Median 22 months | 18% on doxorubicin and 16% on epirubicin that had a specific and transient cardiotoxic event,  2 women started on doxorubicin had progressive decrease of LVEF and signs and symptoms of ventricular failure 6 and 14 months after treatment |
|  |  |  | Epirubicin | 8 |  |  | 0 | 69.1 | 64.1 |  |  |  | pre(0.344(se 0.013)), post (0.341(0.027)) |  |  |
| Bontenbal 1998 [52] | clinical CHF | CHF according to WHO CTC | Doxorubicin | 116 |  |  | 9 |  |  |  |  |  |  | Unclear | Could be early or late effects |
|  |  |  | Epirubicin | 113 |  |  | 2 |  |  |  |  |  |  |  |  |
| FESG, 1988  [51] | cardiac dysfunction | WHO criteria  no formal gradation of cardiotoxicity was attempted (investigators opinion on drug-related cardiotoxic events used). | FAC | 25 | 8 |  | 3 |  |  |  |  |  |  | 41 months | early and "during" treatment  CHF are sub set of cardiac dysfunction |
|  |  |  | FEC | 29 | 0 |  | 0 |  |  |  |  |  |  |  |  |
| Gasparini 1991 [54] | 1) Cardiotoxicity  2) ECG alterations | 1) WHO criteria  2) ECG abnormalties (acute arrhythmias) : ST-T segment depression, tachyarrhythmia | Doxorubicin | 21 | 1 |  |  |  |  | 3 |  |  |  | Unclear | EGC abnormalities were transient |
|  |  |  | Epirubicin | 22 | 0 |  |  |  |  | 2 |  |  |  |  |  |
| Heidmann 1993 [38] | Cardiotoxicity | Toxicity assessed using WHO criteria  1) LVEF: shortening of < 30%  2) Exercise LVEF: reduction of >10%  3)CHF  ECG and MUGA or echo | Adriamycin  cyclophosphamide | 55 |  | 4 | 0 |  |  |  |  |  |  | Unclear |  |
|  |  |  | Epirubicin  Cyclophosphamide | 70 |  | 2 | 1 |  |  |  |  |  |  |  |  |
| Homesley 1992 [59] | LVEF | Reduction LVEF >10%, ECG and MUGA | Cisplatin  Doxorubicin | 39 |  | 19 |  |  |  |  |  |  |  | Unclear |  |
|  |  |  | Cisplatin  Epirubicin | 33 |  | 3 |  |  |  |  |  |  |  |  |  |
| Hernadi 1988 [28] | cardiotoxicty | cardiotoxicity not pre-defined ( observed cardiac rhythm disorders & ST depression) | CAP | 16 | 2 |  |  |  |  |  |  |  |  | Median 29 months | Cardiotoxicity were transient and resolved |
|  |  |  | CEP | 16 | 0 |  |  |  |  |  |  |  |  |  |  |
| IMBSWE, 1988 [57] | cardiotoxicity | PEP:LVET ratio > 0.39, WHO grades 3 and 4 alterations of cardiac rhythm, and clinical and/or instrumental signs of heart failure by ECG | FAC | 247 | 53 |  | 4 |  |  | 53 |  |  | 21/ 119 | Median 473 days | median duration of follow up was 473 days for FAC and 500 days for FEC, early and "during" treatment |
|  |  |  | flurouracil, cyclophosphamide, epirubicin  (FEC) | 250 | 31 |  | 1 |  |  | 31 |  |  | 8/122 |  |  |
| Jain 1995 [55] | cardiac toxicity | decrease resting LVEF > 10% or decrease in stress LVEF > 5%, MUGA scan and clinical evaluation | Doxorubicin | 18 | 9 | 9 | 5 |  |  |  |  |  |  | Unclear | on treatment, early, not clear if CHF subset of cardiotoxic group |
|  |  |  | Epirubicin | 15 | 8 | 8 | 4 |  |  |  |  |  |  |  |  |
| Lahtinen 1991 [60] | 1) LVEF  2) CHF | 1) LVEF: 10% decrease  ECG, LVEF by radionuclide angiography | Cyclophosphamide  Doxorubicin  Vincristine  Prednisolone | 12 |  | 7 | 1 | 61 | 49 |  |  |  |  | Unclear | on treatment and early |
|  |  |  | Cyclophosphamide  Epirubicin  Vincristine  Prednisolone | 12 |  | 4 | 0 | 62 | 61 |  |  |  |  |  |  |
| Lawton 1993 [40] | cardiac complications,  MI | cardiac complications (cardiomyopathy & angina) | Doxorubicin | 28 | 1 |  |  |  |  |  | 1 |  |  | 6 weeks | not clear is early or late, on or off treatment, death due to MI off treatment and after surgery |
|  |  |  | Epirubicin | 28 | 1 |  |  |  |  |  | 0 |  |  |  |  |
| Perez 1991 [56] | 1) LVEF  2) CHF | LVEF reduction > 10% | Doxorubicin | 41 |  | 7 | 1 |  |  |  |  |  |  | Minimum 12 months | all 3 CHF were controlled by medication,  on and early |
|  |  |  | Epirubicin | 39 |  | 5 | 2 |  |  |  |  |  |  |  |  |

**Table 4e: liposomal doxorubicin versus non-liposomal doxorubicin or epirubicin**

| **Study** | **Cardiac outcomes** | **Definition and measurement** | **Groups** | number analysed | Cardiotoxic event | LVEF baseline value (%) | LVEF final value (%) | EF reduction (n) | LVEF reduction (%) | CHF (n) | Cardiac event | Billingham score >=2.5 | Death | **Length of follow up** | **Additional comments, and when outcomes occurred** |
| --- | --- | --- | --- | --- | --- | --- | --- | --- | --- | --- | --- | --- | --- | --- | --- |
| Batist 2001 [61] | 1) cardiotoxicity (primary end point)  2) cardiac event | 1) Decrease in resting LVEF of ≥ 20 ejection fraction units from baseline to a final value of ≥50%, or a decrease of ≥10 EF units from baseline to a final value of less than 50%, or clinical evidence of CHF (MUGA)  2) No definition reported | Liposome-encapsulated doxorubicin (Myocet) + cyclophosphamide (MC) | 142 | 9 |  |  |  |  | 0 | 6 |  |  | Median 20 months | Events are mix of "early & late" and "during" treatment, median follow up 20 months |
|  |  |  | Doxorubicin + cyclophosphamide (AC) | 155 | 33 |  |  |  |  | 5 | 34 |  |  |  |  |
| Harris 2002 [62] | 1) cardiac toxicity  2) reduction in LVEF  3) cardiac damage | 1) Decrease in resting LVEF of 20 or more from baseline to a final value of≥ 50%, a decrease of≥ to 10 points from baseline to a final value of < 50%, a cardiac biopsy of Grade 2.5 or higher or clinical evidence of CHF  2) S/A  3) Billingham score derived from endomyocardial biopsies.score≥ 2.5 | Liposome-encapsulated doxorubicin (TLC D-99) | 108 | 14 |  |  | 12 |  | 2 |  | 5 /19 | 0 | Unclear | Death due to CHF and drug related  2 CHF on TLCD, 3 doxorubicin off- treatment, 6 on- treatment doxorubicin, all early |
|  |  |  | Doxorubicin | 116 | 34 |  |  | 25 |  | 9 |  | 12/17 | 1 |  |  |
| O’Brien 2004 [63] | 1) cardiac event  2) CHF | 1) decrease ≥20% from baseline LVEF in normal range or ≥10% decrease in LVEF in abnormal range (MUGA)  2) clinical signs and symptoms such as dyspnea upon excertion, peripheral edema, orthopnea or tachypnea requiring treatment. | Pegylated liposomal doxorubicin (PLD) | 254 | 10 |  |  | 10 | 2.3 | 0 |  |  |  | Unclear | CHF are a subset of the cardiotoxic events, cardiotoxic events are the same cases with LVEF, all on treatment |
|  |  |  | Doxorubicin | 255 | 48 |  |  | 48 | 11.6 | 10 |  |  |  |  |  |
| Rifkin 2006 [64] | 1) cardiac adverse events  2) LVEF reduction | 1) CTC for adverse events (CHF grade 3 or 4) MUGA and echo | Pegylated liposomal doxorubicin (DVd) | 97 |  |  |  | 1* | 2.3 | 0 |  |  |  | 21 months | LVEF reduction p=0.01 favouring liposomal, no thromboembolic deaths, *discontinuation due to LVEF reduction |
|  |  |  | doxorubicin (VAd) | 95 |  |  |  | 4* | 4.5 | 2 |  |  |  | 20 months |  |
| Chan, 2004 [65] | Cardiotoxicity | Decrease in LVEF of ≥20 ejection fraction units from baseline, or a decrease of ≥10 EF units from baseline to a final value of less than 50% or clinical evidence of CHF (ECG) | Liposomal doxorubicin (Myocet) + cyclophosphamide  (MC) | 76 | 9 |  |  | 9 |  | 0 |  |  |  | 21 months | All cardiotoxic events are LVEF events, no CHF events  Unclear if early/ late or on/off treatment, median follow up 21 months |
|  |  |  | Epirubicin + cyclophosphamide  (EC) | 78 | 8 |  |  | 8 |  | 0 |  |  |  |  |  |
|  |  |  | Cyclophosphamide  Idarubicin  Vincristine  Prednisolone | 27 | 3 | 62 | 59 |  |  |  |  |  |  |  |  |

**Table 4f: cardioprotective agents**

| **Study** | **Outcomes** | **Definition and measurements** | **Group** | **number analysed** | **cardiotoxic event** | **CHF** | **NYHA II symptoms** | **NYHA III to V** | **Ejection fraction decreased** | **LVIDD (mm) pre mean (SD)** | **LVIDD (mm) post mean (SD)** | **LVIDD (mm) 6 months mean (SD)** | **EF (%) pre mean (SD)** | **EF (%) (mm) post mean (SD)** | **EF (%) (mm) 6 months mean (SD)** | **LVEF baseline value (SD) (%)** | **LVEF final value (SD) (%)** | **death (cardiac related)** | **Additional comments, and when outcomes occurred** | **Length of follow up** |
| --- | --- | --- | --- | --- | --- | --- | --- | --- | --- | --- | --- | --- | --- | --- | --- | --- | --- | --- | --- | --- |
| Lopez, 1998 [73] | 1) Clinical cardiotoxicity  2) CHF  3) Laboratory cardiotoxicity | 1) clinical signs of cardiac toxicity (NYHA)  2)CHF  3) Laboratory cardiotoxicity defined as a LVEF <45% or a decrease from baseline by 20% (MUGA) | dexrazoxane | 59 | 4 | 0 | 4 | 0 | 5 |  |  |  |  |  |  | 64 (65) | 65 (8.9) |  | CHF off-treatment, resolved with medication  2 patients withdrawn epirubicin, I angina, 1 AV block | Unclear |
|  |  |  |  | 62 | 15 | 4 | 9 | 4 | 18 |  |  |  |  |  |  | 65 (6.8) | 57 (8.7) |  |  |  |
| Marty, 2006 [68] | 1) Cardiac event  2) Time to cardiac event-free survival  3) Ejection fraction decrease | Cardiac event: Reduction in LVEF by 10% (MUGA) or 15% (echo) or value below 45%, or clinical signs of cardiac insufficiency (NYHA grade 2/3/4).  Ejection fraction decrease (CTC grade 3/4) | 20:1 dextrazoxane:doxorubicin dose ratio, or 10:1 dexrazoxane: epirubicin dose ratio. | 79 | 10 | 1 | 1 | 0 | 9 |  |  |  |  |  |  |  |  |  | early & during treatment  CTC EF reduction also reported | Unclear |
|  |  |  | doxorubicin or epirubicin | 74 | 29 | 8 | 1 | 7 | 21 |  |  |  |  |  |  |  |  |  |  |  |
| Speyer, 1992 [69] | Cardio toxicity, death due to cardiac causes | NYHA 2,3, 4 clinical cardiotoxicity, subclinical cardiotoxicity NYHA 1, decrease in LVEF by at least 20% or to <45% (MUGA) or Billingham score at least 2 (biopsy) | dexrazoxane | 76 | 6 |  | 2 | 0 |  |  |  |  |  |  |  |  |  | 1 | Deaths one each on/off study  Cardiotoxicity on treatment | Unclear |
|  |  |  |  | 74 | 37 |  | 10 | 10 |  |  |  |  |  |  |  |  |  | 1 |  |  |
| Swain, 1997 [70] | 1) cardiac event (including CHF) 2) CHF | 1) cardiac event defined as reduction in LVEF by at least 10% below LLN, or reduction at least 20% from baseline, or to at least 5% below LLN 2) CHF any 2 of following (cardiomegaly established by radiography, basilar rales, S3 gallop, paroxysmal nocturnal dyspnea, orthopnea or sig dyspnea on exertion  ECG, resting LVEF by MUGA | dexrazoxane | 249 | 36 | 2 |  |  |  |  |  |  |  |  |  |  |  | 0 | 11/1 off treatment, log rank p-value < 0.001 and 0.038 for each study for cumulative dose to | Median 532 days (study 1) and 397 days (study 2) |
|  |  |  | Placebo | 285 | 89 | 22 |  |  |  |  |  |  |  |  |  |  |  | 2 |  | Median 511 days (study 1) and 517 (study 2) |
| Venturini, 1996 [71] | cardio toxicity | Cardiotoxicity defined as clinical CHF classified as NYHA grade2,3,4 or reduction in LVEF by MUGA to <=45%, or reduction from baseline resting LVEF of >=20 EF units | dexrazoxane 10:1 | 82 | 6 | 2 | 2 | 0 | 4 |  |  |  |  |  |  |  |  |  | Cardiotoxicity includes clinical and sub-clinical events | Unclear |
|  |  |  | Placebo | 78 | 18 | 4 | 1 | 3 | 14 |  |  |  |  |  |  |  |  |  |  |  |
| Wexler 1996 [72] | Cardiac toxicity | Dose limiting cardiotoxicity defined as reduction in LVEF below 45% (lower limit of normal) or decrease > 20% or clinical CHF  MUGA scan | Doxorubicin + dexrazoxane 20:1 | 18 | 4 |  |  |  |  |  |  |  |  |  |  |  |  |  | on therapy, early  Cardiotoxic events includes both clinical and sub-sclinical | 39 months |
|  |  |  | Doxorubicin | 14 | 10 |  |  |  |  |  |  |  |  |  |  |  |  |  |  | 40 months |
| Waldner, 2006 [75] | Size of left ventricle (LVIDD), systolic function (EF) and E/A ratio | Echocardiograph | L-cartinine | 20 |  |  |  |  |  | 58.3 (5.9) | 49.9 (5.0) | 52.0 (5.1) | 61.0 (6.0) | 58.3 (5.9) | 59.4 (7.4) |  |  |  | Early and on treatment and off treatment | 6 months |
|  |  |  | Placebo | 20 |  |  |  |  |  | 51.7 (4.1) | 51.4 (5.6) | 51.4 (5.6) | 61.9 (6.3) | 60.0 (6.2) | 60.3 (6.5) |  |  |  |  |  |
| Kalay, 2006 [74] | systolic function | systolic dysfunction defined as EF < 50%, ECG | Carvedilol | 25 |  |  |  |  | 1 |  |  |  |  |  |  | 70.5 | 70 |  | Early and on treatment | 6 months |
|  |  |  | Placebo | 25 |  |  |  |  | 5 |  |  |  |  |  |  | 69 | 52 |  |  |  |
| Milei, 1987 [76] | 1) Cardiotoxic event (myocardiopathy or rhythm disturbances)  2) CHF | 1) Myocardiopathy included: diastolic and systolic diameter, shortening fraction, septal moyility, left atrial size, mitral septal distance.  2) CHF: clinical assessment  Chest X-ray | Prenylamine | 13 | 0 | 0 |  |  |  |  |  |  |  |  |  |  |  |  | CHF subset of cardiotoxic event, rhythm disturbance is other event resolved with treatment  Early and on treatment | 5.9 months |
|  |  |  | Placebo | 13 | 3 | 2 |  |  |  |  |  |  |  |  |  |  |  |  |  |  |
| Gallegos-Castorena 2007 [77] | cardiac toxicity | WHO CTC grade 1,2,3,4 | Amifostine | 15 | 0 |  |  |  |  |  |  |  |  |  |  |  |  |  | Early and on -therapy | Unclear |
|  |  |  |  | 13 | 2 |  |  |  |  |  |  |  |  |  |  |  |  |  |  |  |
| Myers 1983 [78] | CHF | CHF not defined, ECG-gated pool scans | Acetylcysteine | 17 |  | 3 |  |  |  |  |  |  |  |  |  |  |  |  | on therapy early, only patients staying on therapy analysed | Unclear |
|  |  |  |  | 14 |  | 3 |  |  |  |  |  |  |  |  |  |  |  |  |  |  |

Abbreviations: A-COPP=cyclophosphamide, vincristine, prednisone, procarbazine; CAF=cyclophosphamide, adriamycin, 5-FU; CEF=cyclophosphamide, epirubicin, 5-FU; CMF=cyclophosphamide, methotrexate, 5-FU; CAP=cyclophosphamide, cisplatin, adriamycin; CEP=cyclophosphamide, cisplatin, epirubicin; CP=cyclophosphamide, cisplatin; CHF=congestive heart failure; CHOP=cyclophosphamide, vincristine, doxorubicin, prednisone; COMP=cyclophosphamide, vincristine, methotrexate, prednisone; CNOP=cyclophosphamide, vincristine, mitoxantrone, prednisone; CMF=cyclophosphamide, fluorouracil, mitoxantrone; CNF=cyclophosphamide, 5-FU, mitoxantrone; ECG=Electrocardiogram; ECOG=European Cooperative Oncology Group; EVBD=epirubicin, vinblastine, bleomycin; MVBD=mitoxantrone, vinblastine, bleomycin; FAC=5-FU, cyclophosphamide, doxorubicin; FEC=5-FU, cyclophosphamide, epirubicin; FNC=cyclophosphamide, 5-FU, mitoxantrone; LVEF=Left Ventricular Ejection Fraction; LLN=lower limit of normal; MI= myocardial infarction; MOPP=mustargen, vincristine, prednisone, procarbazine; M-BACOD=cyclophosphamide, bleomycin, vincristine, dexamethazone, methotrexate, leucovorin, doxorubicin; m-BNCOD=cyclophosphamide, bleomycin, vincristine, dexamethazone, methotrexate, leucovorin, mitoxantrone; MUGA=multigated acquisition scan; PAC=Cisplatin, adriamycin, cyclophosphamide; PEC=Cisplatin, 4'epi-adriamycin, cyclophosphamide; PEP/LVET=left ventricular systolic time interval ratios; SD=standard deviation; VAD=vincristine, doxorubicin, dexamethazone; VND/MP= vincristine, mitoxantrone, dexamethazone; WHO-CTC=World Health Organization common toxicity criteria
